# Yeast Growth Plasticity Is Regulated by Environment-Specific Multi-QTL Interactions

**DOI:** 10.1534/g3.113.009142

**Published:** 2014-01-28

**Authors:** Aatish Bhatia, Anupama Yadav, Chenchen Zhu, Julien Gagneur, Aparna Radhakrishnan, Lars M. Steinmetz, Gyan Bhanot, Himanshu Sinha

**Affiliations:** * Department of Physics and Astronomy, Rutgers University, Piscataway, New Jersey 08854; † Department of Biological Sciences, Tata Institute of Fundamental Research, Mumbai 400005, India; ‡ European Molecular Biology Laboratory, Genome Biology Unit, 69117 Heidelberg, Germany; § Gene Center, Ludwig-Maximilians-Universität, 81377 Munich, Germany; ** Department of Genetics, Stanford University School of Medicine, Stanford, California 94305; †† Stanford Genome Technology Center, Stanford University, Palo Alto, California 94304; ‡‡ BioMaPS Institute for Quantitative Biology, Rutgers University, Piscataway, New Jersey 08854; §§ Department of Molecular Biology and Biochemistry, Rutgers University, Piscataway, New Jersey 08854; *** Rutgers Cancer Institute of New Jersey, New Brunswick, New Jersey 08903; ††† Simons Center for Systems Biology, Institute for Advanced Study, Princeton, New Jersey 08540

**Keywords:** growth plasticity, carbon source variation, quantitative trait locus, gene–environment interaction, gene–gene interaction

## Abstract

For a unicellular, non-motile organism like *Saccharomyces cerevisiae*, carbon sources act both as nutrients and as signaling molecules and consequently affect various fitness parameters including growth. It is therefore advantageous for yeast strains to adapt their growth to carbon source variation. The ability of a given genotype to manifest different phenotypes in varying environments is known as phenotypic plasticity. To identify quantitative trait loci (QTL) that drive plasticity in growth, two growth parameters (growth rate and biomass) were measured in a published dataset from meiotic recombinants of two genetically divergent yeast strains grown in different carbon sources. To identify QTL contributing to plasticity across pairs of environments, gene–environment interaction mapping was performed, which identified several QTL that have a differential effect across environments, some of which act antagonistically across pairs of environments. Multi-QTL analysis identified loci interacting with previously known growth affecting QTL as well as novel two-QTL interactions that affect growth. A QTL that had no significant independent effect was found to alter growth rate and biomass for several carbon sources through two-QTL interactions. Our study demonstrates that environment-specific epistatic interactions contribute to the growth plasticity in yeast. We propose that a targeted scan for epistatic interactions, such as the one described here, can help unravel mechanisms regulating phenotypic plasticity.

The ability of a given genotype to exhibit different phenotypes in various environments is called phenotypic plasticity (Pigliucci 2001). This property is often observed in organisms that have adapted to varying environmental conditions. For a fixed genotype, plasticity can be quantified as the change in phenotype when the environment is varied (for a binary phenotype) or the slope of the phenotype–environment curve (for a continuous phenotype variable) (Scheiner 1993; Pigliucci 2005; Bergland *et al.* 2008). Plasticity can allow an organism to adapt to new environments as well as protect it from potentially adverse environmental effects (Via and Lande 1985).

A gene–environment interaction (GEI) occurs when the phenotypic effect of an allele is environment-dependent. The occurrence of such GEIs creates variation in phenotypic plasticity (Via and Lande 1985; Shook and Johnson 1999). Natural selection may then act on this variation to enhance or reduce the plasticity in a population. Several studies have identified roles of single genes affecting plasticity (Kent *et al.* 2009; Gerke *et al.* 2010). Many of these fitness phenotypes affected by the environment are quantitative in nature and are affected by interactions between various alleles (Mackay 2001; Remold and Lenski 2004). Furthermore, single allele effects do not account for all observed variation in such traits (Fijneman *et al.* 1996; Bloom *et al.* 2013). Hence, to gain insights into genomic regulation of plasticity, it is important to study both GEIs as well as environment-specific multi-locus interactions.

In a unicellular, sessile organism such as *Saccharomyces cerevisiae*, carbon sources act both as energy sources and as signaling molecules (Broach 2012). Carbon source availability affects basic biological processes, such as translation regulation, metabolism, and signaling pathways, which directly or indirectly affect yeast growth (Zaman *et al.* 2008). In its evolutionary history, yeast would have encountered and adapted to both fermentable and high growth sources such as glucose, fructose, maltose, as well as non-fermentable and slow growth sources such as glycerol and ethanol (Gancedo 1998; Broach 2012).

Yeast growth has previously been studied by measuring different growth phenotypes, such as colony size (Tong *et al.* 2001, 2004), biomass (Perlstein *et al.* 2006), and growth kinetics (Warringer *et al.* 2003, 2011). These different measures of growth are driven by a partially overlapping set of genes across varied environmental conditions (Warringer *et al.* 2008; Cubillos *et al.* 2011). To understand the genetic basis of growth phenotype plasticity, it is therefore important to quantify growth by multiple growth parameters over a range of carbon sources, because these parameters can have distinct regulatory mechanisms.

Previously, several linkage mapping studies have identified genetic loci that contribute to the variation of quantitative traits in different carbon sources (Warringer *et al.* 2008; Cubillos *et al.* 2011; Bloom *et al.* 2013). These studies identified numerous QTL, as well as epistatic interactions among QTL that contribute to variation in growth in different carbon sources. While there is a well-developed understanding of how QTL contribute to growth differences in a given environment (Sinha *et al.* 2008; Cubillos *et al.* 2011; Ehrenreich *et al.* 2012; Yang *et al.* 2013; Gagneur *et al.* 2013), the role of QTL–environment interactions and the environment dependence of epistatic interactions governing growth are not very well-studied. Several studies have either taken a specific pair of environmental variations or have considered a small set of QTL contributing to phenotypic variation (Sambandan *et al.* 2008; Gerke *et al.* 2010). While some of these studies do consider multiple measures of growth (Warringer *et al.* 2008; Cubillos *et al.* 2011), differential genetic regulation of growth parameters is still not well-understood. Similarly, the role of multi-locus interactions on such regulation and the environment-dependence of these regulatory interactions are not clear. In this study, we attempt to address these questions by identifying QTL, QTL–environment interactions, and two-QTL and three-QTL interactions that contribute to variation in growth across a varied set of carbon sources.

In a set of 144 high-resolution genotyped meiotic segregants (Mancera *et al.* 2008) of two genetically and phenotypically divergent yeast strains grown separately in the presence of ethanol, fructose, glucose, glycerol, lactose, maltose, or sucrose as the sole carbon source, we measured two growth parameters: growth rate (doubling time) and biomass accumulated (maxOD) in a published dataset (Gagneur *et al.* 2013). QTL were mapped independently in each environment for both growth parameters. This was followed by GEI mapping, which identified loci where the plasticity (*i.e.*, change in phenotype with respect to environment) differs for the two genotypes.

As a large number of genetic factors interact to affect growth, many QTL involved in epistatic interactions may have a small independent effect. The large sample sizes and computational resources required for genome-wide epistatic analyses have made identification of these interactions difficult (Phillips 2008). To identify epistatic interactions with a limited sample size, we searched for interactions among a targeted set of candidate loci, namely those QTL that showed an effect of any size and QTL that showed GEI. The reason for including the latter set was that many QTL whose effect in a single environment does not exceed the threshold for genome-wide significance may have opposite effects in different environments, and thus can be identified in a GEI scan. The approach that we used for this article allows us to identify epistatic interactions among small-effect QTL (either single-environment or GEI QTL) with a relatively small population size (144 segregants). We were able to identify two loci indicating novel interactions as well as a greater role for known single-environment QTL in regulating growth. Our study showed that yeast growth is regulated by such environment and growth parameter–specific multi-locus interactions.

## Materials and Methods

### Growth data

The raw growth data analyzed in this study were derived from a study by Gagneur *et al.* (2013), in which the experimental procedures are described in detail. The data we used were generated for 157 segregants derived from a cross between *S. cerevisiae* strains S96 and YJM789 (Mancera *et al.* 2008) grown in the following conditions: 2% ethanol; 2% fructose; 2% glucose; 3% glycerol; 2% lactose; 2% maltose; and 2% sucrose. The filtered phenotype and genotype data as well as all necessary files for analysis are available in Supporting Information, File S1 and File S2.

### Mapping single-environment QTL

For each strain, two growth parameters (doubling time and biomass) were measured in seven environmental conditions (ethanol, fructose, glucose, glycerol, lactose, maltose, and sucrose). Genotype data for the parental strains and segregants were obtained (Mancera *et al.* 2008) and filtered to include only single nucleotide markers, which resulted in 48,934 markers.

The R/qtl package (Broman *et al.* 2003; Broman and Sen 2009) was used to construct a genetic map and to identify QTL separately for each growth parameter in each of the seven environmental conditions. QTL were identified using the LOD score, which is the log_10_ of the ratio of the likelihood of the experimental hypothesis to the likelihood of the null hypothesis (Broman and Sen 2009).

An interval mapping method (“scanone” function in R/qtl) was used to compute this LOD score using the Haley-Knott regression algorithm (Broman *et al.* 2003) (see File S1 for details). This method had an advantage over marker regression in that it can impute data at missing markers and inspect positions between markers. *P* values were computed in R/qtl with a permutation test (1000 permutations) where the null distribution consisted of the highest genome-wide LOD score obtained from each permutation (Broman *et al.* 2003). For R scripts for this and other QTL mapping, see File S2.

### Mapping GEI

GEI occurs when the effect of a QTL is environment-dependent. Such QTL were identified by pooling data from two environmental conditions and including the effect of the environment as a covariate. The LOD scores were calculated using the “scanone” function in R/qtl (using the Haley–Knott regression algorithm) (Broman *et al.* 2003), including the environmental variable as an additive and interactive covariate (see File S1 for details). *P* values were computed in R/qtl with a permutation test (100 permutations).

### Mapping multi-QTL interactions

A two-QTL (QTL-QTL) and three-QTL interaction occurs when the QTL effect at a single locus depends on the genotype at another locus (see File S1 for details). For targeted multi-QTL interaction mapping, markers from both the growth parameters identified in single-environment (*P* < 1) and GEI (*P* < 0.5) QTL mapping were collated. This combined set of markers was tested for both parameters. A custom python script was written to compute this LOD score for pairwise comparisons among a set of markers (see File S1 and File S2 for details). *P* values were computed using a permutation test (10,000 permutations).

## Results

### Mapping previously identified and novel QTL

Growth rate (doubling time) and biomass accumulated (maxOD) were measured for parental haploid strains S96 (indicated as S) and YJM789 (indicated as Y) and their haploid meiotic segregants separately in the presence of seven different carbon sources: five fermentable (glucose, fructose, sucrose, maltose, lactose) and two non-fermentable carbon sources (ethanol and glycerol; see *Materials and Methods*).

For the two growth phenotypes, phenotypic correlations between different environmental conditions ranged from −0.02 to 0.63 (Pearson correlation coefficient) (Table S1). For easily utilizable carbon sources (glucose, fructose, and sucrose), the segregants showed higher correlation for doubling time (ranging from 0.41 to 0.53) but a comparatively lower correlation for maxOD (ranging from 0.12 to 0.27). This suggested a distinct genetic basis for regulation of growth in different environments, and this hypothesis was later supported by our findings.

**Figure 1:**
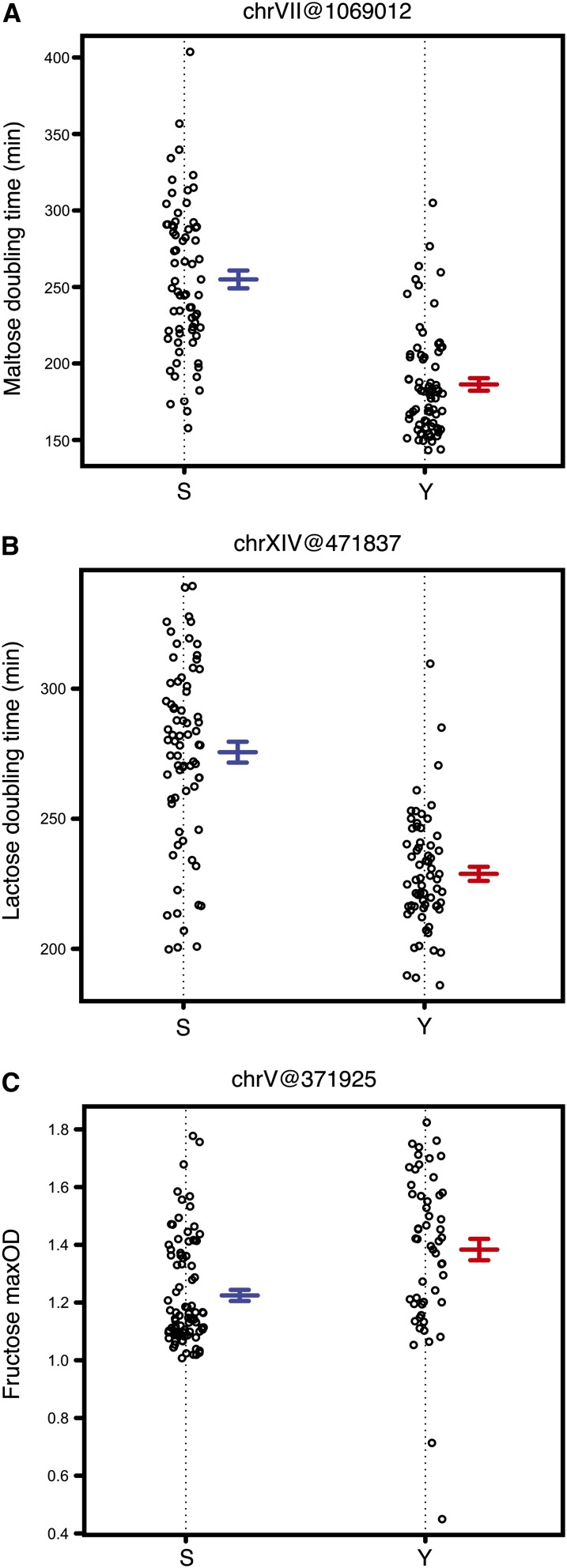
Scatter plots showing examples of QTL identified for various growth parameters and environmental conditions. (A, B, C) The QTL is indicated as chromosome number followed by marker position in bp within brackets. The x-axis indicates marker genotype. Error bars indicate ± 1 SE.

QTL mapping was performed in all conditions for doubling time and maxOD. Many QTL (with *P* ≤ 0.2) were identified for both the growth parameters in different environmental conditions (Table 1, Figure S1). A chrII(516,338) QTL, containing the *AMN1* gene [also identified in the analysis of this data by Gagneur *et al.* (2013)] was mapped for doubling time in glucose. The same locus was also mapped for fructose and sucrose, the other two highly fermentable sugars (Table 1), consistent with the observed high correlation for doubling time of the segregants between these fermentable carbon sources. Apart from being identified in glucose and maltose (Gagneur *et al.* 2013), the chrXIV QTL, containing the gene *MKT1*, was also mapped for lactose doubling time (Figure 1A). Because the S strain has a *mal13* mutation (Charron *et al.* 1986), the chrVII locus containing *MAL13* gene was identified as a regulator of both doubling time (Figure 1B) and maxOD in maltose (Figure S1). A locus on chrV was also mapped, which affected fructose maxOD (Figure 1C), and was later found to be involved in various epistatic interactions in different media conditions (see *Results* below). Apart from the chrVII QTL containing *MAL13*, no common loci was identified for both growth parameters (Table 1). This again was consistent with the correlation analysis and with previous studies (Warringer *et al.* 2008; Cubillos *et al.* 2011, 2013).

**Table 1:** Environment-specific QTL

Parameter	Media	Single QTL (*P* ≤ 0.2)	Two-QTL Interactions*^a^* (*P* ≤ 0.2)	Two-QTL Interactions with Large-Effect QTL*^b^* (*P* ≤ 0.2)	Three-QTL Interactions*^c^* (*P* ≤ 0.2)
**Doubling time**	Ethanol	chrXIV(465,189), 9.48, 0.001	chrX(68,089)-chrXVI(298,954), 3.06, 0.014		
	Fructose	chrII(558,465), 5.82, 0.001; chrXIII(26,435), 4.14, 0.012			
	Glucose	chrII(516,338), 4.56, 0.004; chrXIV(491,256), 3.70, 0.04	chrI(33,865)-chrV(377,186), 2.44, 0.052	chrV(371,899)-chrI(33,865), 2.265, 0.087	
	Glycerol	chrXIV(441,202), 1.03, 0.08		chrV(371,899)-chrXIII(715,970), 1.28, 0.163	
	Lactose	chrXIV(467,221), 16.05, 0.001			
	Maltose	chrVII(1,069,012), 17.68, 0.001; chrXV(656,568), 3.04, 0.18	chrIX(55,251)-chrXIII(555,077), 2.70, 0.053		
	Sucrose	chrII(516,338), 3.50, 0.036	chrV(371,899)-chrXV(473,018), 2.36, 0.125	chrV(371,899)-chrXV(473,018), 2.36, 0.08	chrI(62,951)-chrV(371,899)-chrV(525,070), 3.02, 0.189; chrII(516,338)-chrV(371,899)-chrV(525,070), 3.02, 0.188
**maxOD**	Ethanol	chrV(525,070), 4.60, 0.006			chrII(558,465)-chrVII(42,563)-chrXIII(26,435), 3.32, 0.078
	Fructose	chrV(371,899), 3.72, 0.039		chrV(371,899)-chrIX(313,896), 2.29, 0.126	
	Glucose		chrI(33,865)-chrV(377,186), 2.15, 0.111; chrV(377,186)-chrXV(656,568), 2.29, 0.078	chrI(33,865)-chrV(371,899), 2.19, 0.104; chrV(371,899)-chrXV(653,770), 1.90, 0.197; chrV(371,899)-chrXV(656,568), 2.63, 0.035	chrII(558,465)-chrV(377,186)-chrVIII(145,761), 3.07, 0.097
	Glycerol			chrV(525,070)-chrIX(420,785), 1.98, 0.184	
	Lactose	chrIII(56,309), 3.03, 0.151			
	Maltose	chrVII(1,069,000), 26.84, 0.001			chrII(182,131)-chrV(371,899)-chrXIII(555,077), 3.11, 0.185
	Sucrose		chrI(62,951)-chrV(371,899), 3.76, 0.007; chrV(371,899)-chrXV(473,018), 3.55, 0.009	chrI(62,951)-chrV(371,899), 3.76, 0.005; chrI(62,951)-chrV(525,070), 2.24, 0.075; chrV(371,899)-chrXV(473,018), 3.55, 0.006	chrV(371,899)-chrV(525,070)-chrXV(473,018), 3.13, 0.148

Each entry lists the chromosome, chromosome position in bp within brackets, LOD score, and *P* value for an identified QTL. Markers with *P* ≤ 0.2 are listed.

a Results of a pairwise scan of two-QTL interactions among single-environment and GEI QTL.

b Results of a targeted scan for two-QTL interactions where one locus is constrained to be a large-effect QTL.

c Results of a targeted scan for three-QTL interactions.

### Weak-effect QTL interact antagonistically in different carbon sources

**Figure 2:**
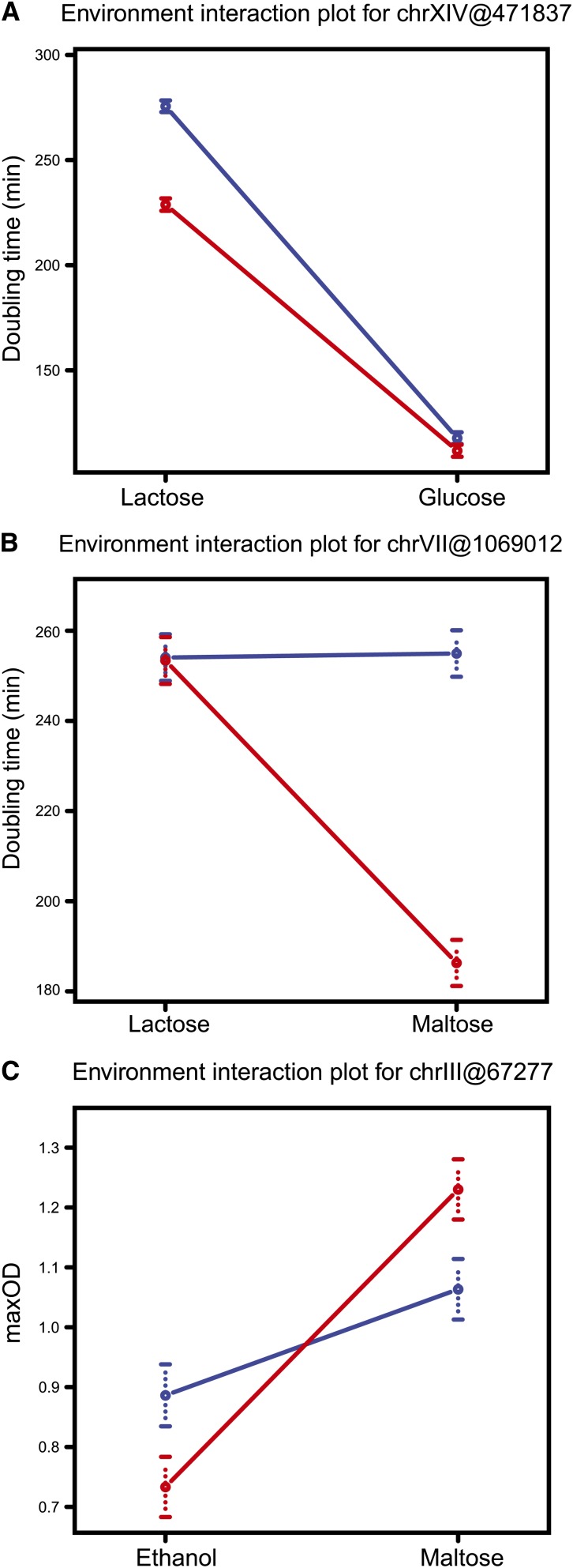
Reaction norms of three classes of GEI QTL. (A) Scale effect GEI QTL: mean doubling time for segregants grown in glucose and lactose, carrying either S (blue) or Y (red) allele at chrXIV(468,490) marker position. (B) Environment-specific GEI QTL: mean doubling time for segregants grown in glucose and maltose, carrying either S (blue) or Y (red) allele at chrVII(1,069,012) marker position. (C) Crossover effect GEI QTL: mean maxOD for segregants grown in lactose and ethanol, carrying either S (blue) or Y (red) allele at chrII(708,904) marker position. Error bars indicate ± 1 SE.

To identify environmental effects on QTL, mapping was performed using the environment as an interactive covariate between pairs of growth media (Table 2, Figure S2). Although there is no conventional way of differentiating scale and crossover GEI QTL, studies have used different methods, for example, comparing variance (Lacaze *et al.* 2009). We categorized the GEI QTL in the three classes on the basis of non-overlapping ± 1 SE. By mapping GEI between pairwise comparisons of growth media, three classes of GEI QTL were identified. The first was scale effect interactions, which occur when a single-environment QTL contributes to variation in both environments but with different magnitude. For example, a chrXIV QTL affected doubling time in both lactose and glucose but the polymorphism resulted in a larger growth rate difference in lactose (Figure 2A). Second was the environment-specific QTL, which contribute to growth in only one of the two media (to the limit of our mapping resolution), for example, the maltose-specific chrVII QTL affecting doubling time (Figure 2B). Third was the crossover effect QTL, where the parental alleles have effects in opposite directions on the growth parameters in the two environments, for example, a chrII QTL affecting maxOD in lactose and ethanol (Figure 2C). Most of these GEI QTL were either antagonistic or present only in one environment. We identified nine novel QTL, out of which seven were antagonistic. There were two scale effect GEI QTL for doubling time between lactose and glucose [chrXIV(468,480)] and ethanol and glucose [chrXIV(465,189)] (Table 2, Figure S2).

**Table 2:** Gene–environment interaction QTL

Doubling Time	Ethanol	Fructose	Glucose	Glycerol	Lactose	Maltose	Sucrose
Ethanol		chrXIV(465,189), 9.58, 0.01	chrXIV(465,189), 9.29, 0.01			chrVII(1,069,012), 13.01, 0.01	chrXIV(465,189), 9.16, 0.01
Fructose					chrXIV(467,221), 14.47, 0.01	chrVII(1,069,012), 20.63, 0.01; chrXV(656,568), 3.12, 0.2	
Glucose					chrXIV(468,490), 14.12, 0.01	chrVII(1,069,012), 20.49, 0.01	
Glycerol							
Lactose						chrVII(1,069,012), 16.44, 0.01	chrXIV(468,490), 13.28, 0.01
Maltose							chrVII(1,069,012), 20.07, 0.01; chrXV(656,568), 3.05, 0.2
Sucrose							

maxOD	Ethanol	Fructose	Glucose	Glycerol	Lactose	Maltose	Sucrose
Ethanol		chrV(377,186)*^a^*^,^*^b^*, 6.76, 0.01	chrV(377,186)*^a^*^,^*^b^*, 3.58, 0.1	chrV(377,186)*^a^*^,^*^b^*, 4.33, 0.02; chrVII(25, 442)*^a^*^,^*^b^*, 3.03, 0.17	chrII(708,904)*^a^*^,^*^b^*, 3.78, 0.02; chrXVI(295,943)*^a^*^,^*^b^*, 3.92, 0.01	chrVII(1,069,000), 28.25, 0.01	
Fructose						chrIII(248,850)*^a^*, 3.16, 0.13; chrVII(1,069,012), 21.09, 0.01; chrXV(288,114)*^a^*, 3.07, 0.14	chrV(371,899)*^a^*^,^*^b^*, 4.52, 0.01
Glucose						chrVII(1,069,012), 26.70, 0.01	
Glycerol						chrVII(1,069,000), 26.19, 0.01	
Lactose						chrVII(1,069,012), 24.85, 0.01	
Maltose							chrVII(1,069,000), 26.17, 0.01
Sucrose							

Each entry lists the chromosome, chromosome position in bp within brackets, LOD score, and *P* value for markers involved in a gene–environment interaction. Markers with *P* ≤ 0.2 are listed.

a Novel QTL that was not identified in concerned environments in environment-specific QTL mapping (see Table 1).

b QTL that had a crossover effect (antagonistic allelic effect with non-overlapping ± 1 SE bars).

Some of these QTL were below the genome-wide significance threshold in each individual environment but were identified as genome-wide significant GEI QTL when their effects between environments were considered. Thus, the set of significant GEI QTL identified by this study not only included QTL identified in either of the conditions independently but also included several that were not identified in independent mapping (Table 2).

### Epistatic interactions contributing to yeast growth plasticity

We computed the broad-sense heritability for each phenotype and found that, in many cases, this exceeded the component of phenotypic variance attributable to individual QTL (Table S2 and Table S3). Hence, the independent effect of QTL may not fully explain the heritable phenotypic variance observed in the segregants. Furthermore, large-effect QTL may have their effect mediated through interactions with QTL of small individual effect. To investigate this possibility, we performed a two-QTL interaction analysis to identify interacting alleles contributing to growth variation. Previously, it has been shown that if neither of the parental strains shows an independent QTL effect, then two-QTL interactions in their segregants are rare (Bloom *et al.* 2013). Due to multiple testing for large number of markers in the whole genome, large sample sizes are needed for adequate power of identifying two-QTL interactions. To alleviate this problem in our relatively small sample size, a subset of markers, selected as described below, was analyzed for potential two-QTL interactions. A caveat of using a subset for epistasis mapping was that it is possible that an interaction may not be finely mapped because the marker could be in partial linkage with the causal locus.

**Figure 3:**
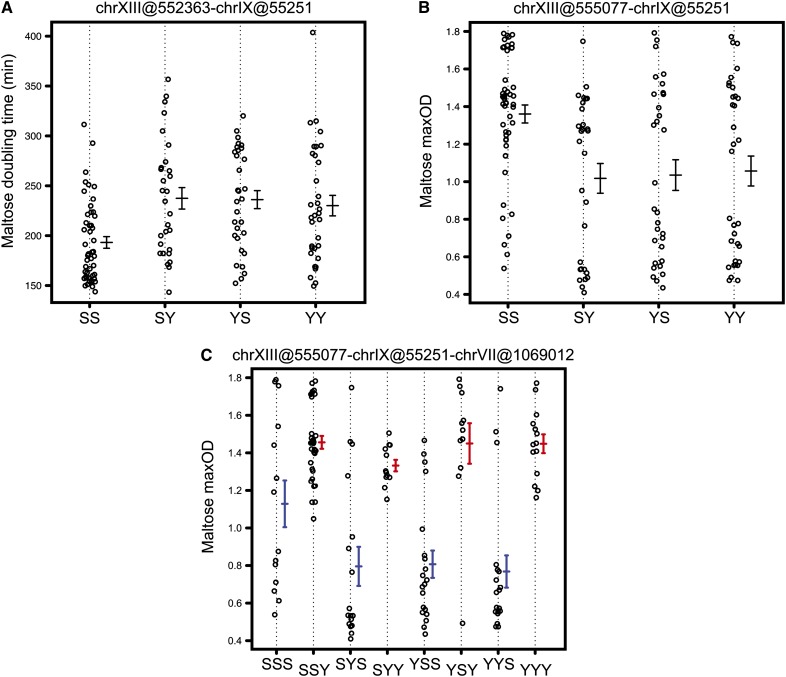
Scatter plots for growth parameters in sucrose showing two-QTL and three-QTL interactions. (A, B) Two-QTL scatter plots for chrV(371,899) and chrXV(473,018) for doubling time and maxOD, respectively. The QTL are indicated as chromosome number followed by marker position in bp within brackets. The x-axis indicates biallelic marker genotype in the QTL order written above the plots. (C) A three-QTL scatter plot for chrV(525,070), chrXV(473,018), and chrV(371,899) for maxOD. The QTL are indicated as chromosome number followed by marker position in bp within brackets. The x-axis indicates triallelic marker genotype in the QTL order written above the plot. The color of error bars (± 1 SE) indicates allele of chrV(525,070) marker with S allele (blue) and Y allele (red).

To increase our initial sets of loci for the interaction scan and thereby increase our sensitivity in detecting interactions, the LOD score cutoff was lowered. Hence, we considered as candidate locus for the interaction scan any locus with a LOD score larger than the smaller maximum genome-wide LOD score obtained in the 1000 random permutations. We also considered candidate loci with evidence for a GEI QTL (*P* < 0.5 across 100 permutations). This analysis showed that QTL of weak independent effect (*i.e.*, those that do not pass a stringent genome-wide significance threshold) combined with GEI QTL can be involved in statistically significant two-QTL interactions (Table 1, Figure 3, A and B, Figure S3). For QTL involved in the same pathway, the variation in phenotype due to a polymorphism at one locus depends on the phenotypic direction of the allele at the second locus. The probability of this will depend on the effect size of the second locus. To identify such epistatic interactions, we conducted a targeted scan where we identified interactions among a large-effect QTL and small-effect single or GEI QTL. We chose the following large-effect QTL from both the parameters: chrII(558,465), chrVII(1,069,000), and chrXIV(467,221) from doubling time and chrV(371,899), chrV(525,070), and chrVII(1,069,012) from maxOD (Table 1 and File S2). In addition to previously mapped two-QTL interactions, this approach identified novel interactions (Table 1).

Analogous to the results of QTL mapping, no overlap was observed in two-QTL interactions across growth parameters except for two interactions, namely, chrI(33,865)-chrV(377,186) in glucose and chrV(371,899)-chrXV(473,018) in sucrose, which were identified for both doubling time and maxOD (Table 1). Thus, most of the potential epistatic interactions were both growth parameter–specific and environment-specific.

However, it is very likely that a gross phenotype like growth is regulated by interactions among more than two QTL. It is challenging, both experimentally and computationally, to identify such genetic interactions. A targeted three-QTL analysis was performed where we searched for interactions between large-effect QTL (see above) and QTL involved in two-QTL interactions (Table 1).

## Discussion

Although rich conditions result in high growth, mild stress results in increased survival (Lin *et al.* 2002). Depending on the evolutionary pressures in the environment, either high growth (in conditions of competition between species and strains) or longer survival (in scarce conditions) could contribute to fitness. In growth itself, trade-offs between growth rate and growth efficiency have been extensively discussed (Maclean 2008). Hence, environment-dependent growth plasticity, especially for a sessile organism like yeast, would aid in maintenance of optimum fitness. For yeast, the availability and utilization of carbon sources affect every aspect of growth (Broach 2012), and many genes are known to respond to a change in the type and level of carbon sources (Daran-Lapujade *et al.* 2004; Wu *et al.* 2004). Attempts have been made to identify loci contributing to variation in yeast growth under different environmental conditions (Cubillos *et al.* 2011; Bloom *et al.* 2013). However, the mechanisms by which genetic interactions affecting different aspects of yeast growth are modulated by the nature of available carbon sources in a growth medium are not well-understood.

In the present study, we mapped variation in phenotypic plasticity across various carbon sources for a large number of segregants of yeast, and performed a genome-wide single-environment QTL analysis as well as focused multi-QTL interaction analyses for doubling time and maxOD (Table 1). A number of loci associated with carbon-source–dependent phenotypic plasticity for the two growth parameters were mapped (Table 2). This plasticity variation was attributable to different sets of GEI QTL for growth rate and for biomass. Using our targeted approach, multi-QTL interactions involving known and novel loci contributing to variation in growth were identified.

We identified large-effect QTL independently in each environment for both of the growth parameters. GEI mapping was performed to identify small-effect QTL that contributed to growth plasticity across pairs of environments. This mapping identified alleles that acted antagonistically across pairs of environments. Such crossover interactions might occur when one allele is sensitive to an environmental variable and the other shows a resilient phenotype in the same environment. Alternatively, the parental strains might be oppositely adapted to different environmental conditions at these loci. For example, glycerol and ethanol, both non-fermentable carbon sources, have a low correlation for maxOD (0.11) compared to doubling time (0.58) (Table S1). Although no GEI QTL were identified between them for doubling time, three crossover GEI QTL were identified between them (Table 2) for maxOD, suggesting that not only different but also antagonistically acting genetic loci regulate growth in them.

In this study, a two-QTL analysis identified epistatic interactions of known QTL contributing to variation as well as novel epistatic interactions among QTL, which did not have an independent effect as single-environment QTL. Similarly, for many conditions in which single-environment QTL mapping did not identify any loci, epistatic interactions between QTL were observed, suggesting that environment-specific epistatic interactions contribute to differential growth of a population in different media. We identified an interaction between Y allele of chrV(371,899) and S allele of chrXV(473,018), which resulted in increased doubling time and decreased maxOD in sucrose (Figure 3, A and B). A three-QTL interaction analysis among these two markers and an additional marker chrV(525,070) for sucrose maxOD suggested that such a two-QTL interaction exists only in presence of Y allele of chrV(525,070) (Figure 3C). However, such a three-QTL interaction was not significant for doubling time. These results suggested that varying degrees of genetic interactions regulate yeast growth plasticity.

**Figure 4:**
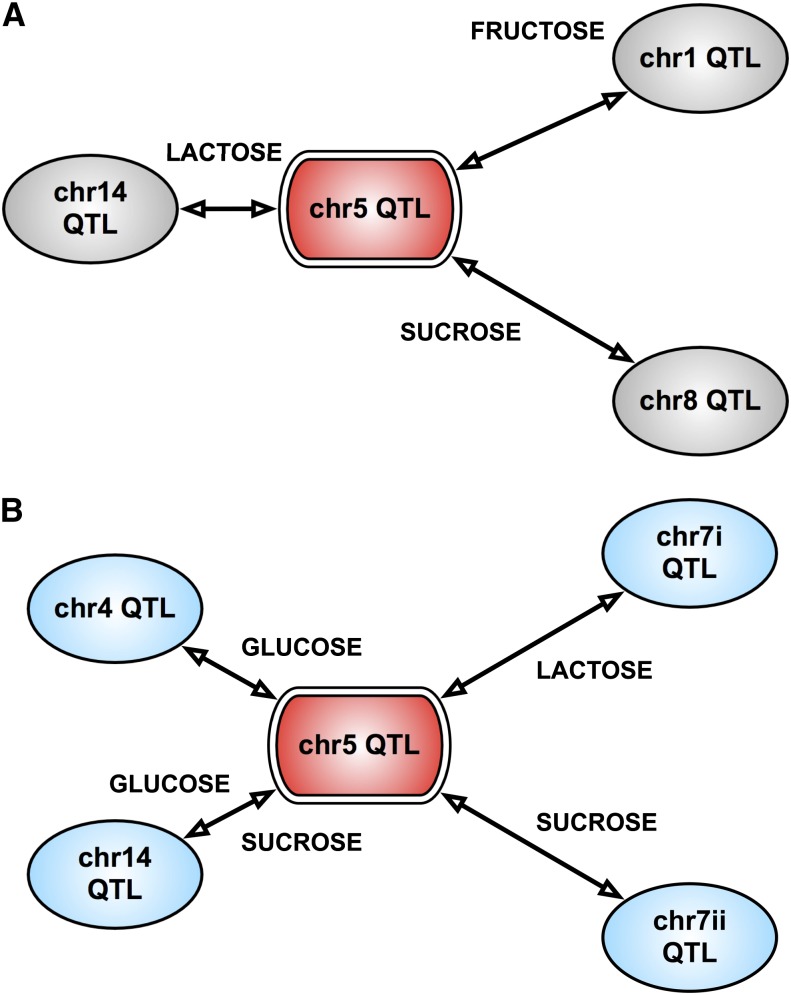
Environment-specific interactions of chrVa [indicated as chrV(371,889) in the main text] QTL. (A, B) Two-QTL and three-QTL interactions, respectively, for doubling time. (C, D) Two-QTL and three-QTL interactions, respectively, for maxOD. Environment is indicated on the interaction line. For QTL on the same chromosome, two genetically distant markers are indicated as (a) and (b). The marker positions, LOD scores, and *P* values for each interaction are given in Table 1.

The chrV(371,899) QTL, which had an independent effect only in fructose maxOD, modulated growth variation in different environments (fructose, glucose, maltose, sucrose, and glycerol) through growth parameter–specific epistatic interactions (Figure 4). These genetic interactions were identified through a targeted two-QTL (Figure S3) and three-QTL (Figure S4) analysis for both the growth parameters. This suggested that such mechanisms exist by which some loci [*e.g.*, chrV(371,899)] regulate growth plasticity in different environments through varied sets of genetic interactions.

Yeast growth is a complex phenotype, with multiple QTL contributing to the various growth parameters (biomass and growth rate) across different carbon sources. The yeast strains in this study demonstrate phenotypic plasticity across these carbon sources, as well as a large variation in this phenotypic plasticity. The presence of QTL–environment interactions and environment-specific multi-QTL interactions contribute to this variation in plasticity. Furthermore, these interactions are parameter-specific, suggesting that yeast has the ability to modulate different aspects of growth independently to maximize its fitness across varied environments. Such targeted studies of genetic interactions can help uncover the genetic factors driving the environmental regulation of complex traits.

## Supplementary Material

Supporting Information
