# Supplementary material for: Yeast Growth Plasticity Is Regulated by Environment-Specific Multi-QTL Interactions
Source: G3 (Bethesda). 2014 Jan 28;4(5):769–77. doi: 10.1534/g3.113.009142 (PMC4025475; doi:10.1534/g3.113.009142)
Supplement: Supporting Information [file supp_4_5_769__index.html]

Yeast Growth Plasticity Is Regulated by Environment-Specific Multi-QTL Interactions — Supporting Information 

# Yeast Growth Plasticity Is Regulated by Environment-Specific Multi-QTL Interactions

## Supporting Information for Bhatia *et al.*, 2014

**Files in this Data Supplement:**

- Supporting Information - Figures S1-S4, Tables S1-S3, and Files S1-S2 (PDF, 140 KB)
- Figure S1 - Scatter plots of QTL identified in various environment conditions. (PDF, 35 KB)
- Figure S2 - Reaction norms of various GEI QTL identified across pairs of various environment conditions. (PDF, 359 KB)
- Figure S3 - Scatter plots of two-QTL interactions identified in various environment conditions. (PDF, 48 KB)
- Table S1 - a. Pearson Correlation Coefficient between various growth media for doubling time (n = 144 segregants). b. Pearson Correlation Coefficient between various growth media for maxOD (n = 144 segregants). (PDF, 65 KB)
- Table S2 - Broad sense heritabilities for each growth parameter and environmental condition (parentheses indicate 95% CI). (PDF, 65 KB)
- Table S3 - Total percentage of phenotypic variance attributable to single-environment QTL. (PDF, 71 KB)
- File S1 - Additional methods for single environment, GEI and two-QTL mapping. (PDF, 128 KB)
- Figure S4 - Scatter plots of three-QTL interactions identified in various environment conditions. (PDF, 24 KB)
- File S2 - R scripts, custom Python code and data files for single environment, GEI and multi-QTL mapping. Also available for download from http://www.tifr.res.in/~dbs/faculty/hsinha/Bhatia\_et\_al/File\_S2.zip. (.zip, 1 MB)
