# Supplementary material for: Yeast Growth Plasticity Is Regulated by Environment-Specific Multi-QTL Interactions
Source: G3 (Bethesda). 2014 Jan 28;4(5):769–77. doi: 10.1534/g3.113.009142 (PMC4025475; doi:10.1534/g3.113.009142)
Supplement: Supporting Information [file supp_g3.113.009142_009142SI.pdf]

G3 · Genes | Genomes | Genetics

Yeast growth plasticity is regulated by environment specific multi-QTL interactions

Aatish Bhatia,*[,1] Anupama Yadav,[†,1] Chenchen Zhu,[‡] Julien Gagneur,[§] Aparna Radhakrishnan,[†] Lars M. Steinmetz,[‡,**,††] Gyan Bhanot,*[,†,‡‡,§§,***,†††] and Himanshu Sinha[†,2]

*Department of Physics and Astronomy and [‡‡]BioMaPS Institute for Quantitative Biology and [§§]Department of Molecular Biology and Biochemistry, Rutgers University, Piscataway, New Jersey 08854, [†]Department of Biological Sciences, Tata Institute of Fundamental Research, Mumbai 400005, India, [‡]European Molecular Biology Laboratory, Genome Biology Unit, 69117 Heidelberg, Germany, [§]Gene Center, Ludwig-Maximilians-Universität, 81377 Munich, Germany, **Department of Genetics, Stanford University School of Medicine, Stanford, California 94305, [††]Stanford Genome Technology Center, Stanford University, Palo Alto, California 94304, [***]Rutgers Cancer Institute of New Jersey, New Brunswick, New Jersey 08903, [†††]Simons Center for Systems Biology, Institute for Advanced Study, Princeton, New Jersey 08540

[1]These authors contributed equally to this work.

[2]Corresponding author: Department of Biological Sciences, Tata Institute of Fundamental Research, Homi Bhabha Road, Colaba, Mumbai 400005, India. Tel: +91-22-22782827. Email: hsinha@tifr.res.in

DOI: 10.1534/g3.113.009142

Figures S1-S4

Available for download as PDF files at http://www.g3journal.org/lookup/suppl/doi:10.1534/g3.113.009142/-/DC1

Figure S1 Scatter plots of QTL identified in various environment conditions. The QTL are indicated as chromosome number followed by marker position in bp. AA indicates S and AB indicates Y allele. Error bars indicate ±1 S.E.

Figure S2 Reaction norms of various GEI QTL identified across pairs of various environment conditions. The QTL are indicated as chromosome number followed by marker position in bp. Error bars indicate ±1 S.E.

Figure S3 Scatter plots of two-QTL interactions identified in various environment conditions. The QTL are indicated as chromosome number followed by marker position in bp. The x-axis indicates biallelic marker genotype in the QTL order written above the plot. AA AA indicates SS allele, AA AB indicates SY allele, AB AA indicates YS and AB AB indicates YY. Alternate error bars (±1 S.E.) are colored blue and red.

Figure S4 Scatter plots of three-QTL interactions identified in various environment conditions. The QTL are indicated as chromosome number followed by marker position in bp. The x-axis indicates biallelic marker genotype in the QTL order written above the plot. AA AA AA indicates SSS allele, AA AA AB indicates SSY allele, AA AB AA indicates SYS, AA AB AB indicates SYY, AB AA AA indicates YSS, AB AA AB indicates YSY, AB AB AA indicates YYS and AB AB AB indicates YYY. Alternate error bars (±1 S.E.) are colored blue and red.

Table S1

a. Pearson Correlation Coefficient between various growth media for doubling time (n = 144 segregants).

Condition	Ethanol	Fructose	Glucose	Glycerol	Lactose	Maltose	Sucrose
Ethanol	1.00	0.35	0.38	0.58	0.63	0.25	0.33
Fructose		1.00	0.41	0.33	0.52	0.19	0.53
Glucose			1.00	0.46	0.45	0.32	0.44
Glycerol				1.00	0.59	0.34	0.34
Lactose					1.00	0.40	0.39
Maltose						1.00	0.15
Sucrose							1.00

b. Pearson Correlation Coefficient between various growth media for maxOD (n = 144 segregants).

Condition	Ethanol	Fructose	Glucose	Glycerol	Lactose	Maltose	Sucrose
Ethanol	1.00	0.13	0.11	0.11	0.04	0.01	0.22
Fructose		1.00	0.18	0.30	0.27	0.07	0.12
Glucose			1.00	0.14	-0.02	0.10	0.27
Glycerol				1.00	0.49	0.05	0.11
Lactose					1.00	0.03	0.05
Maltose						1.00	0.19
Sucrose							1.00

Broad sense heritability

To calculate broad sense heritability, we used the `lmer` function in the lme4 R package (Bates 2010) to perform an analysis of variance with a random effects model on 2 experimental replicates of segregant data (following procedure described in Poecke *et al.* 2007).

The `confint` function (Bates 2010) was used to compute standard errors of the variance estimates provided by `lmer`, and these errors were then propagated to compute the 95% confidence intervals for the broad sense heritability.

Table S2 Broad sense heritabilities for each growth parameter and environmental condition (parentheses indicate 95% CI).

Condition	Ethanol	Fructose	Glucose	Glycerol	Lactose	Maltose	Sucrose
Doubling time	0.13 (0.07, 0.18)	0.44 (0.32, 0.55)	0.65 (0.59, 0.72)	0.96 (0.95, 0.98)	0.40 (0.29, 0.52)	0.53 (0.46, 0.61)	0.20 (0.03, 0.36)
maxOD	0.04 (0.01, 0.09)	0.41 (0.30, 0.53)	0.15 (0.09, 0.21)	0.36 (0.25, 0.47)	0.14 (0.00, 0.32)	0.70 (0.63, 0.77)	0.72 (0.62, 0.82)

References

Van Poecke, Remco MP, et al. "Natural variation in RPS2-mediated resistance among Arabidopsis accessions: correlation between gene expression profiles and phenotypic responses." The Plant Cell Online 19.12 (2007): 4046-4060.

Bates, Douglas M. "lme4: Mixed-effects modeling with R." URL http://lme4.r-forge.r-project.org/book (2010).

Broman, Karl W., and Saunak Sen. A guide to QTL mapping with R-qtl. Springer, 2009.

Percentage of phenotypic variance attributable to single environment QTL

We calculated the fraction of phenotypic variance attributable to single environment QTL using the method described in Broman and Sen (2009). The formula for the percent variance explained by a single QTL is $1 - 10^{(\frac{-2 \times LOD}{n})}$ where LOD is the LOD score of the QTL and n is the number of segregants (n = 144).

Table S3 Total percentage of phenotypic variance attributable to single-environment QTL.

Condition	Ethanol	Fructose	Glucose	Glycerol	Lactose	Maltose	Sucrose
Doubling time	26.2	29.4	24.7	0	40.2	43.2	10.6
maxOD	13.7	11.2	0	0	0	57.6	0

Files S1-S2

Available for download at http://www.g3journal.org/lookup/suppl/doi:10.1534/g3.113.009142/-/DC1

File S1 Additional methods for single environment, GEI and two-QTL mapping.

File S2 R scripts, custom Python code and data files for single environment, GEI and multi-QTL mapping. Also available for download from http://www.tifr.res.in/~dbs/faculty/hsinha/Bhatia_et_al/File_S2.zip.
