# Supplementary figures and images for: Yeast Growth Plasticity Is Regulated by Environment-Specific Multi-QTL Interactions
Source: G3 (Bethesda). 2014 Jan 28;4(5):769–77. doi: 10.1534/g3.113.009142 (PMC4025475; doi:10.1534/g3.113.009142)

chr02\_516338

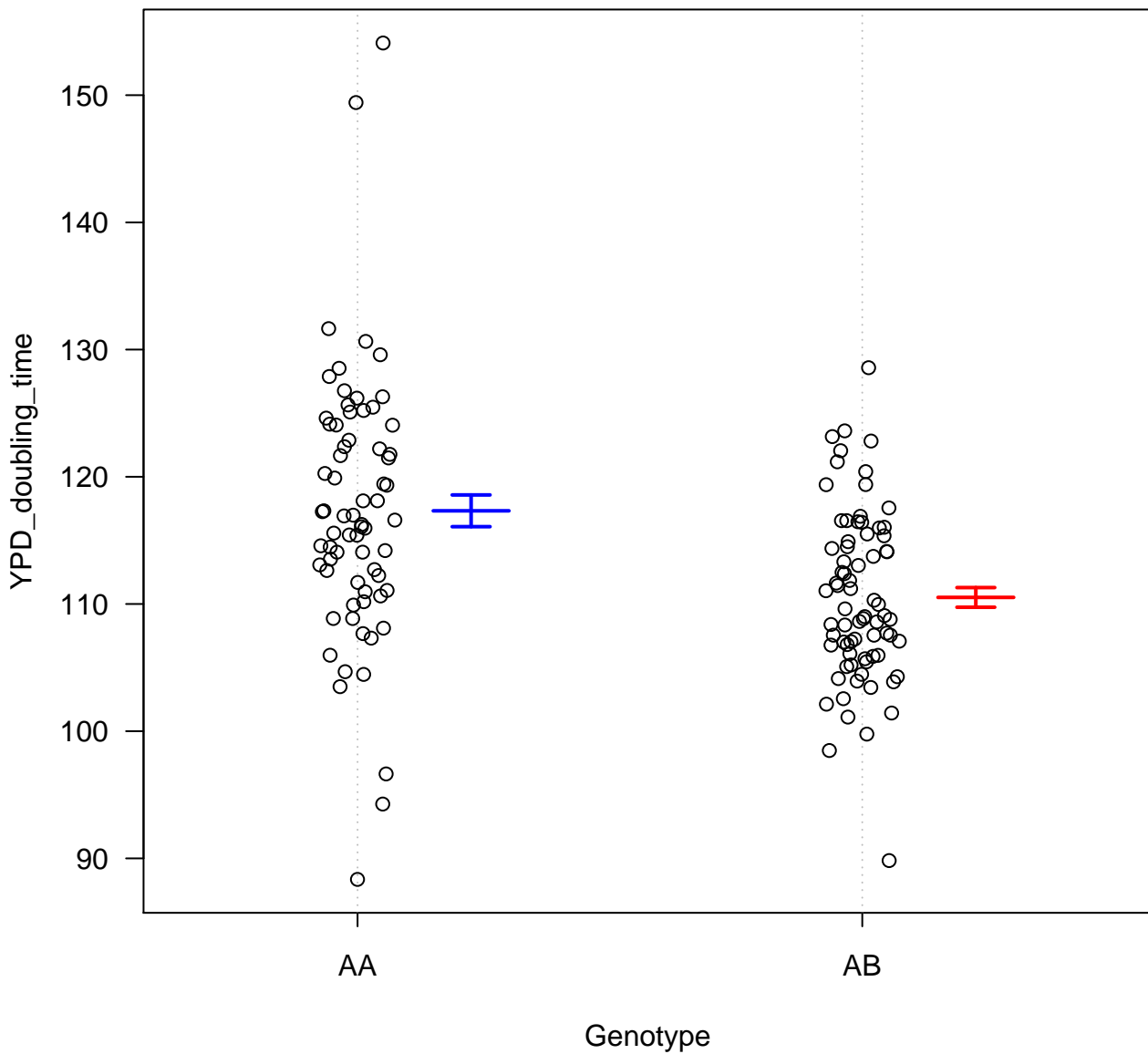

chr14\_491256

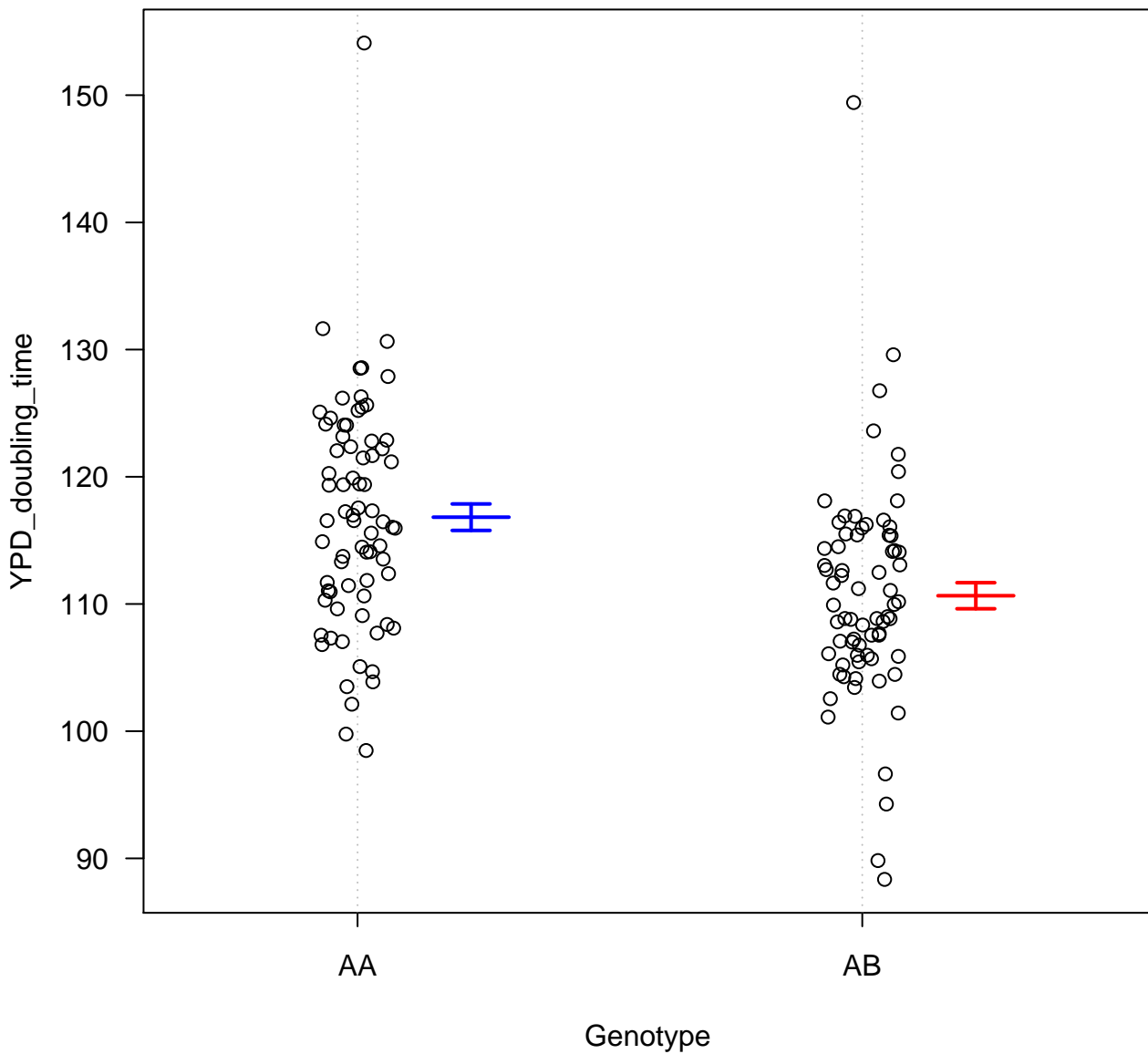

chr14\_465189

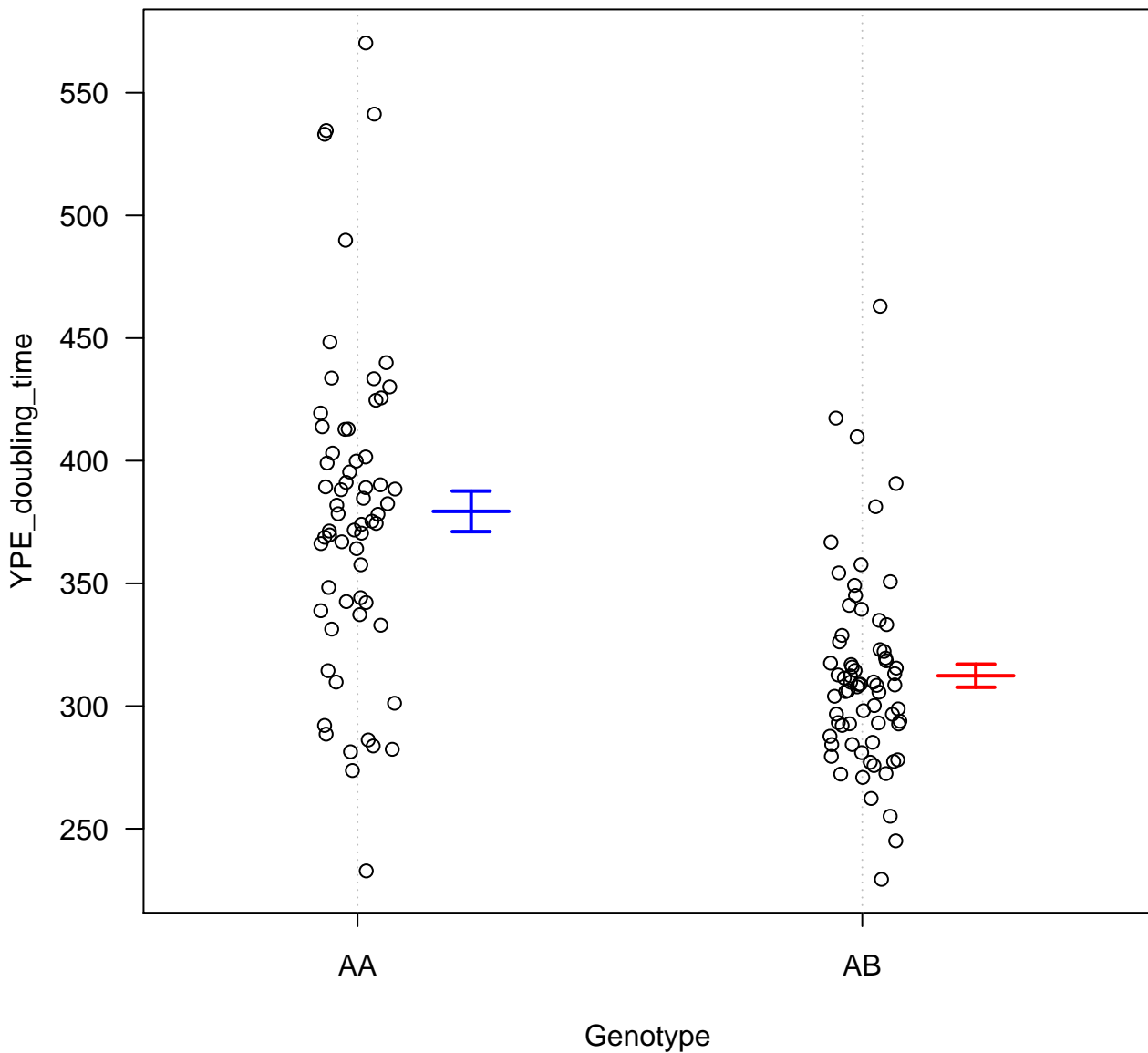

chr05\_525070

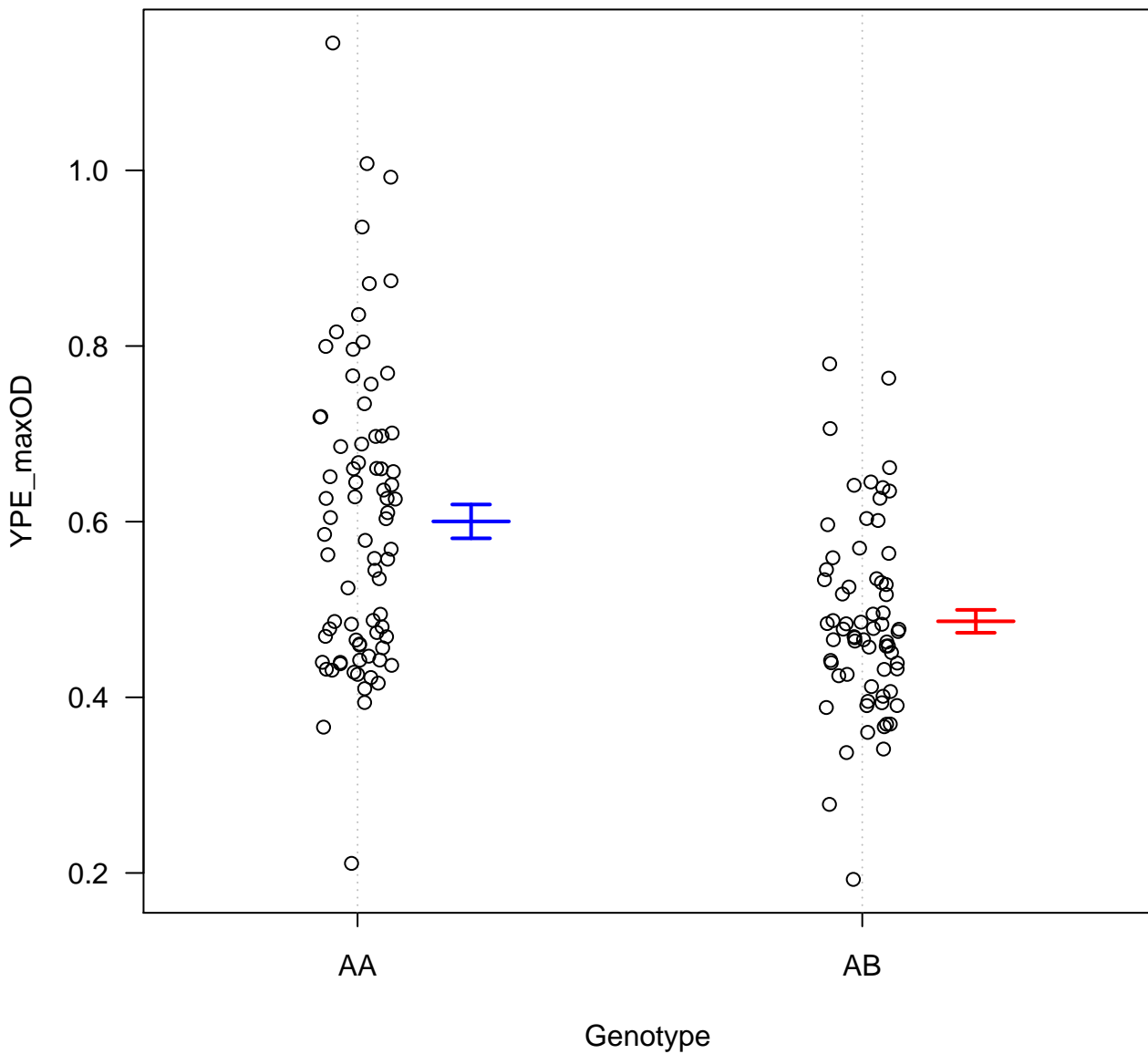

chr02\_558465

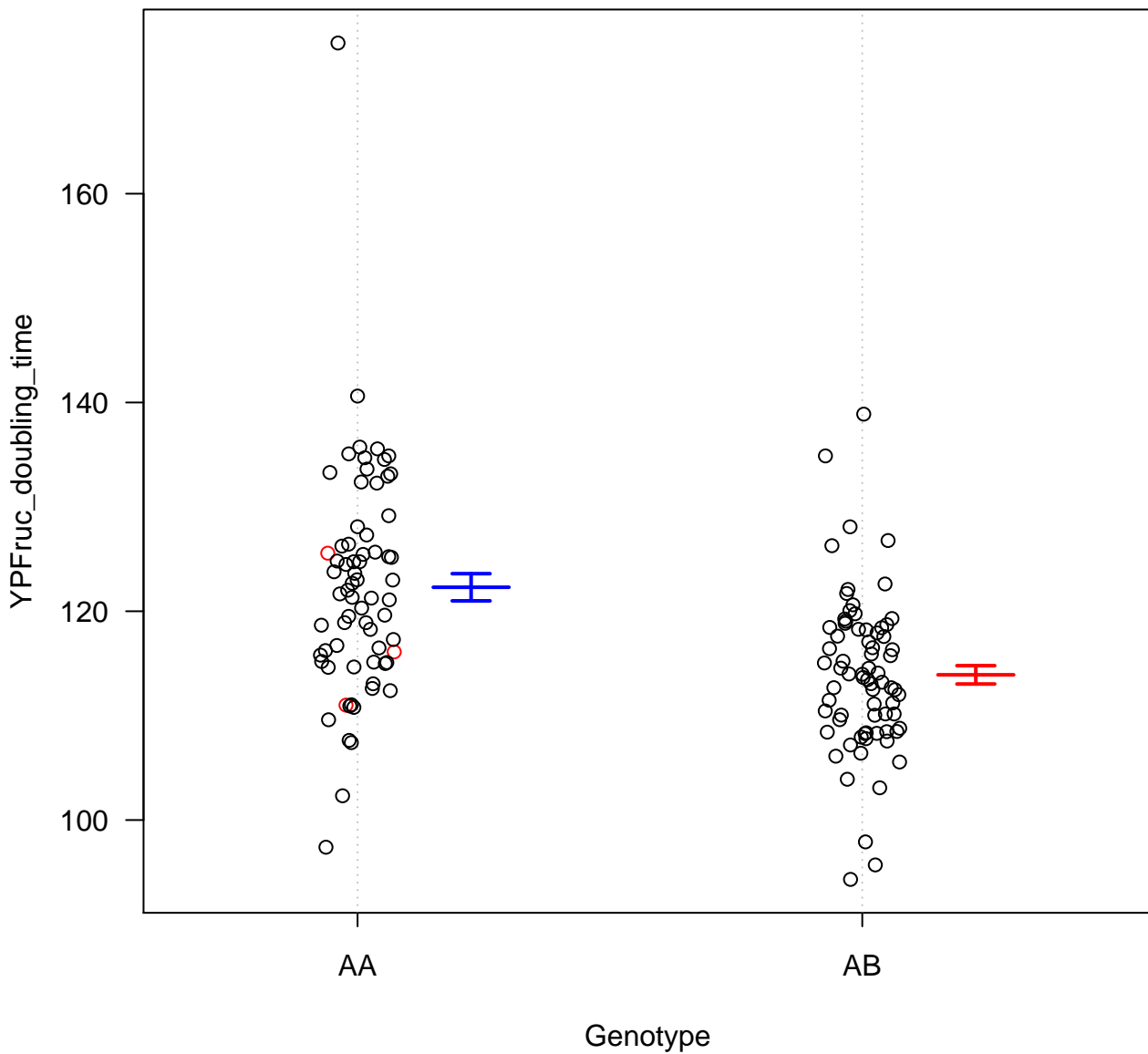

chr13\_26435

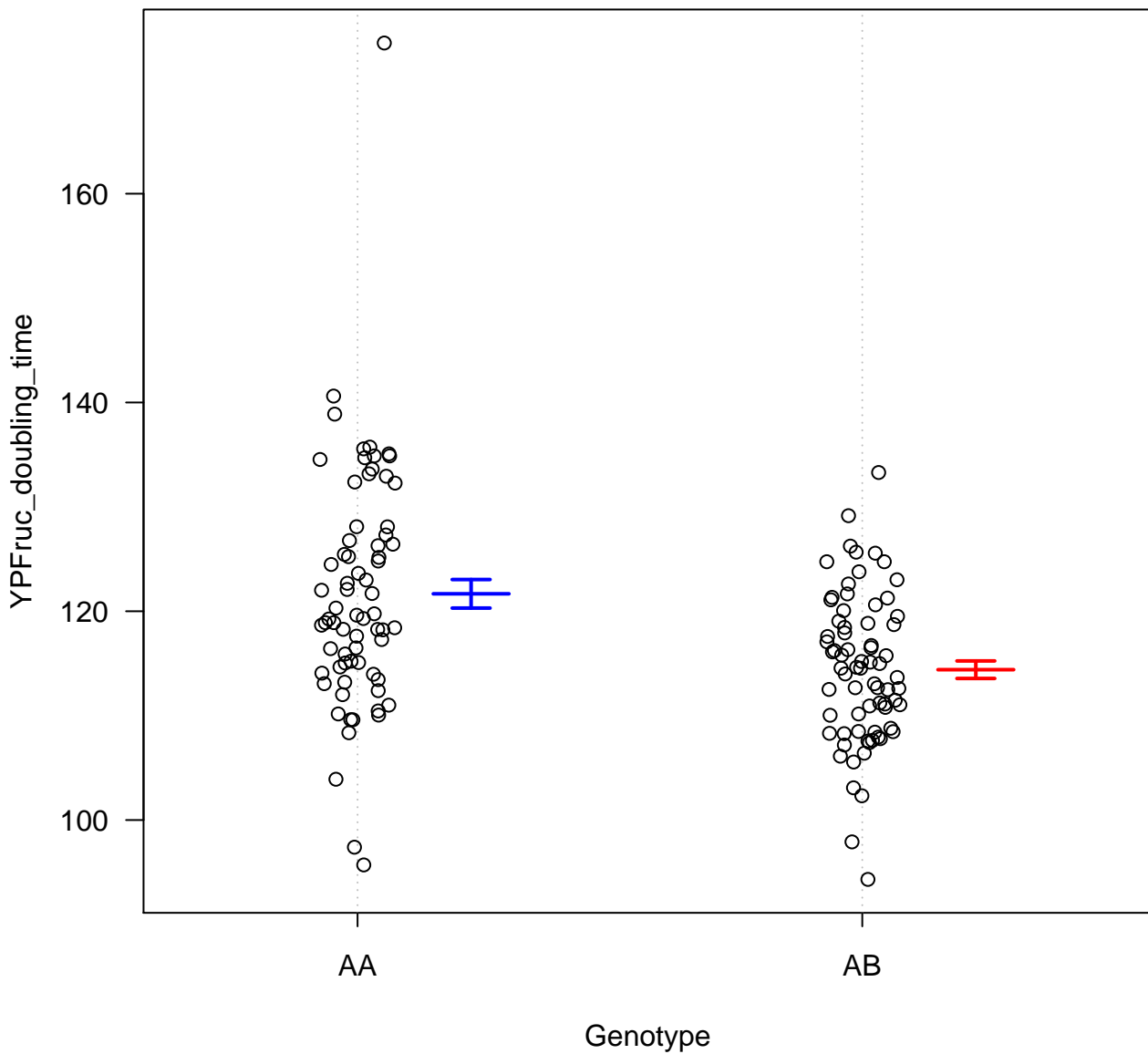

chr05\_371899

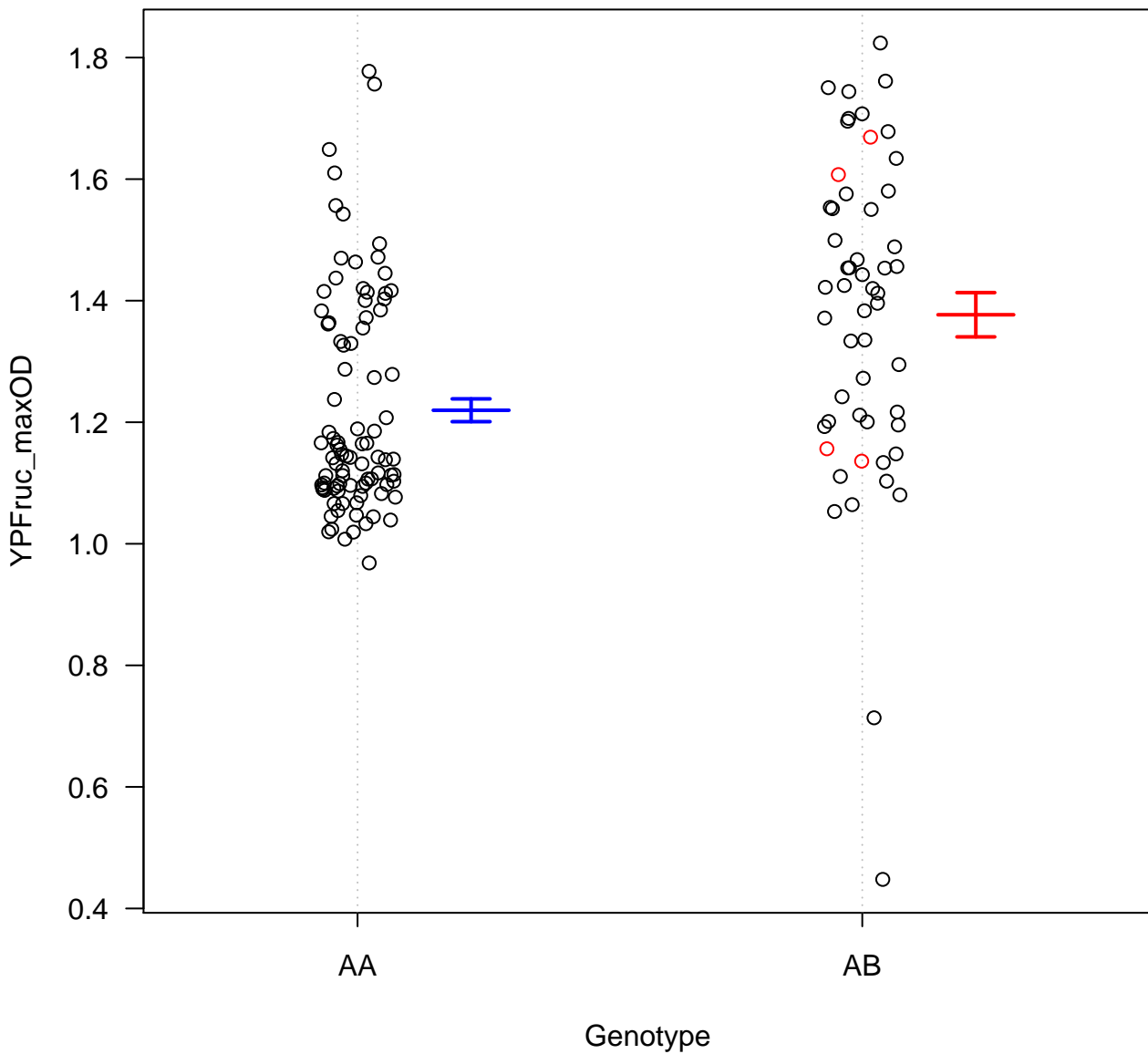

chr14\_441202

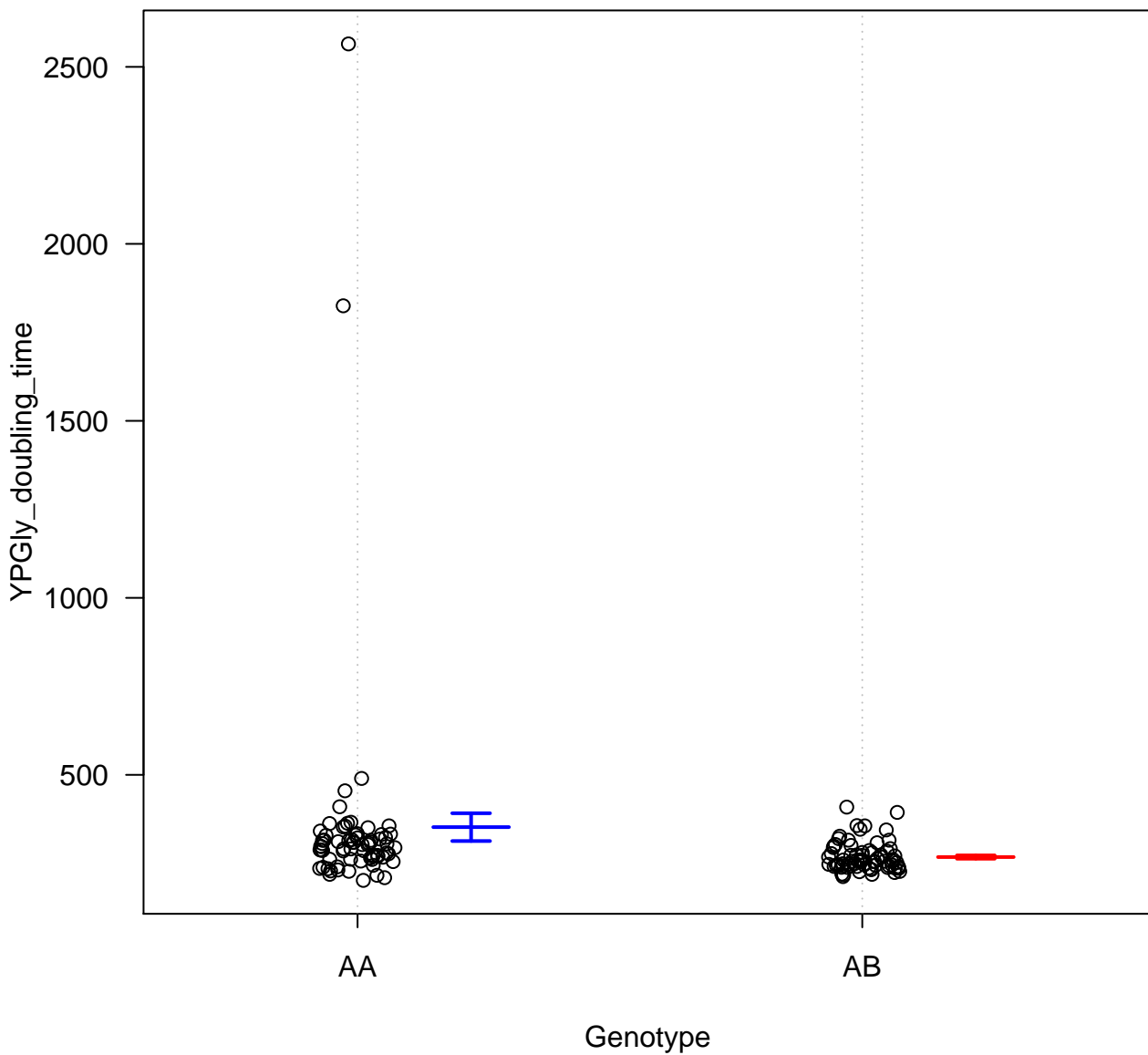

chr14\_467221

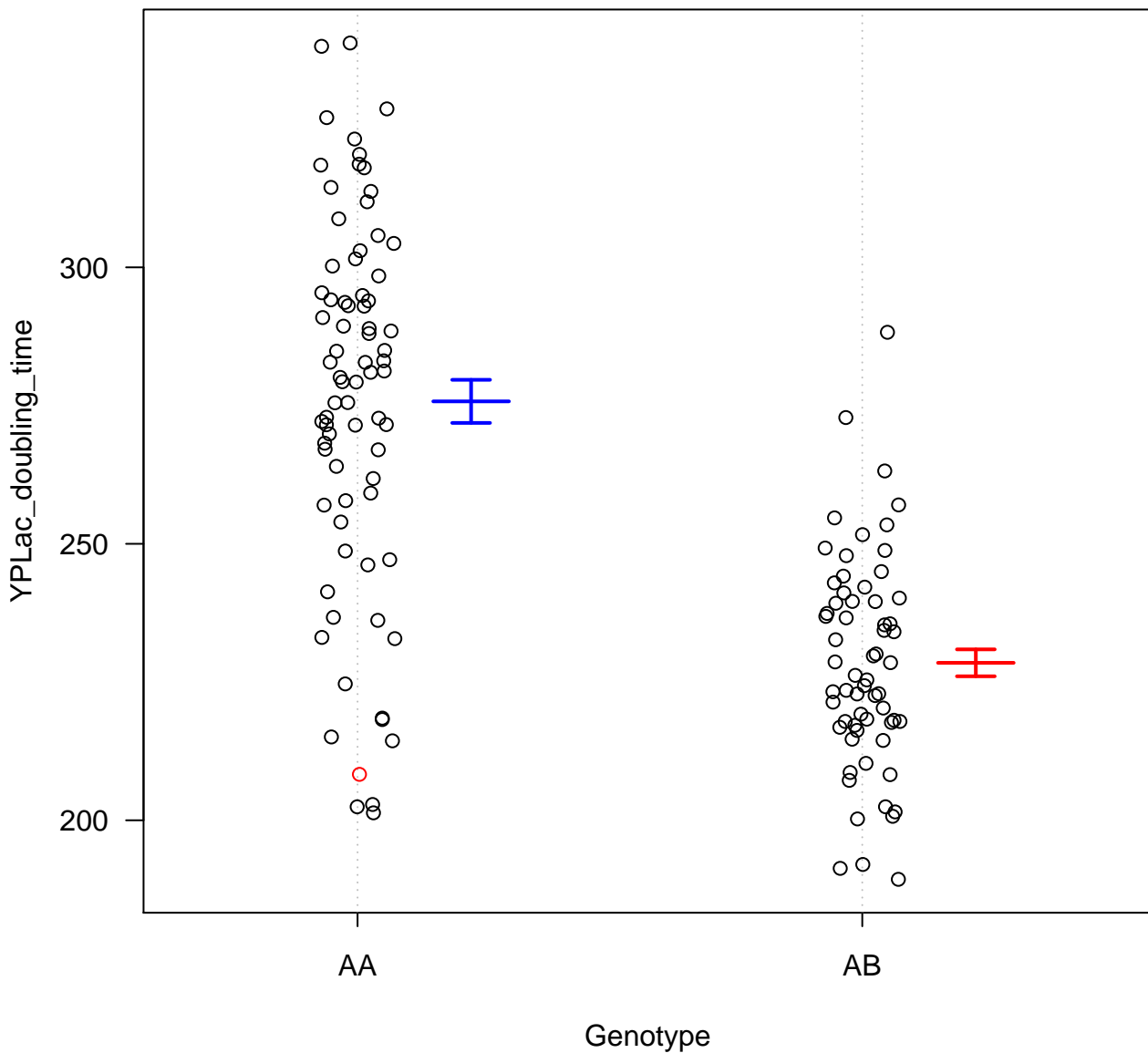

chr03\_56309

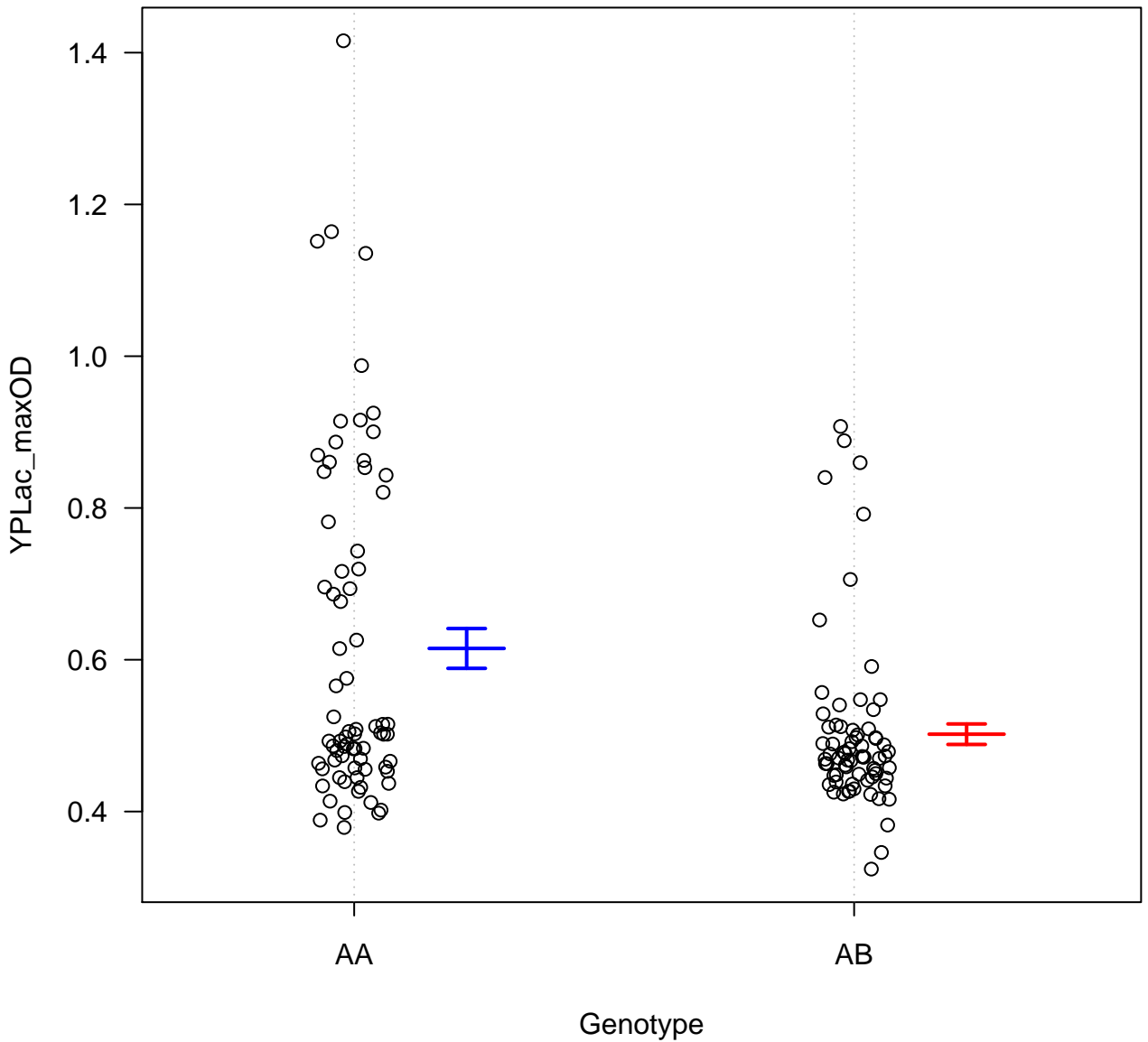

chr07\_1069012

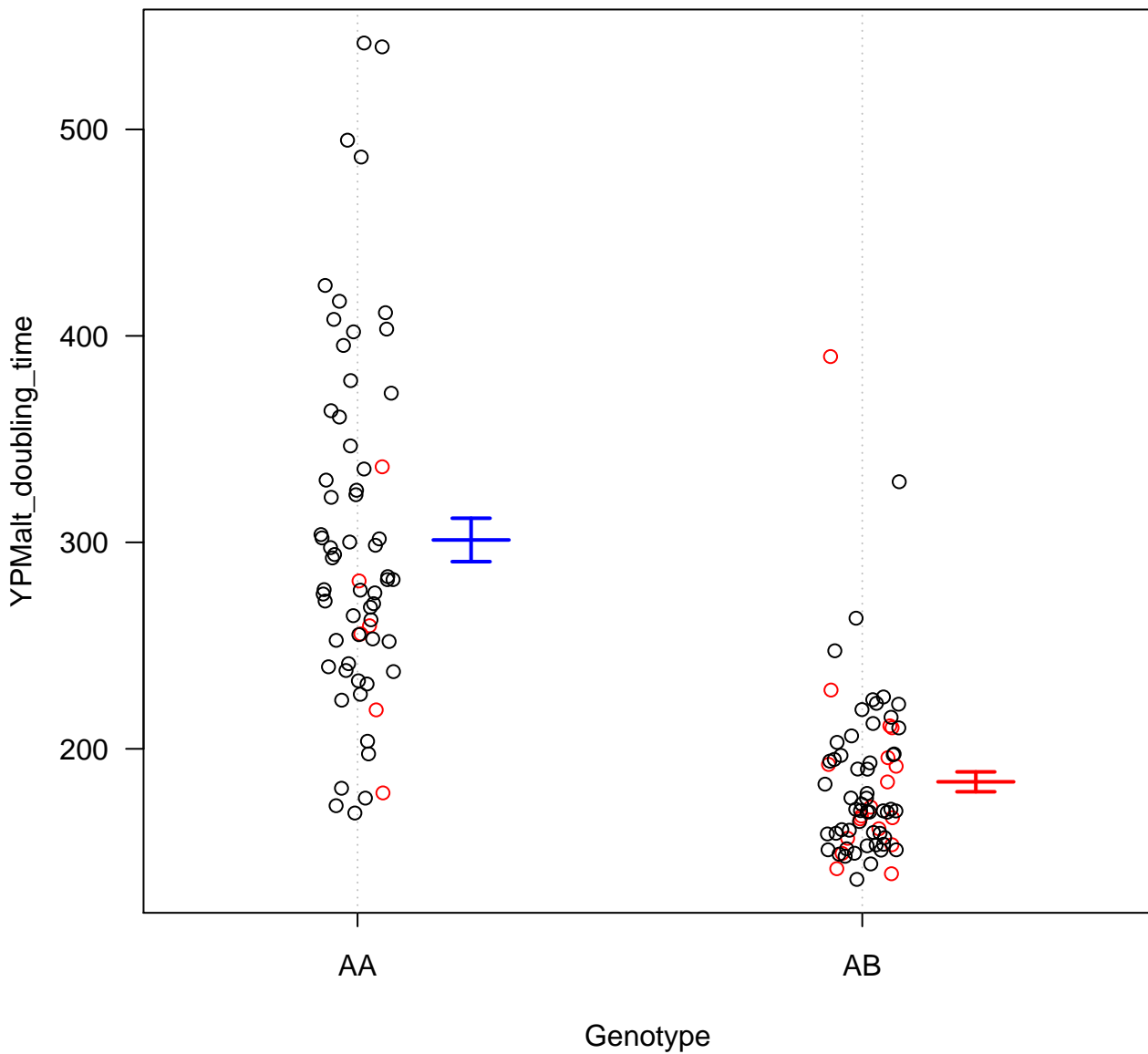

chr15\_656568

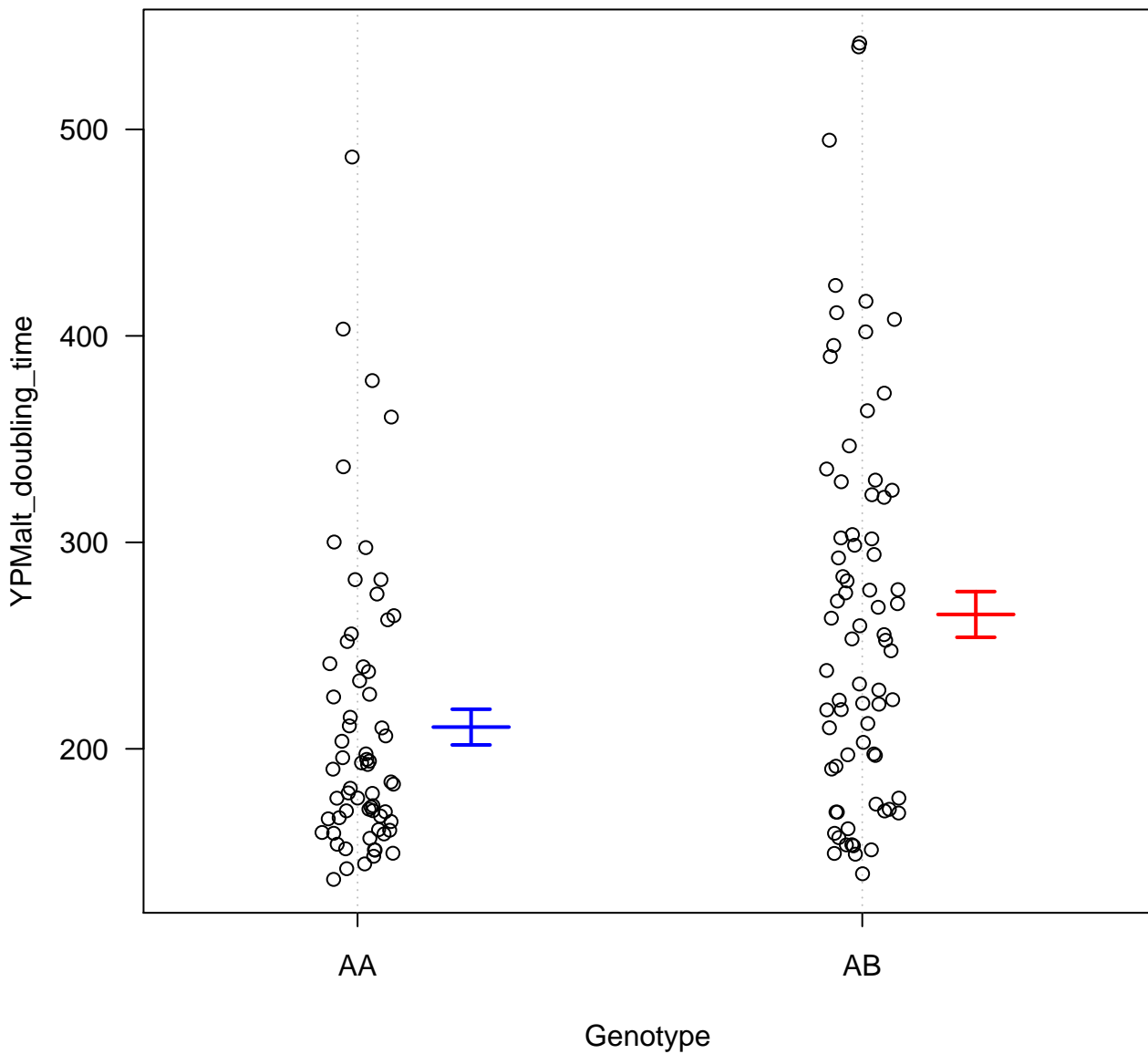

chr07\_1069000

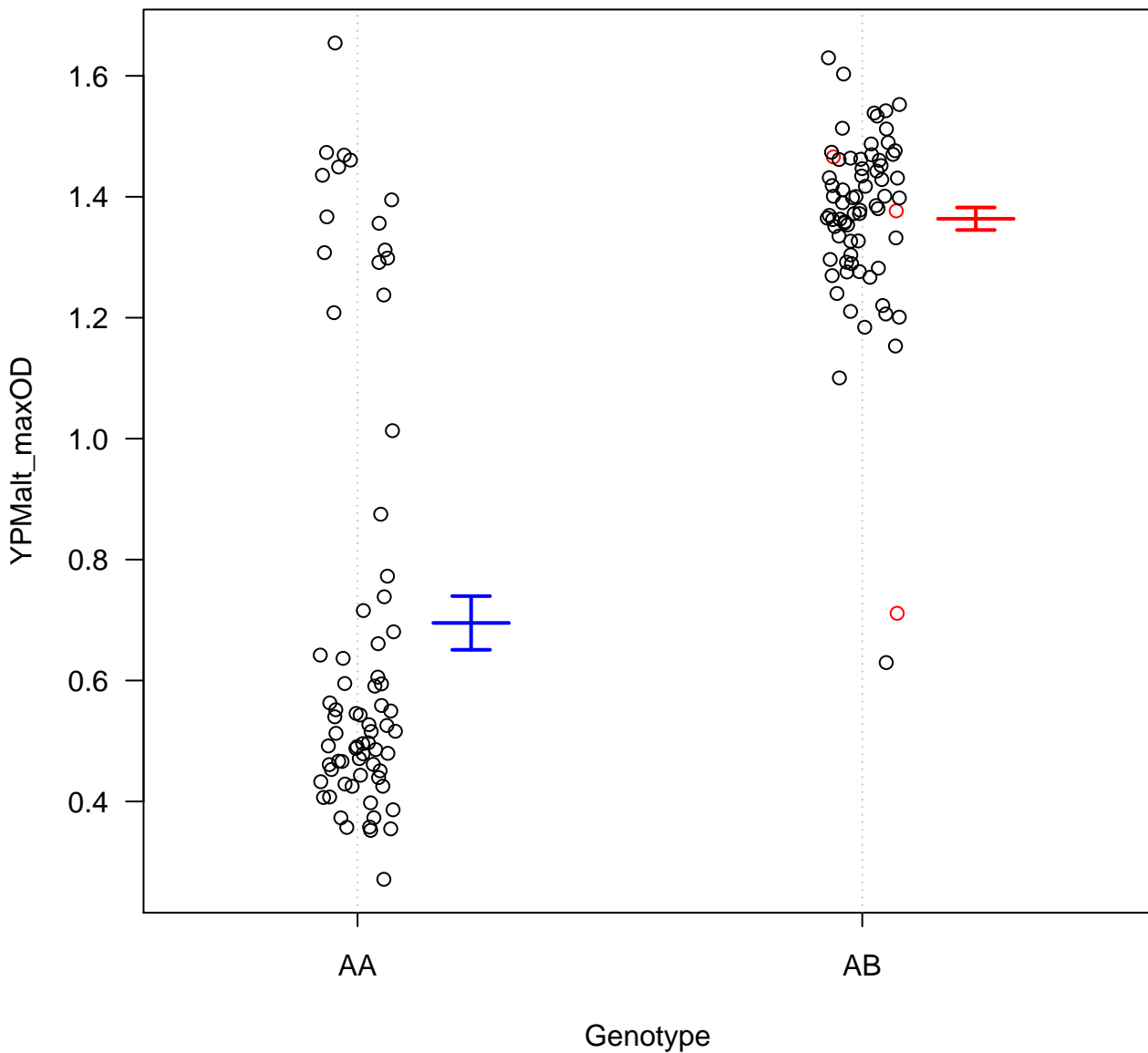

chr02\_516338

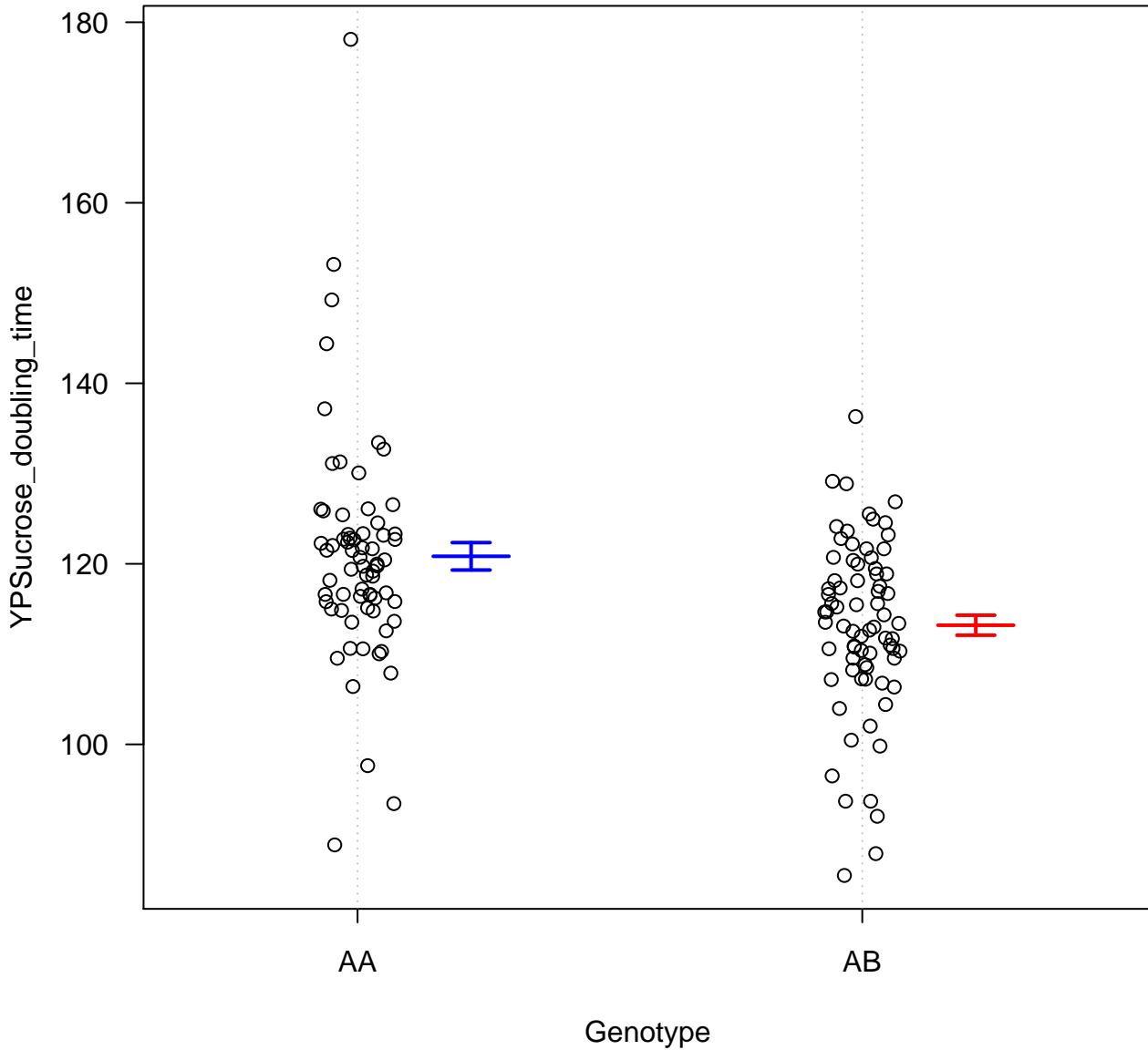

Supplement: Supporting Information [file supp_g3.113.009142_FigureS1.pdf]

chr02\_558465  
chr13\_26435  
chr07\_42563

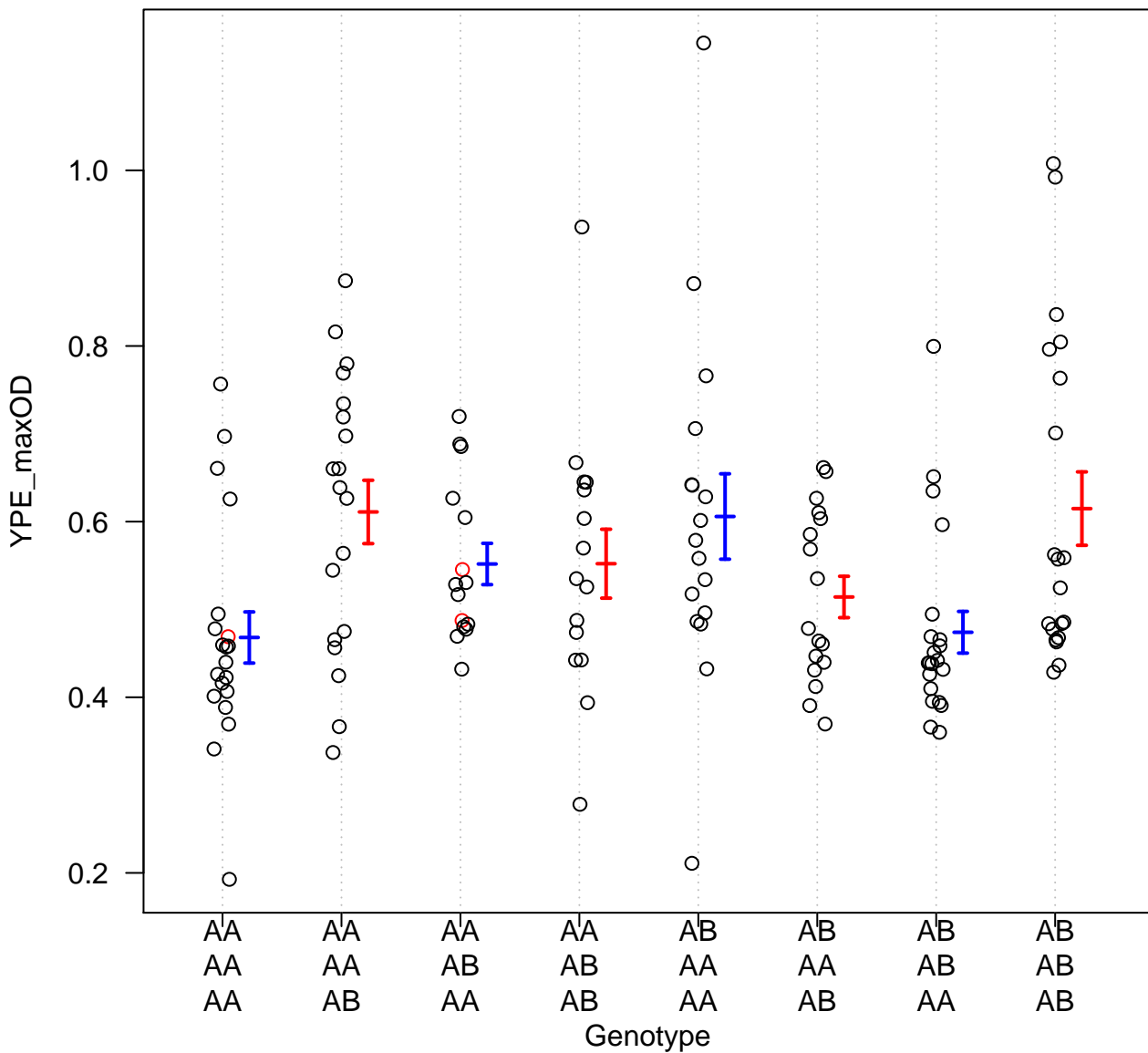

chr05\_371899  
chr13\_555077  
chr02\_182131

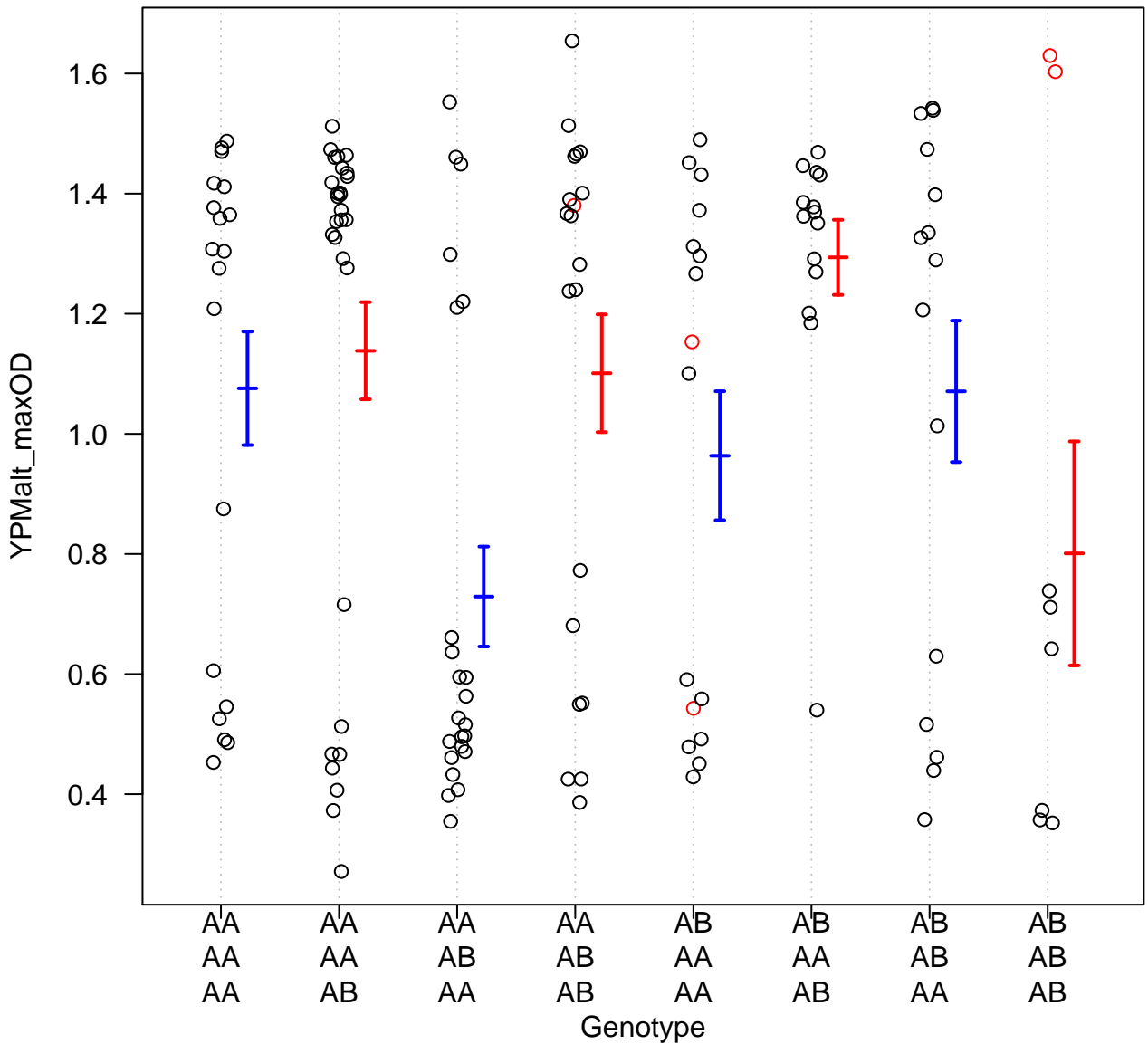

chr02\_558465  
chr08\_145761  
chr05\_377186

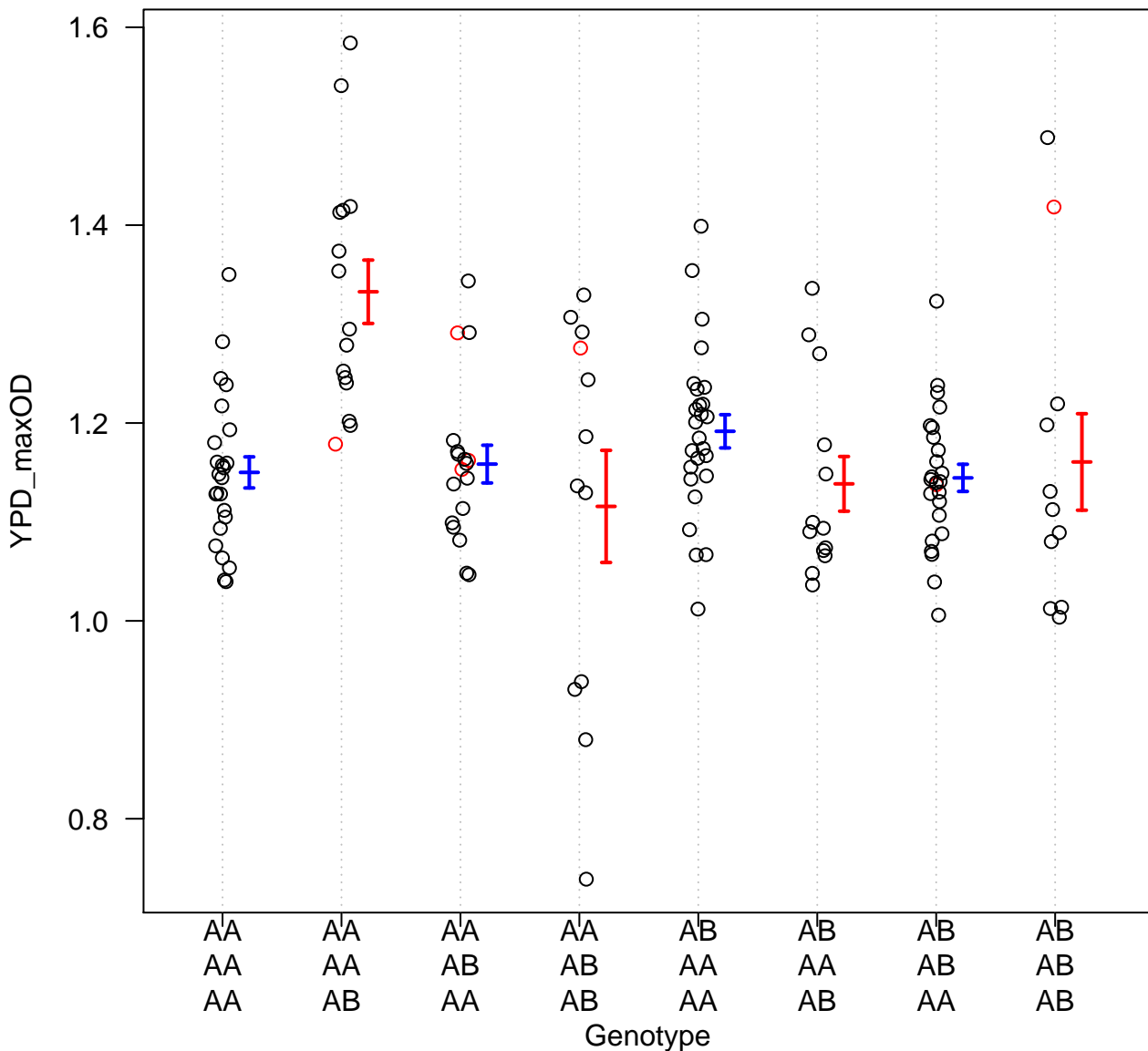

chr05\_525070  
chr15\_473018  
chr05\_371899

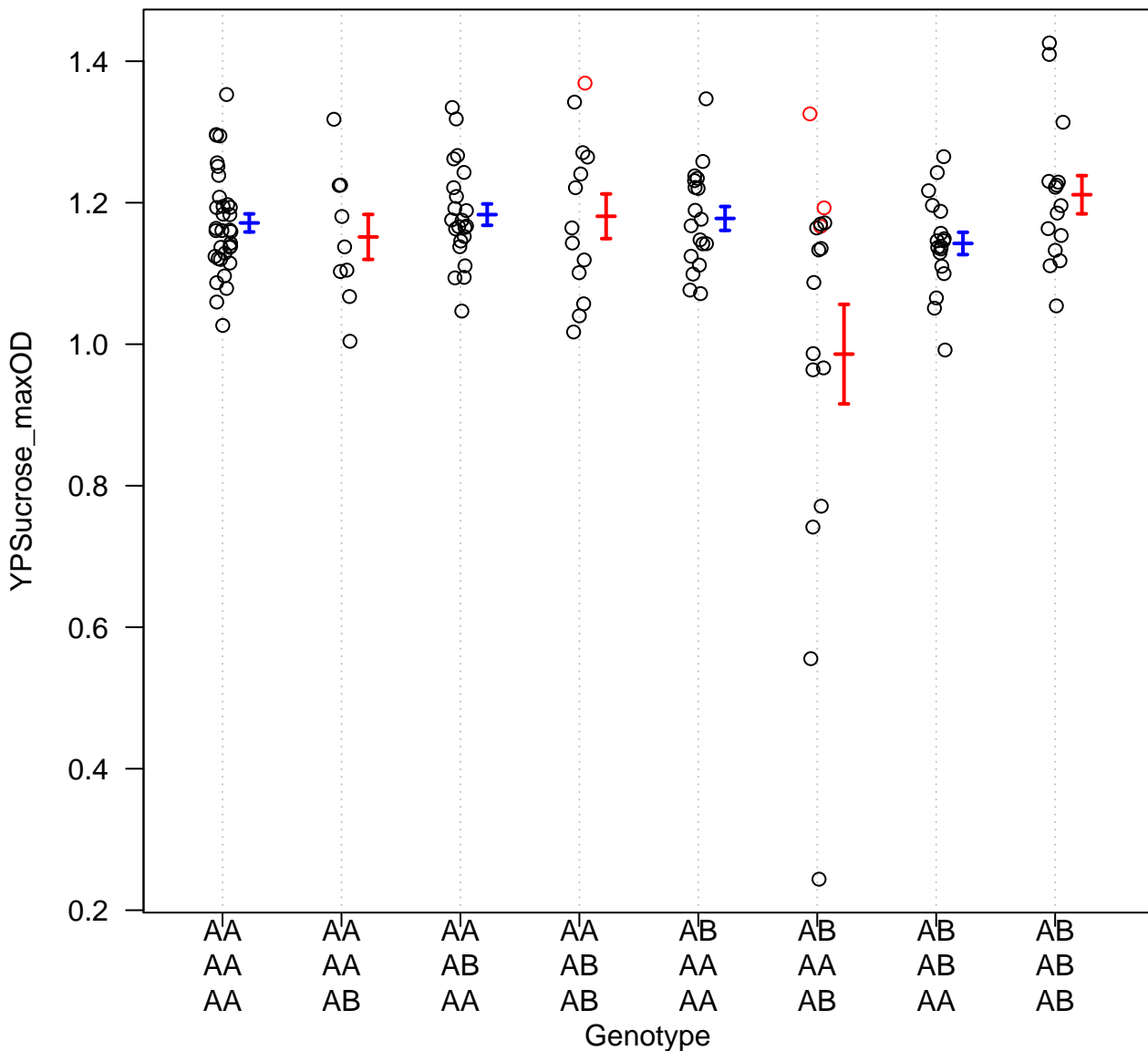

chr05\_525070  
chr05\_371899  
chr01\_62951

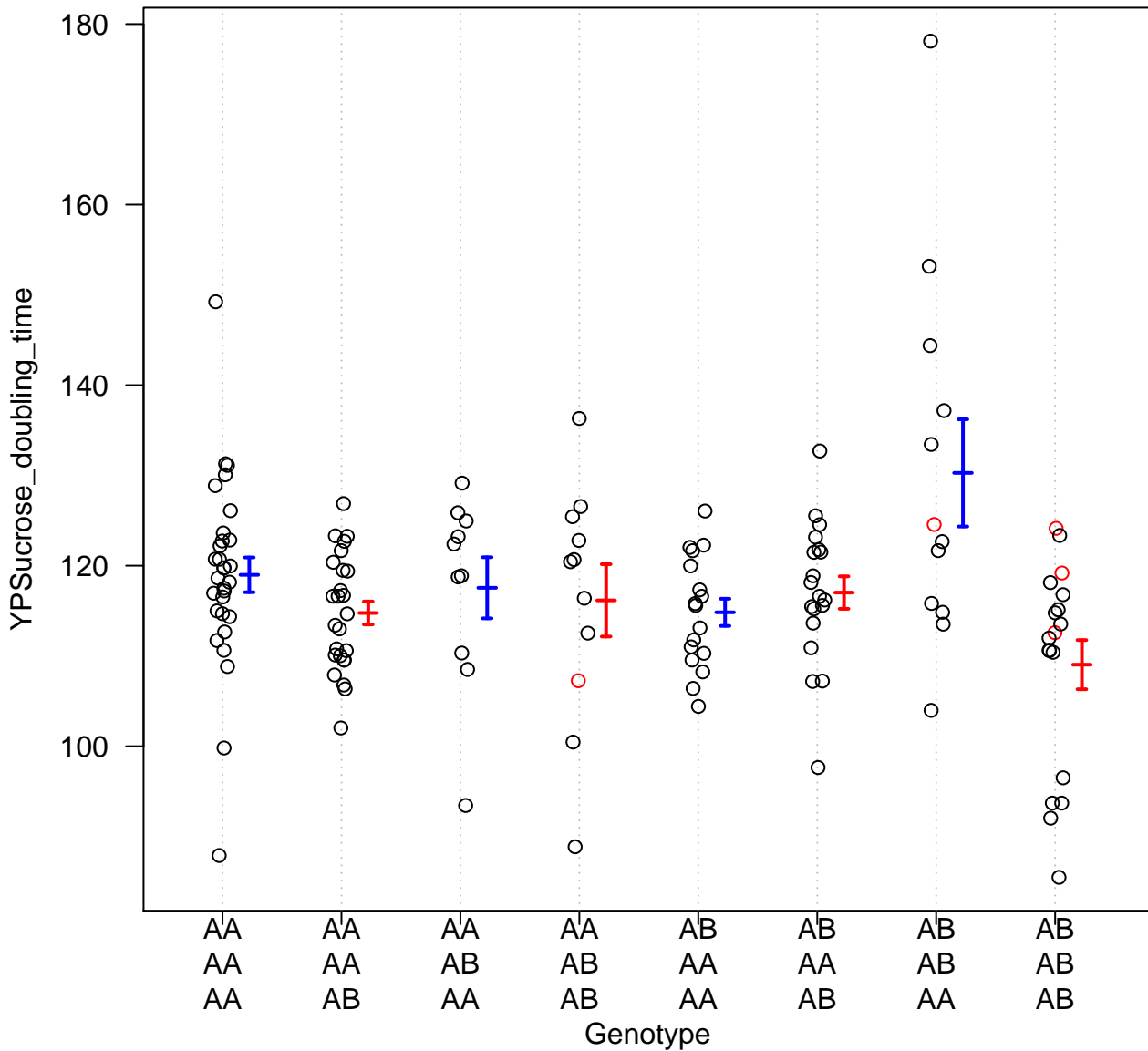

chr05\_525070  
chr05\_371899  
chr02\_516338

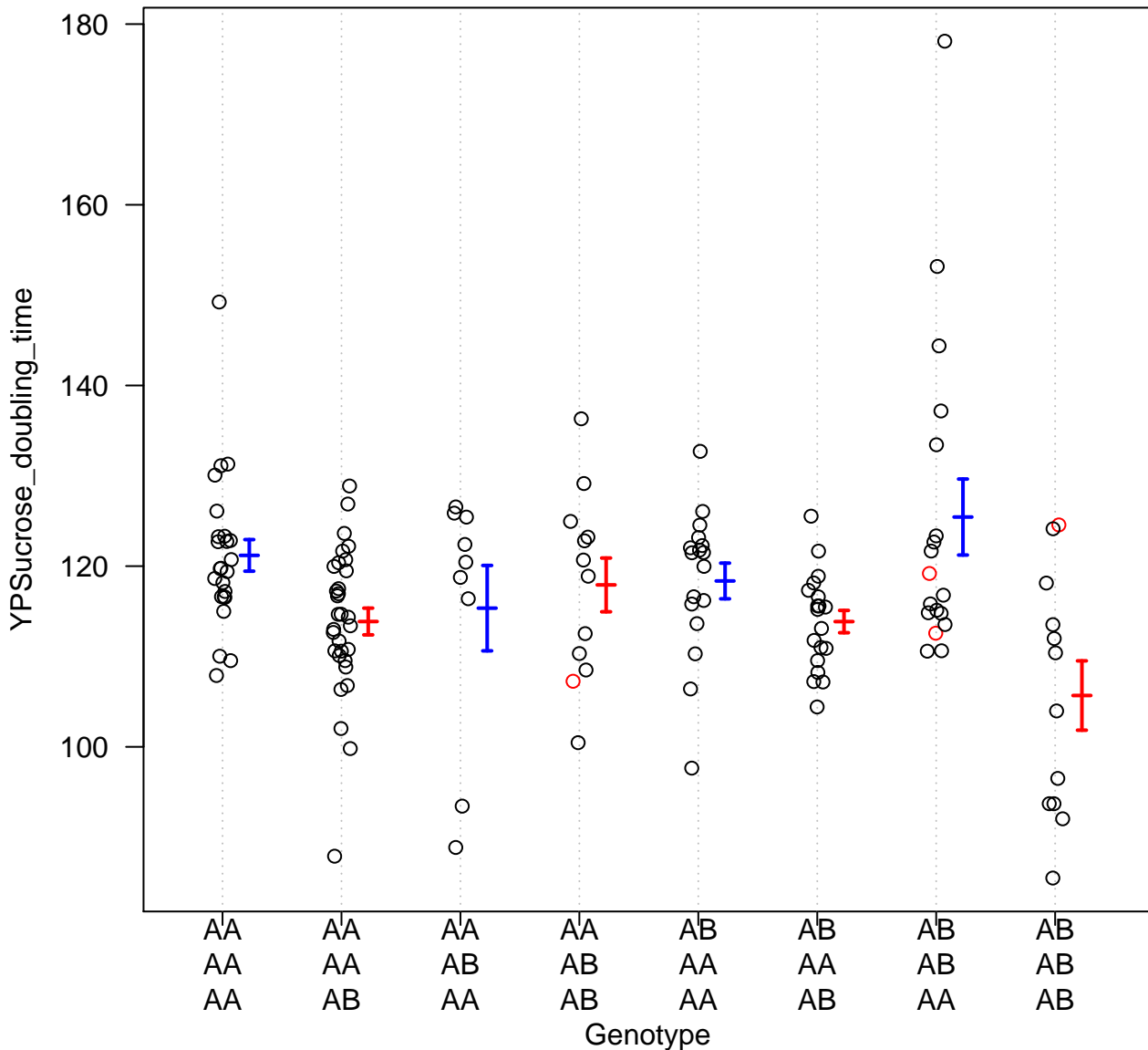

Supplement: Supporting Information [file supp_g3.113.009142_FigureS4.pdf]
