# Supplementary material for: Yeast Growth Plasticity Is Regulated by Environment-Specific Multi-QTL Interactions
Source: G3 (Bethesda). 2014 Jan 28;4(5):769–77. doi: 10.1534/g3.113.009142 (PMC4025475; doi:10.1534/g3.113.009142)
Supplement: Supporting Information [file supp_g3.113.009142_FigureS2.pdf]

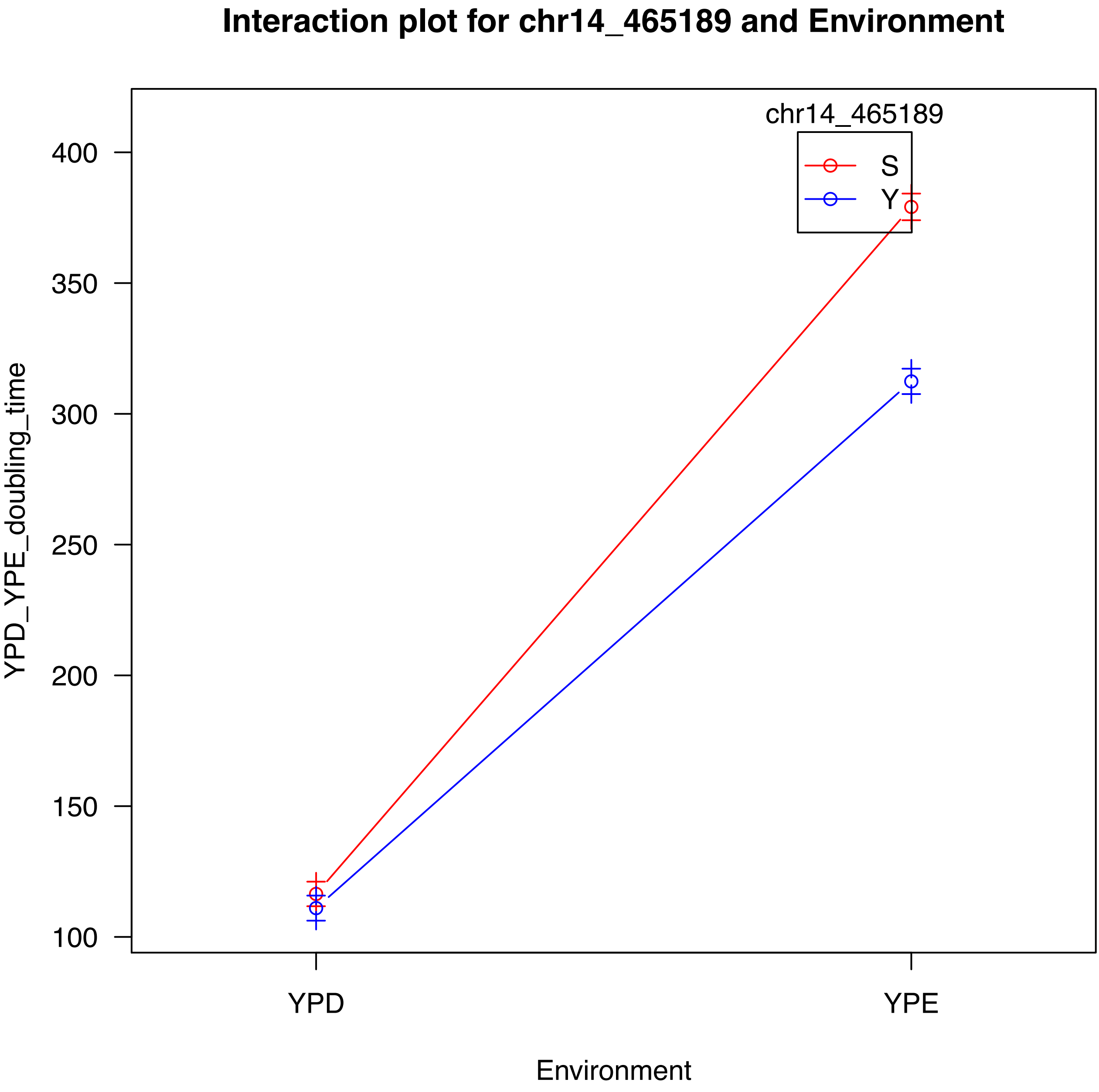

Interaction plot for chr14_465189 and Environment
chr14_465189
S
Y
400
350
300
250
200
150
100
YPD_YPE_doubling_time
YPD
YPE
Environment

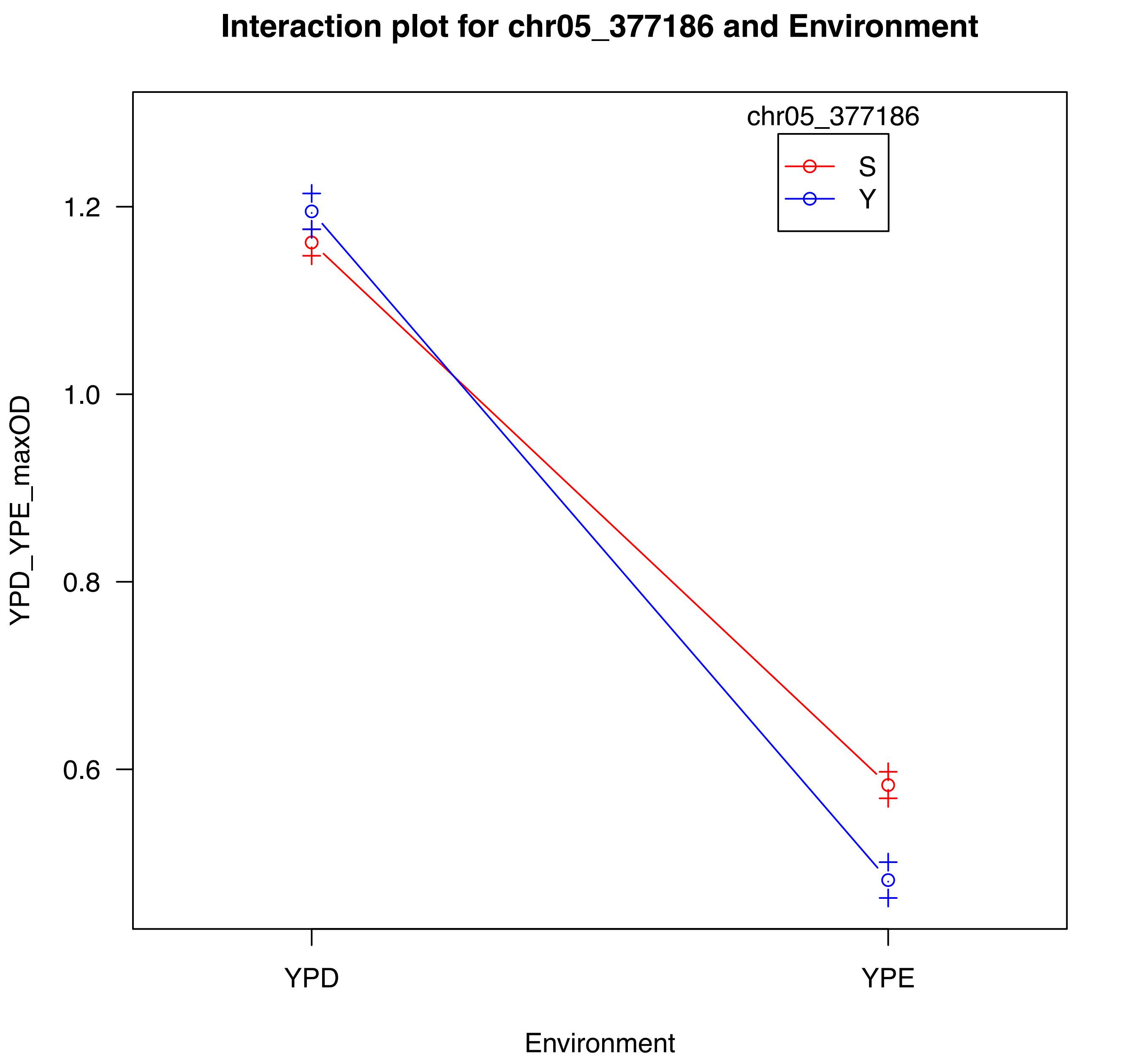

Interaction plot for chr05_377186 and Environment
chr05_377186
S
Y
1.2
1.0
0.8
0.6
YPD_YPE_maxOD
YPD
YPE
Environment

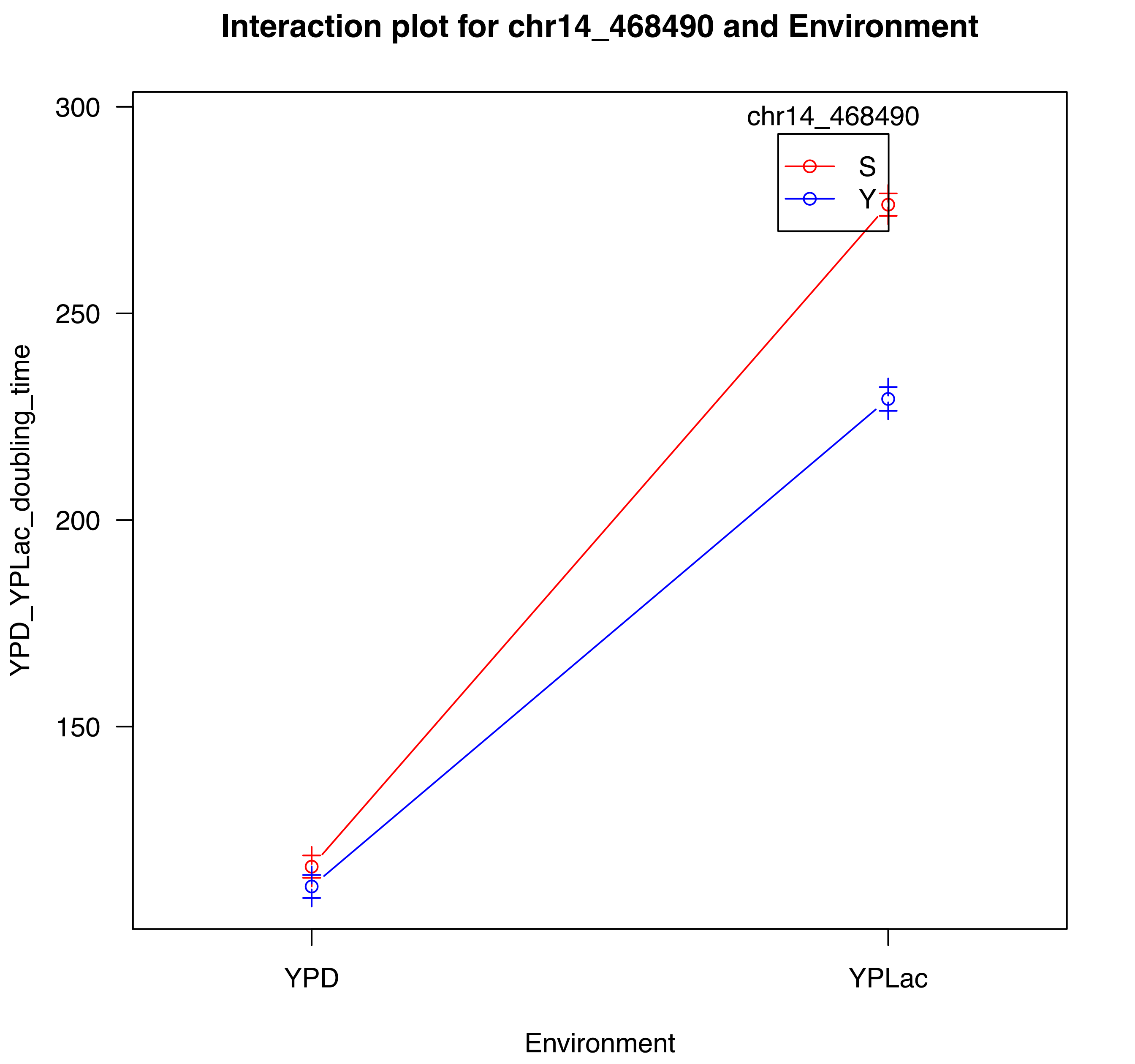

Interaction plot for chr14_468490 and Environment
chr14_468490
S
Y
300
250
200
150
YPD_YPLac_doubling_time
YPD
YPLac
Environment

Interaction plot for chr07_1069012 and Environment

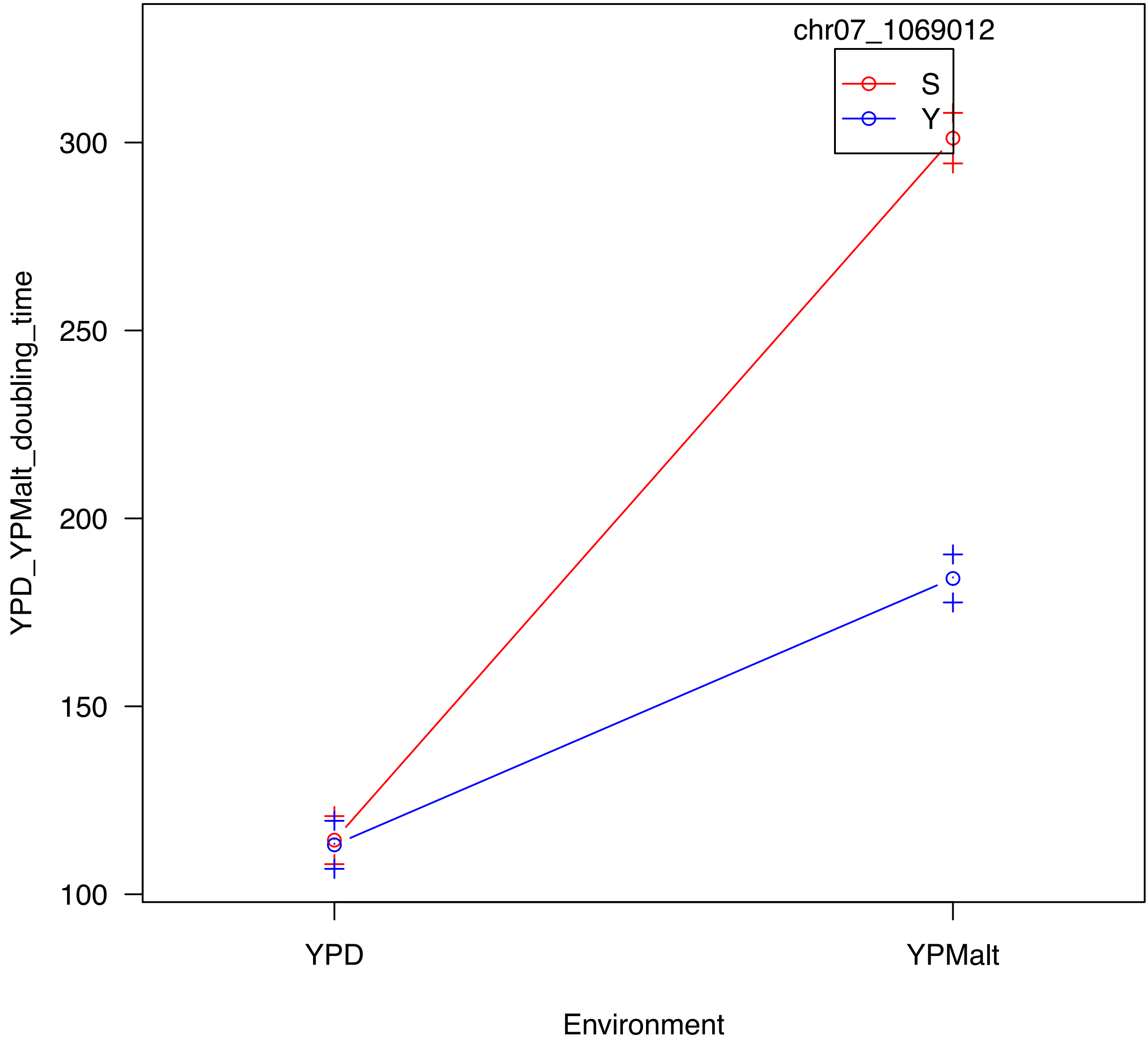

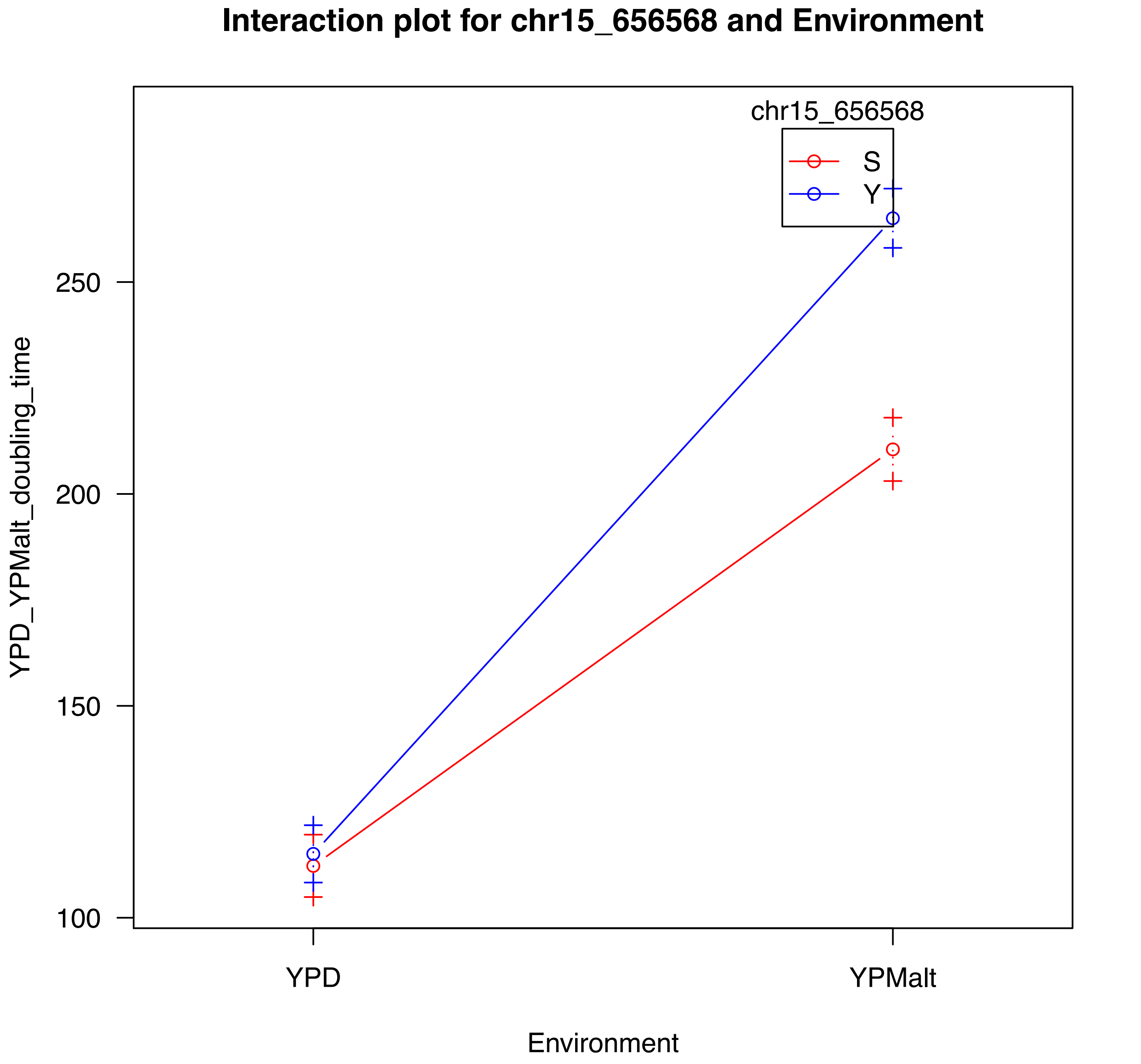

Interaction plot for chr15_656568 and Environment
chr15_656568
S
Y
YPD_YPMalt_doubling_time
250
200
150
100
YPD
YPMalt
Environment

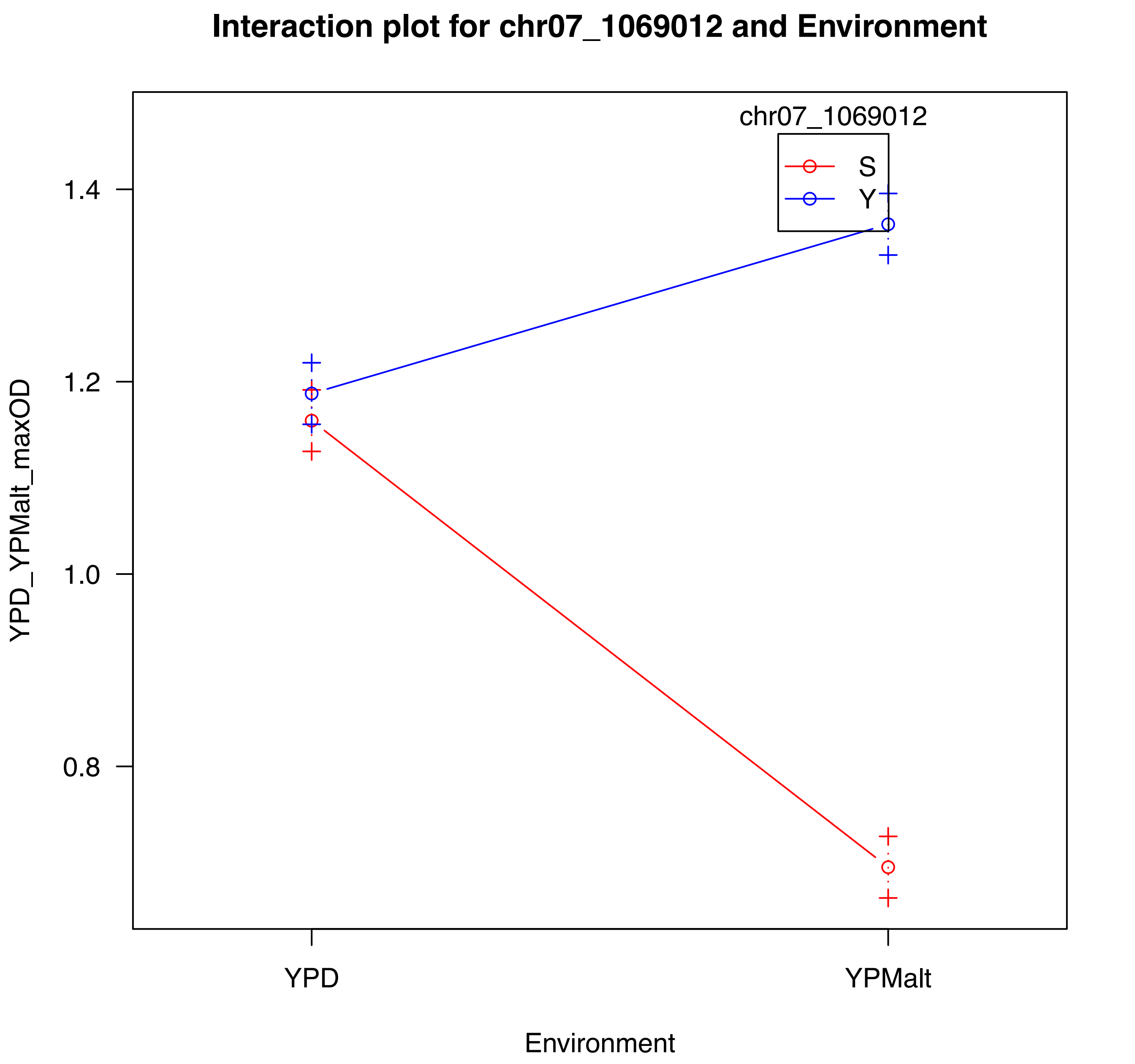

Interaction plot for chr07_1069012 and Environment
chr07_1069012
S
Y
1.4
1.2
1.0
0.8
YPD_YPMalt_maxOD
YPD
YPMalt
Environment

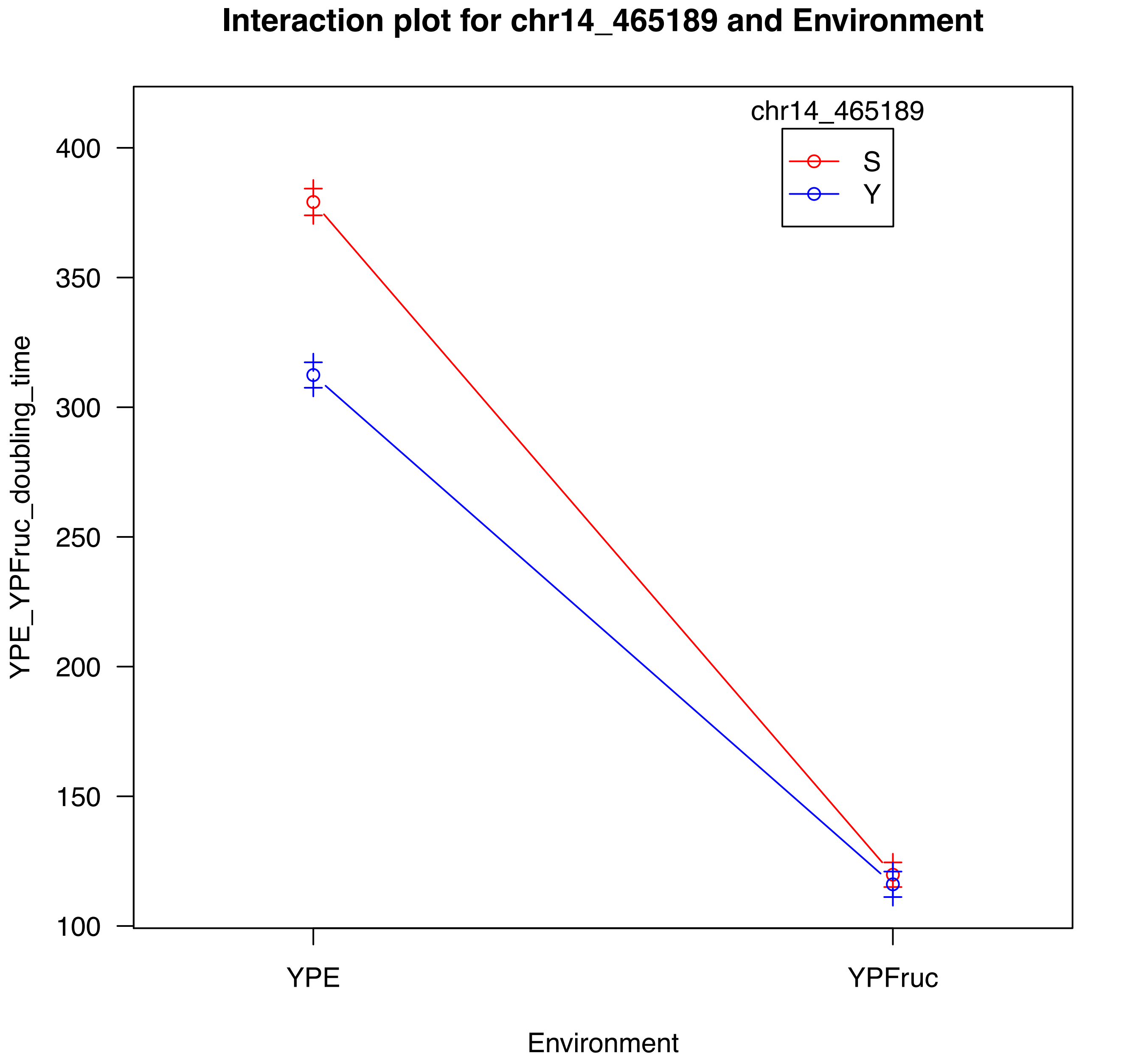

Interaction plot for chr14_465189 and Environment
chr14_465189
S
Y
400
350
300
250
200
150
100
YPE_YPFruc_doubling_time
YPE
YPFruc
Environment

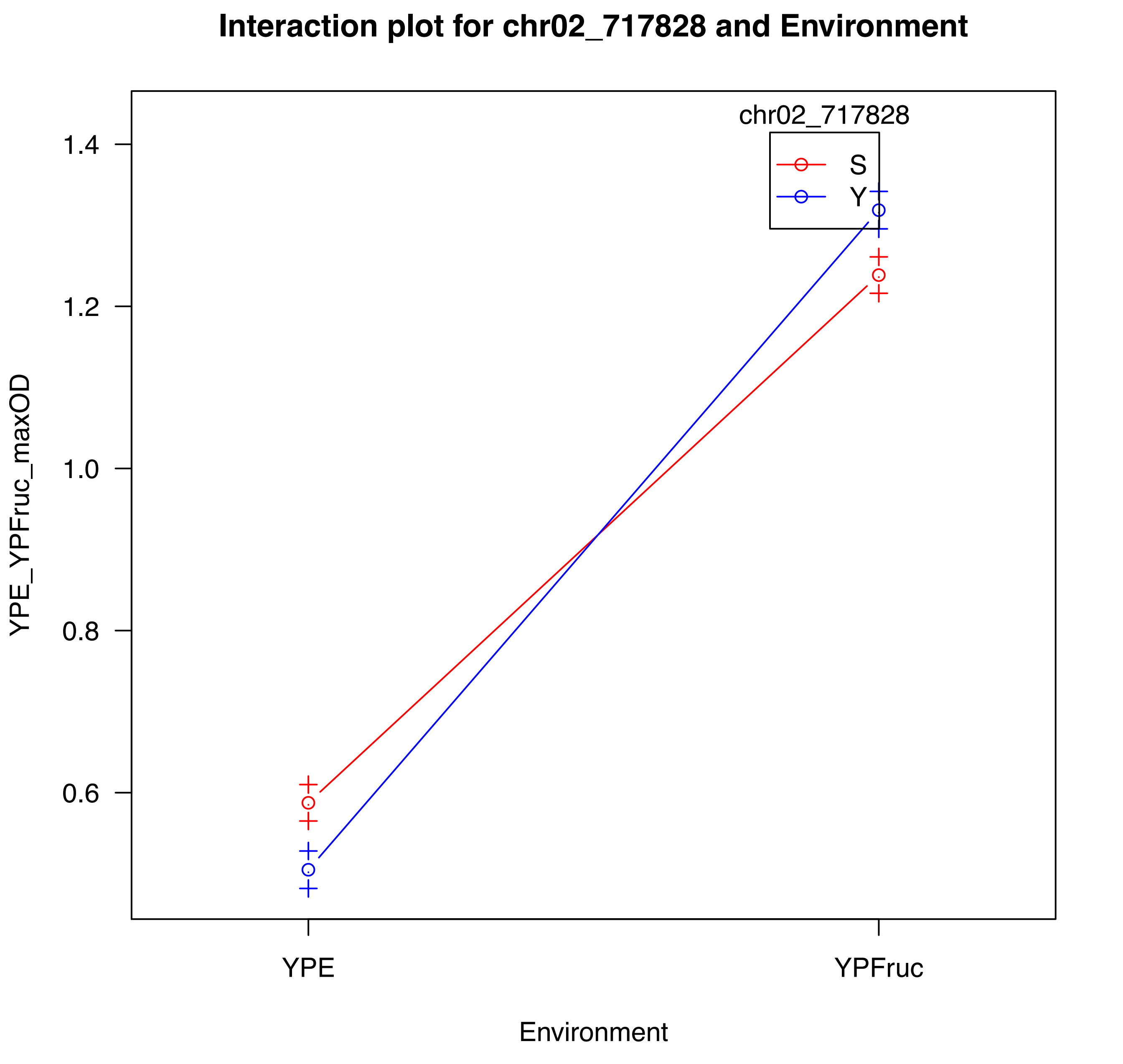

Interaction plot for chr02_717828 and Environment
chr02_717828
S
Y
1.4
1.2
1.0
0.8
0.6
YPE_YPFruc_maxOD
YPE
YPFruc
Environment

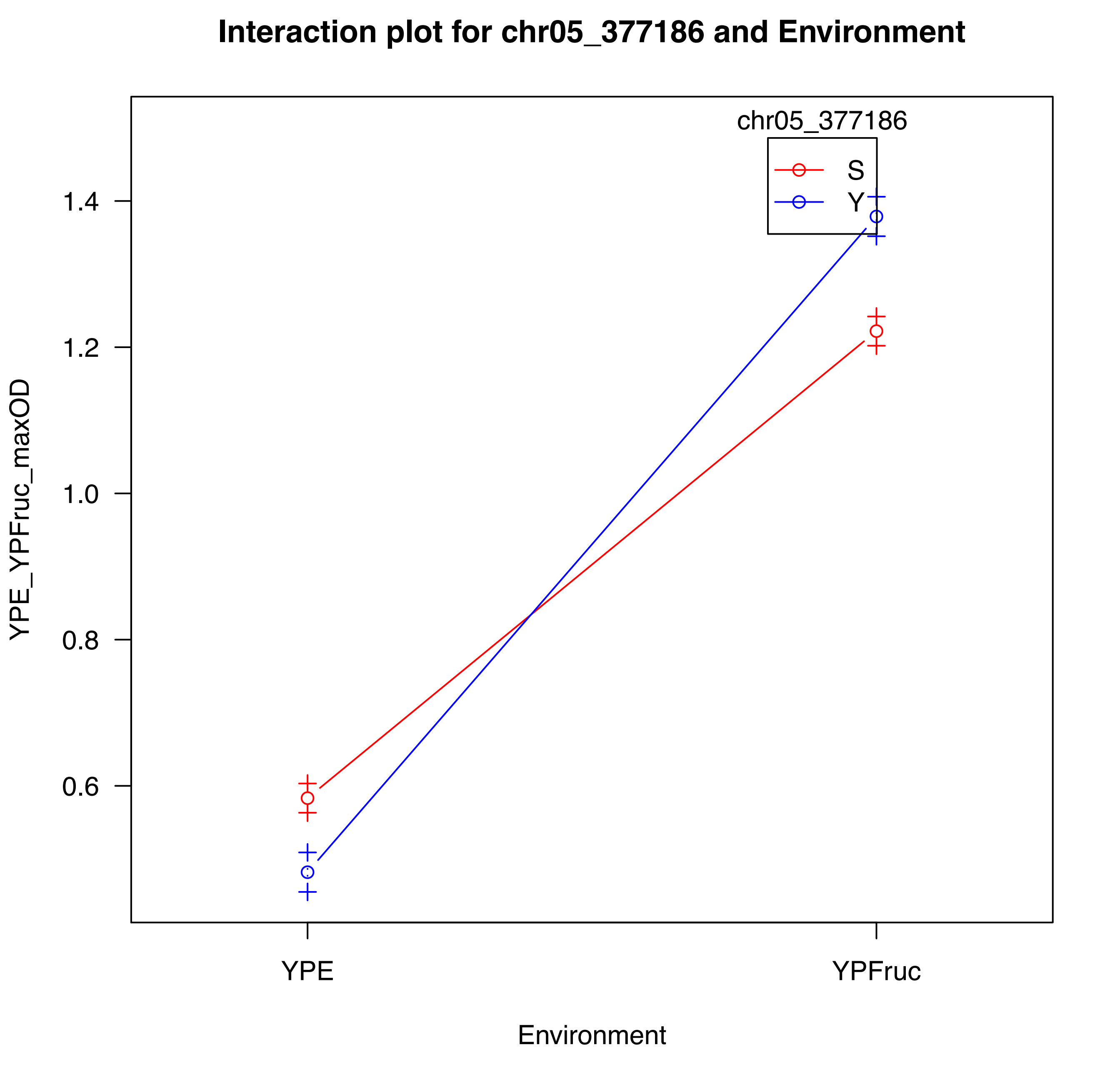

Interaction plot for chr05_377186 and Environment
chr05_377186
S
Y
1.4
1.2
1.0
0.8
0.6
YPE_YPFruc_maxOD
YPE
YPFruc
Environment

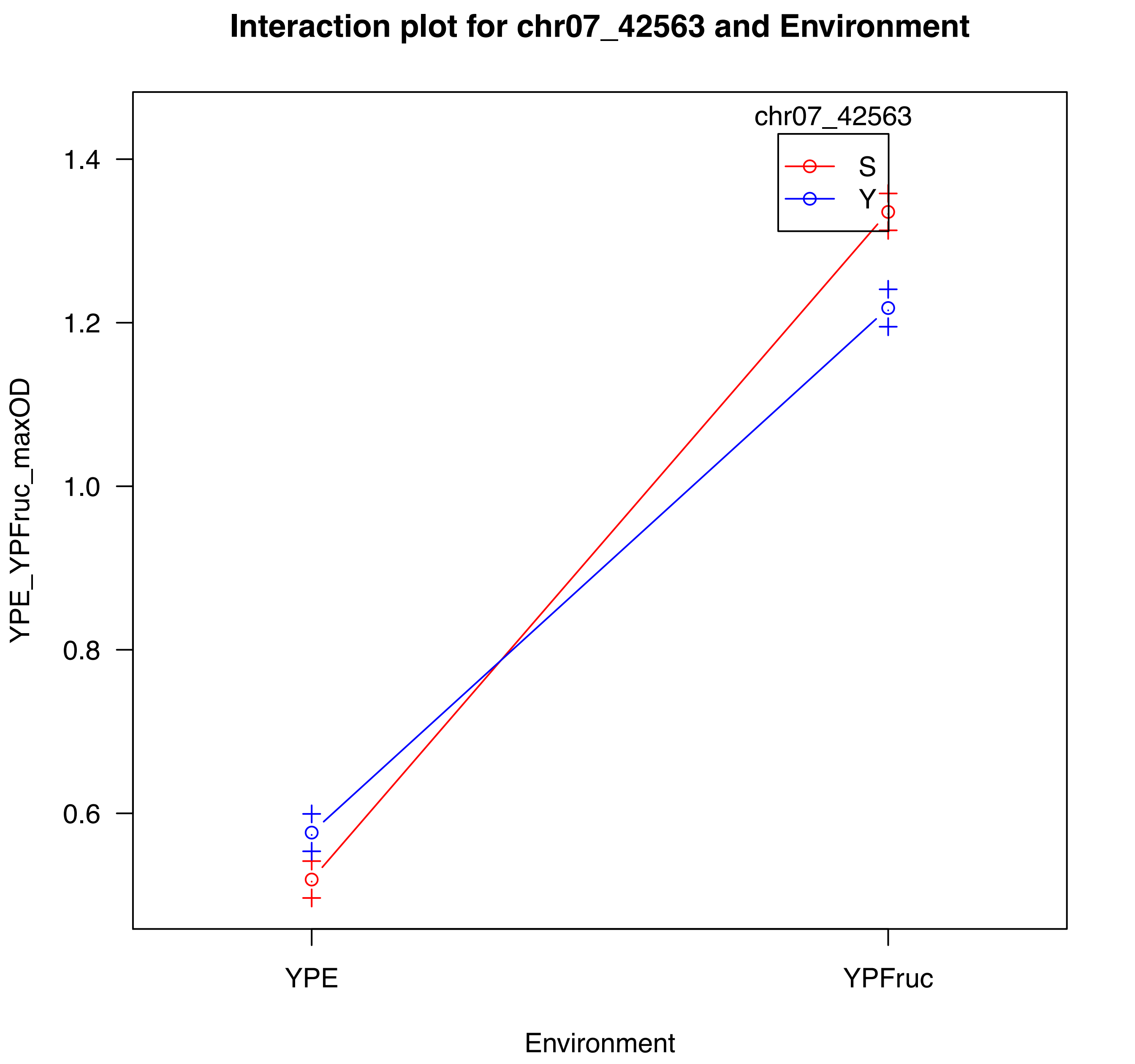

Interaction plot for chr07_42563 and Environment
chr07_42563
S
Y
1.4
1.2
1.0
0.8
0.6
YPE_YPFruc_maxOD
YPE
YPFruc
Environment

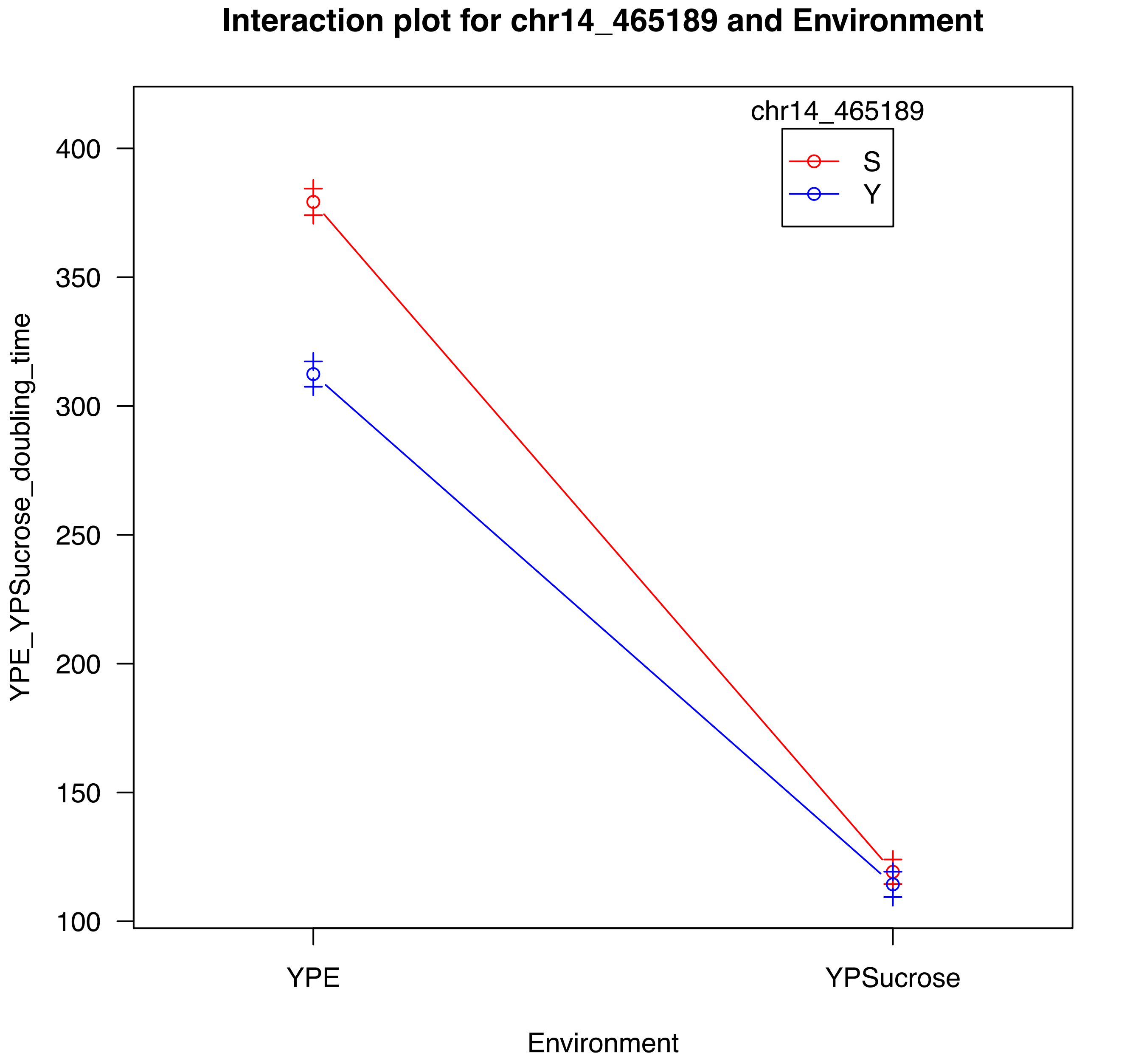

Interaction plot for chr14_465189 and Environment
chr14_465189
S
Y
400
350
300
250
200
150
100
YPE_YPSucrose_doubling_time
YPE
YPSucrose
Environment

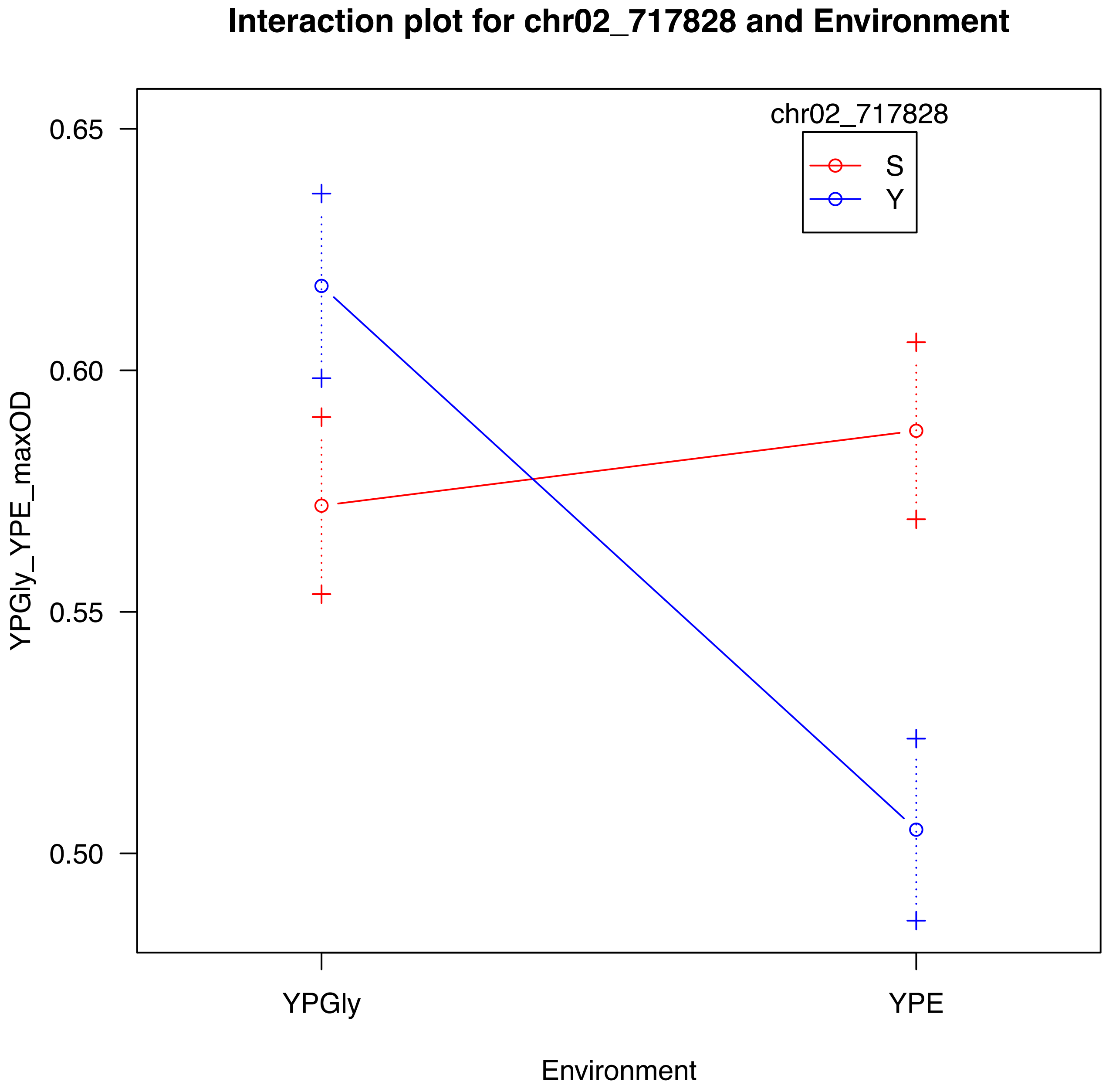
Interaction plot for chr02_717828 and Environment
chr02_717828
S
Y
0.65
0.60
0.55
0.50
YPGly_YPE_maxOD
YPGly
YPE
Environment

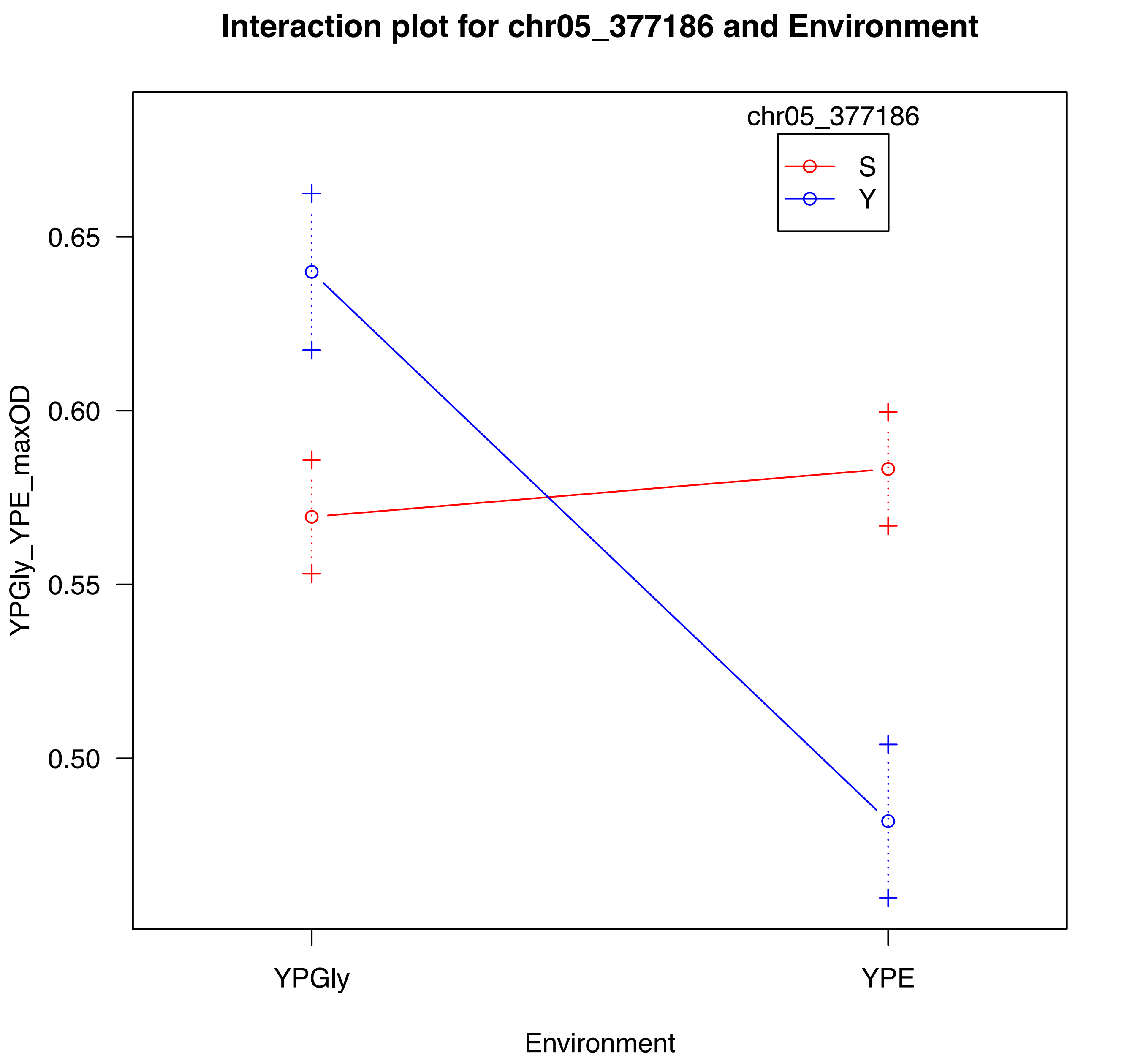

Interaction plot for chr05_377186 and Environment
chr05_377186
S
Y
0.65
0.60
0.55
0.50
YPGly_YPE_maxOD
YPGly
YPE
Environment

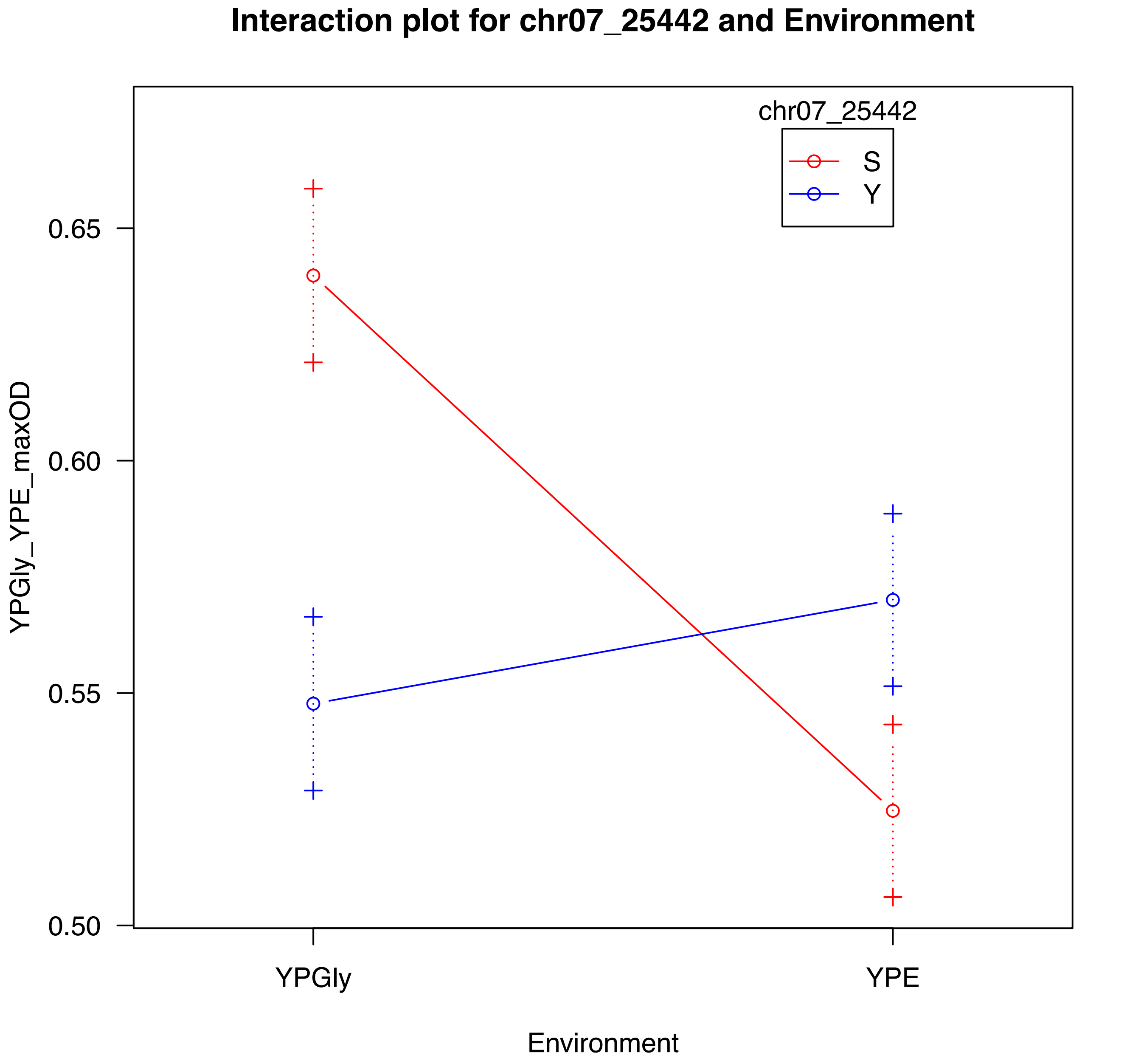

Interaction plot for chr07_25442 and Environment
chr07_25442
S
Y
0.65
0.60
0.55
0.50
YPGly_YPE_maxOD
YPGly
YPE
Environment

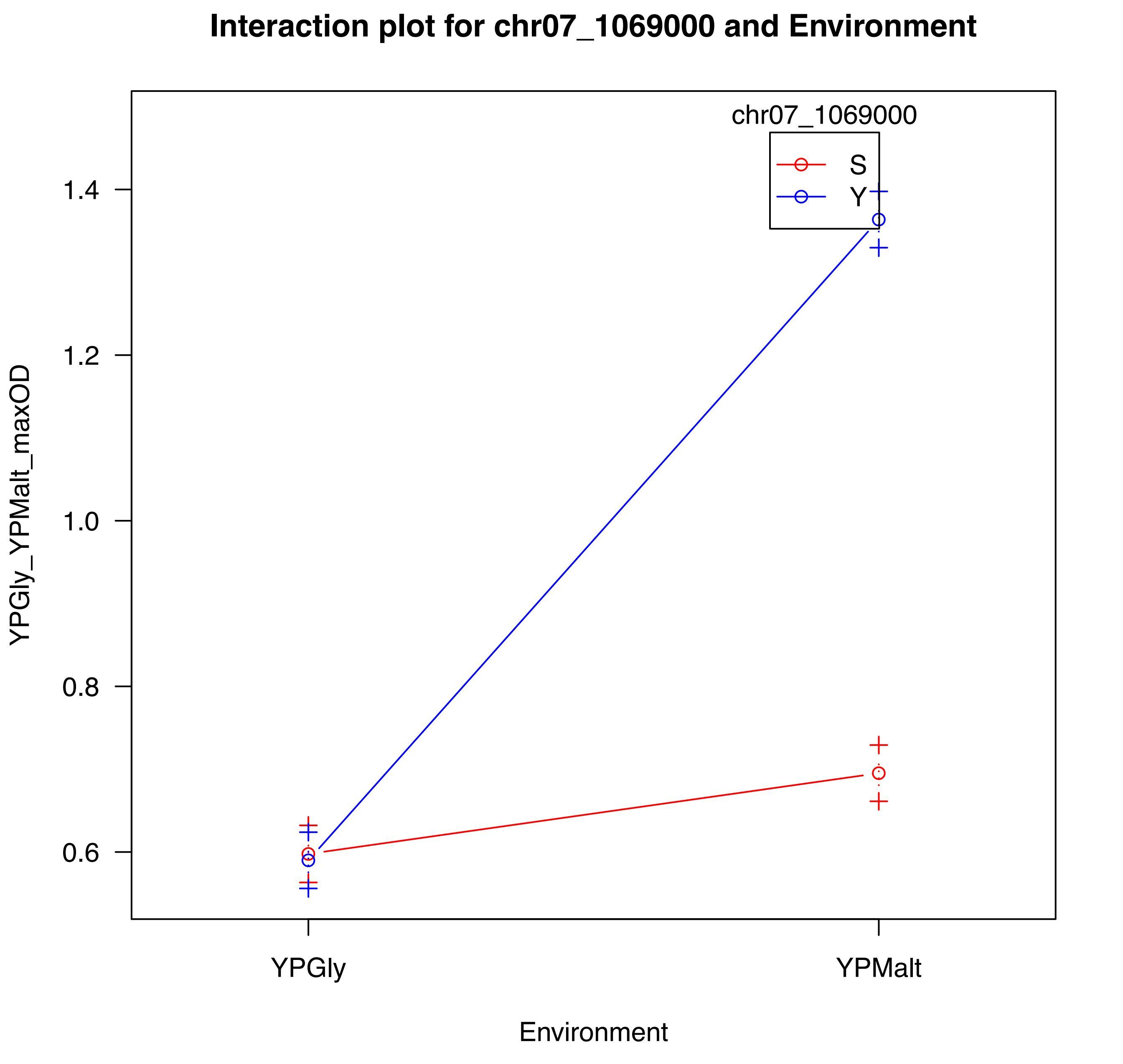

Interaction plot for chr07_1069000 and Environment
chr07_1069000
S
Y
1.4
1.2
1.0
0.8
0.6
YPGly_YPMalt_maxOD
YPGly
YPMalt
Environment

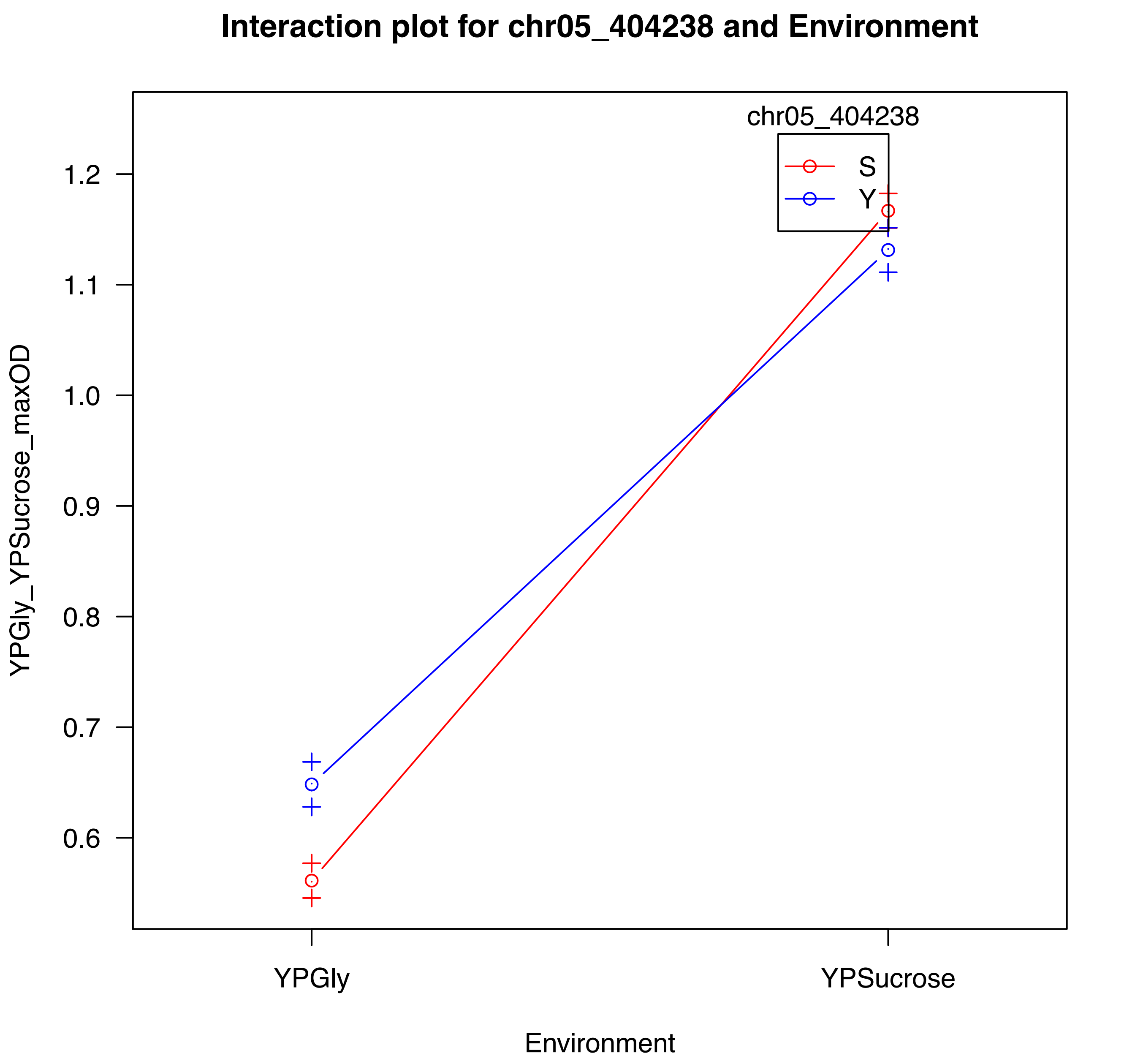

Interaction plot for chr05_404238 and Environment
chr05_404238
S
Y
YPGly_YPSucrose_maxOD
1.2
1.1
1.0
0.9
0.8
0.7
0.6
YPGly
YPSucrose
Environment

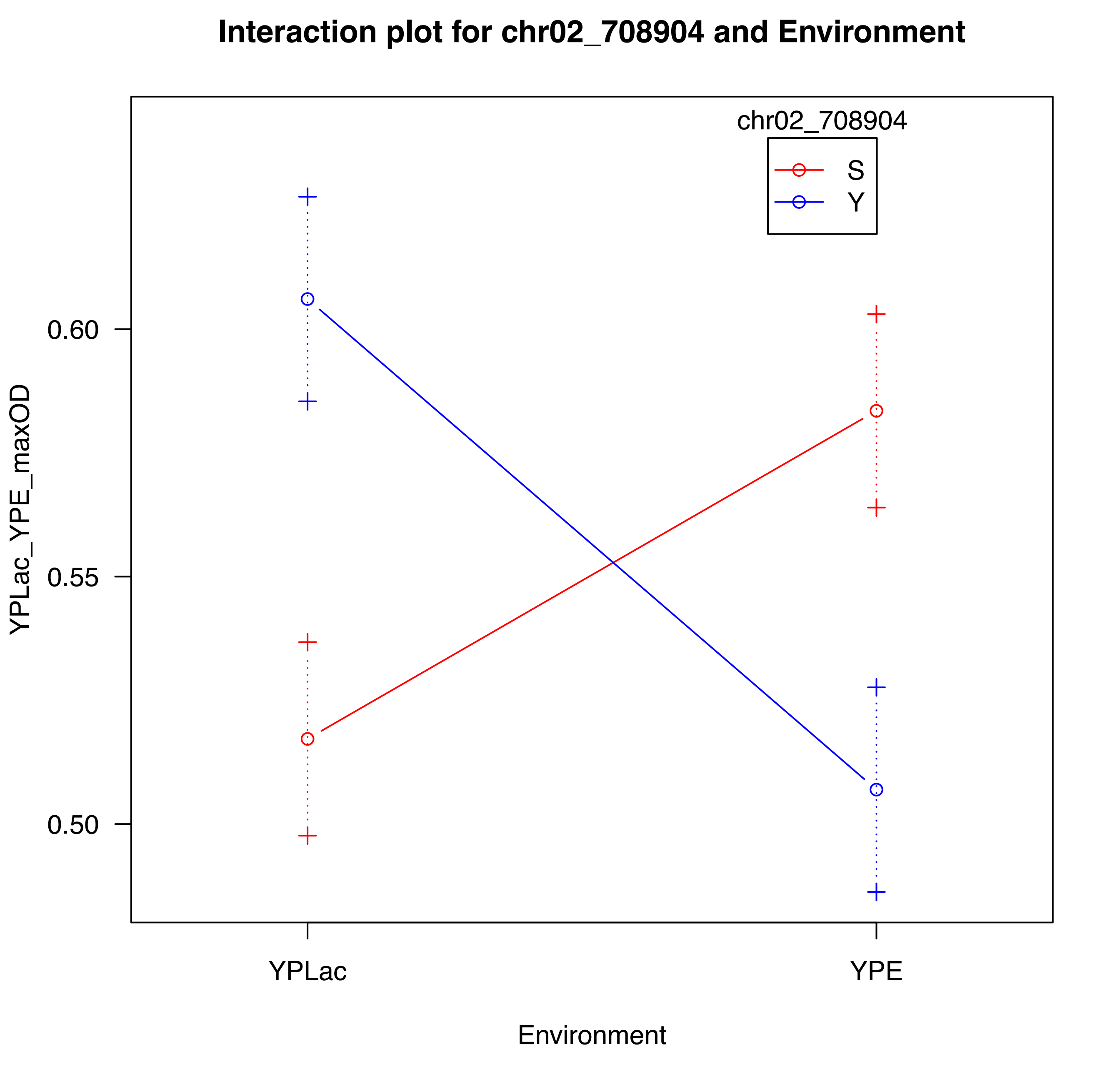

Interaction plot for chr02_708904 and Environment
chr02_708904
S
Y
0.60
0.55
0.50
YPLac_YPE_maxOD
YPLac
YPE
Environment

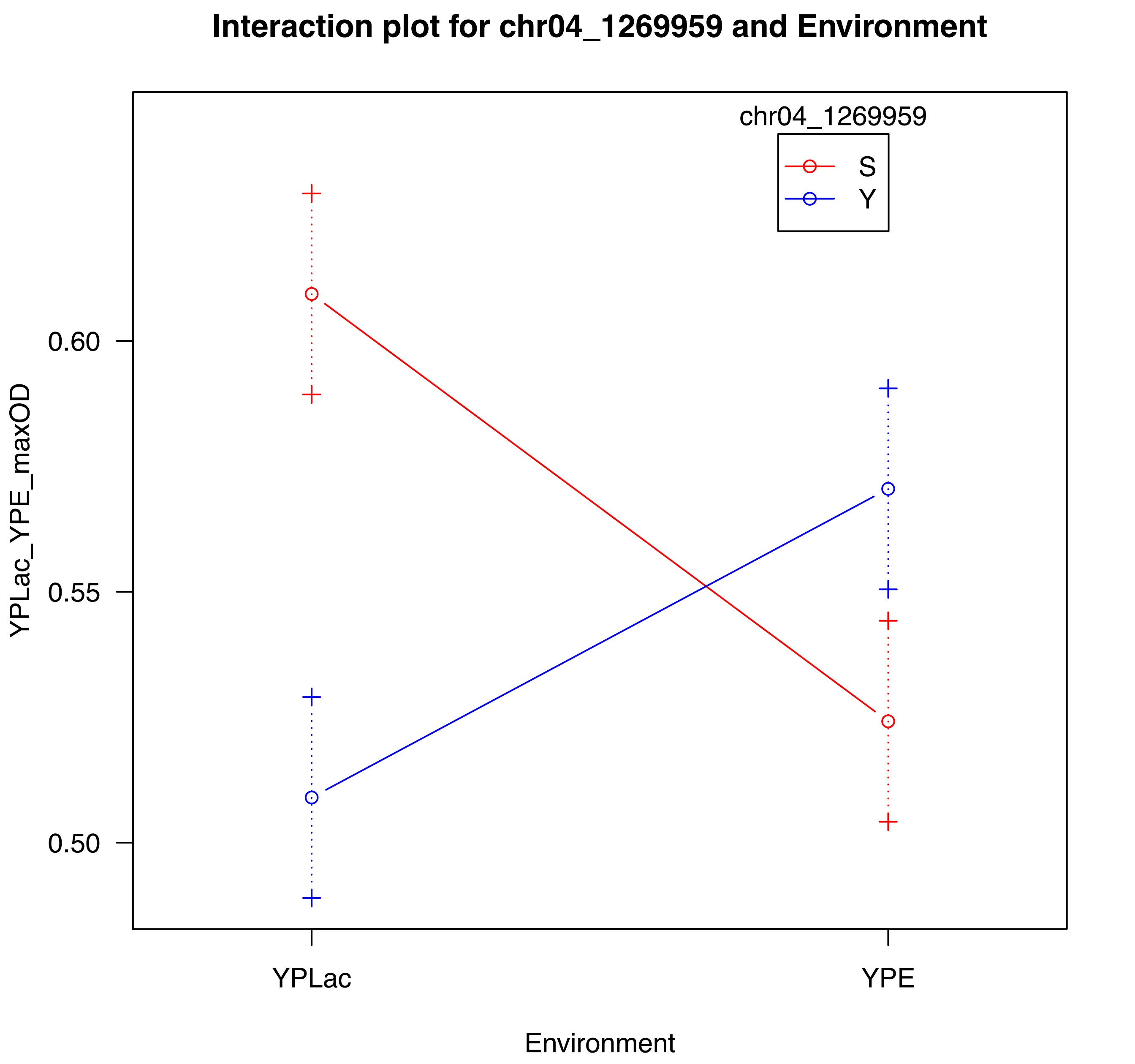
Interaction plot for chr04_1269959 and Environment
chr04_1269959
S
Y
0.60
0.55
0.50
YPLac_YPE_maxOD
YPLac
YPE
Environment

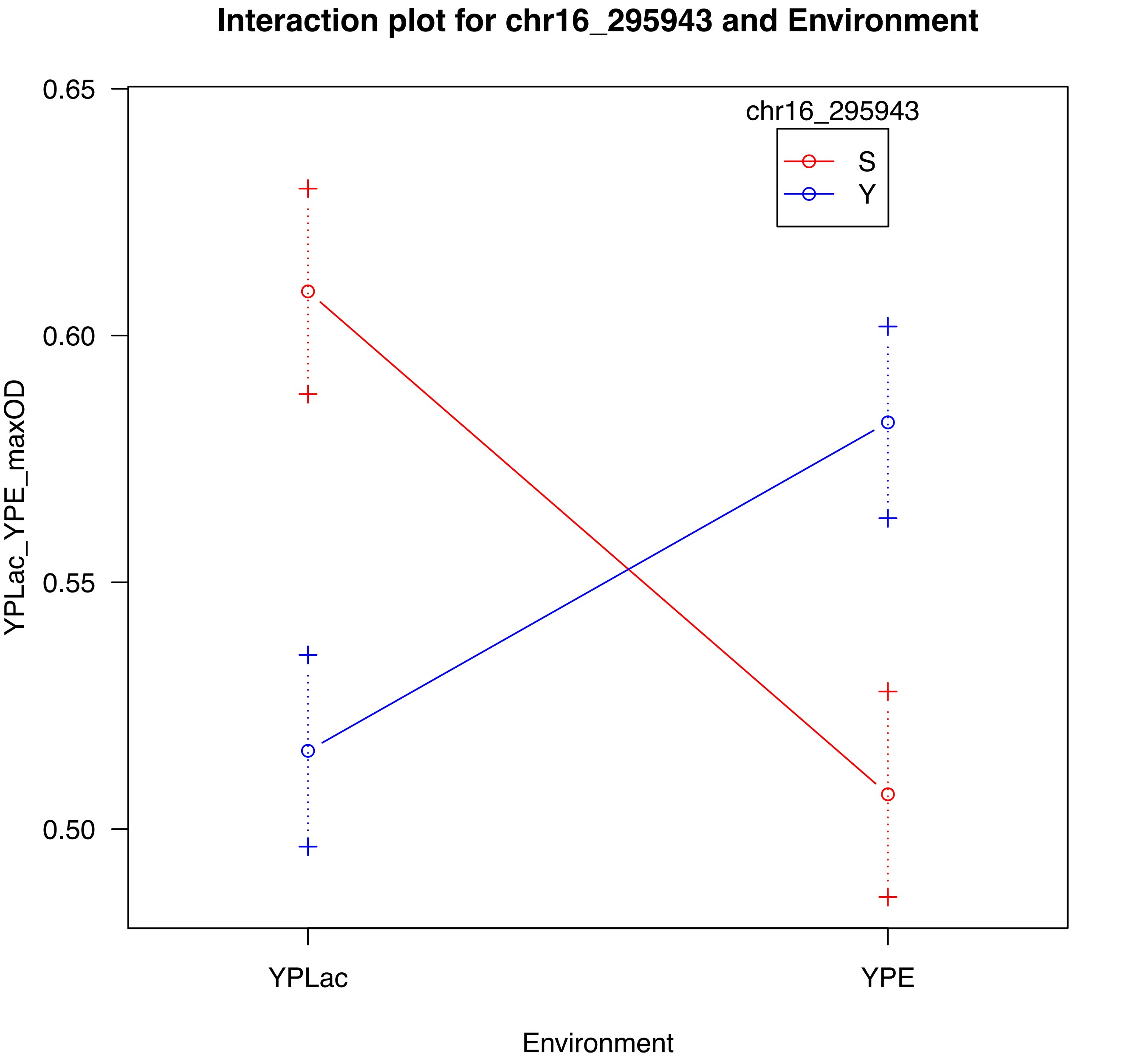
Interaction plot for chr16_295943 and Environment
chr16_295943
S
Y
0.65
0.60
0.55
0.50
YPLac_YPE_maxOD
YPLac
YPE
Environment

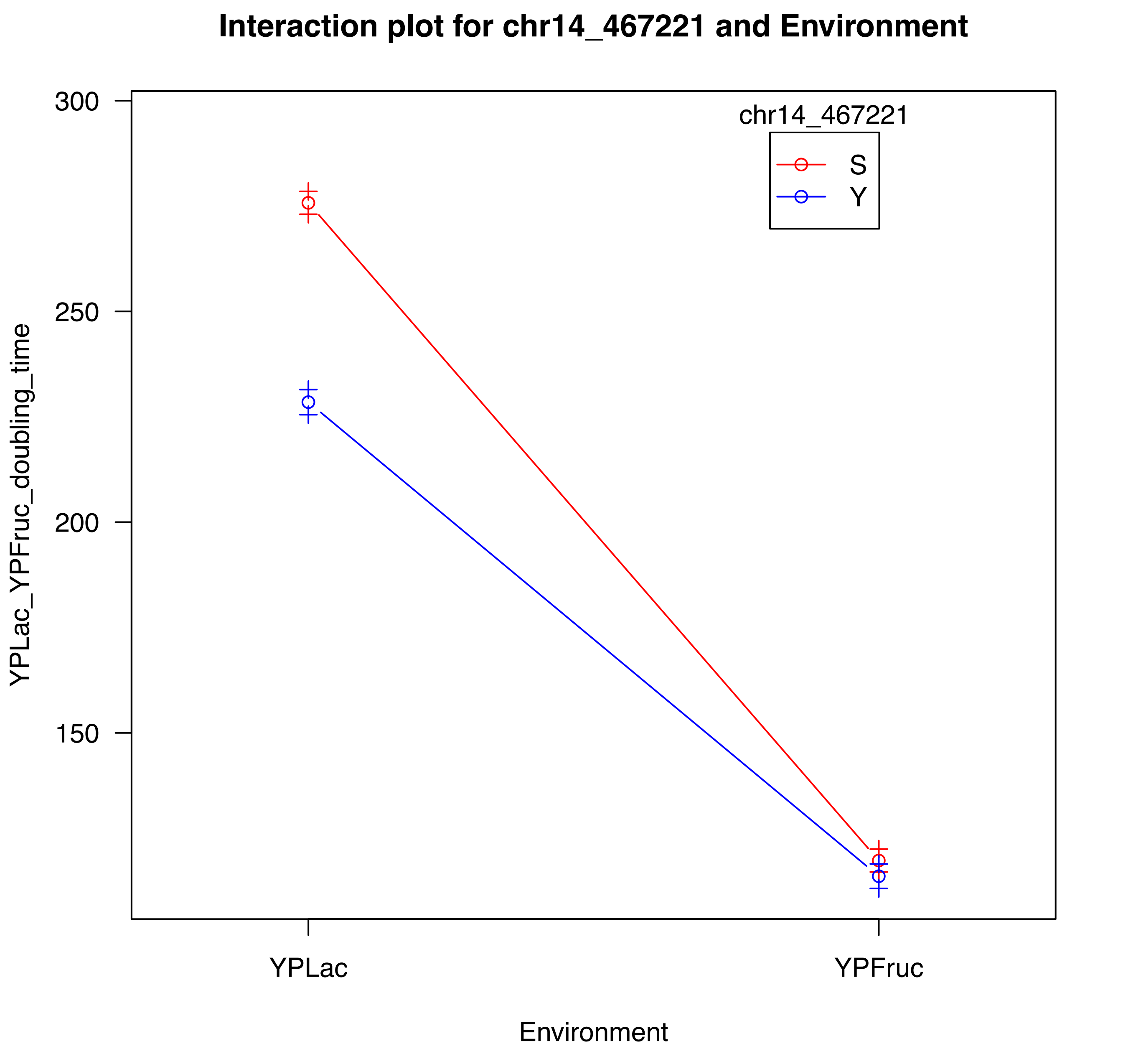

Interaction plot for chr14_467221 and Environment
300
250
200
150
YPLac_YPFruc_doubling_time
chr14_467221
S
Y
YPLac
YPFruc
Environment

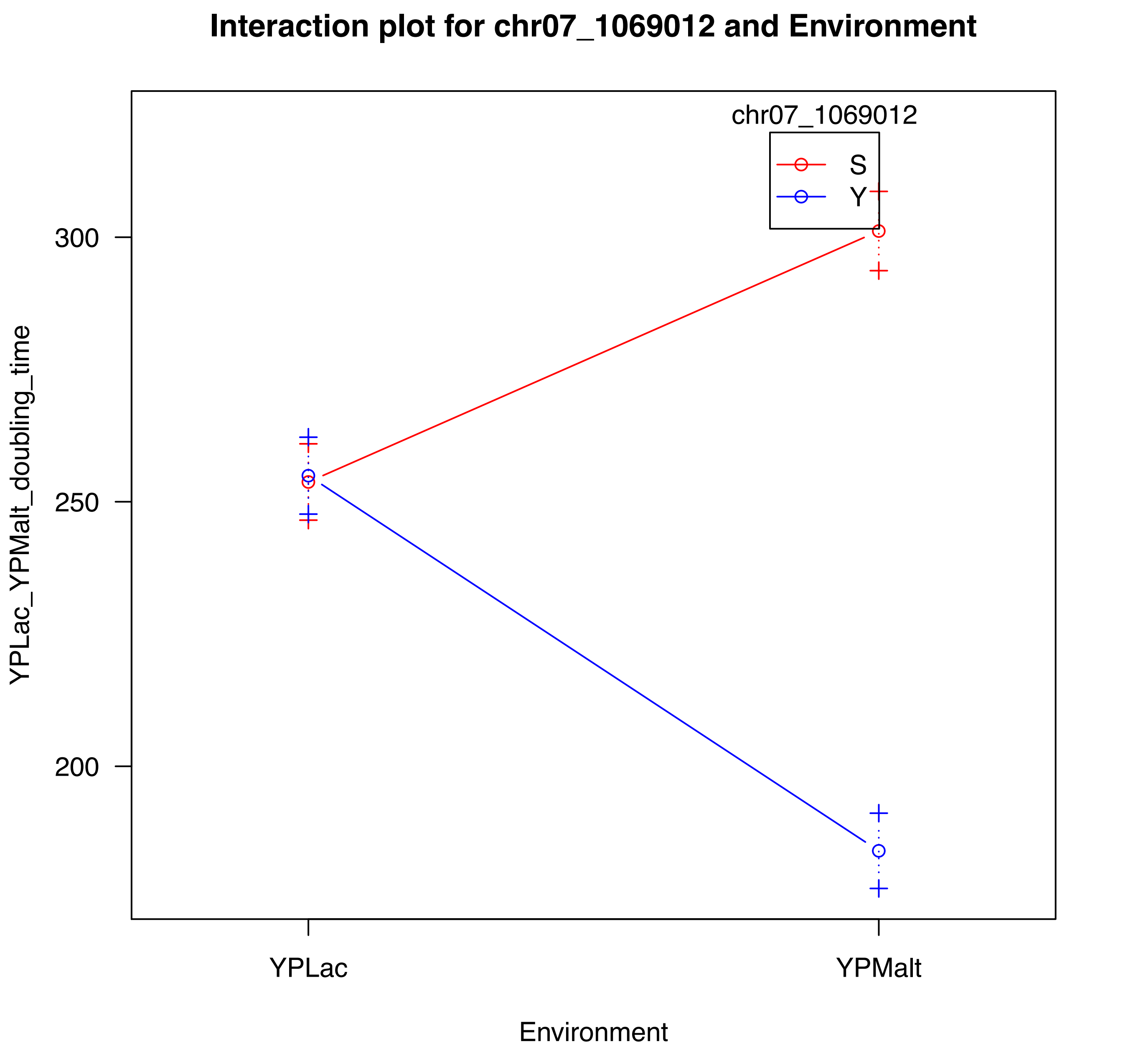

Interaction plot for chr07_1069012 and Environment
chr07_1069012
S
Y
300
250
200
YPLac_YPMalt_doubling_time
YPLac
YPMalt
Environment

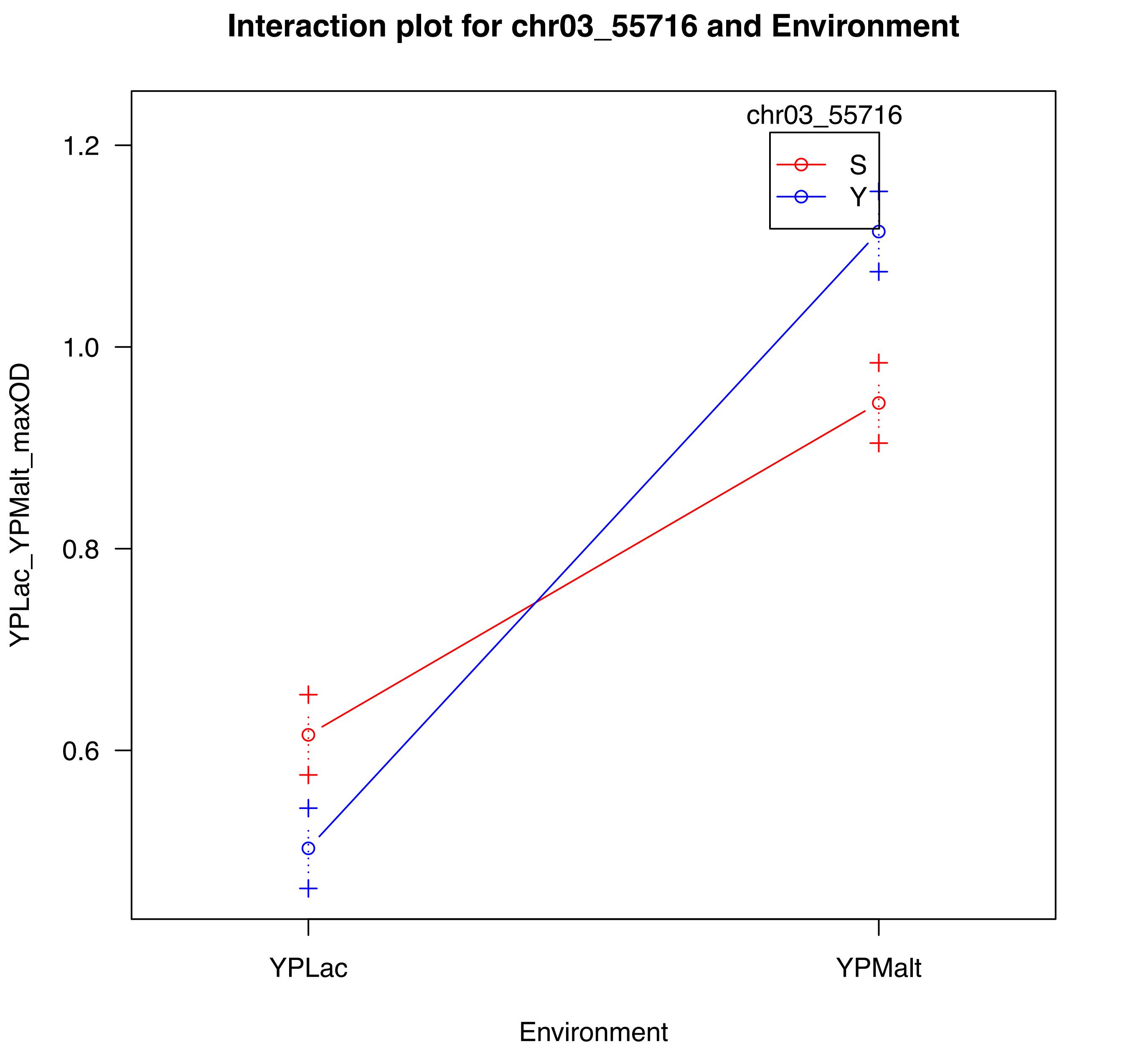
Interaction plot for chr03_55716 and Environment
chr03_55716
S
Y
YPLac_YPMalt_maxOD
1.2
1.0
0.8
0.6
YPLac
YPMalt
Environment

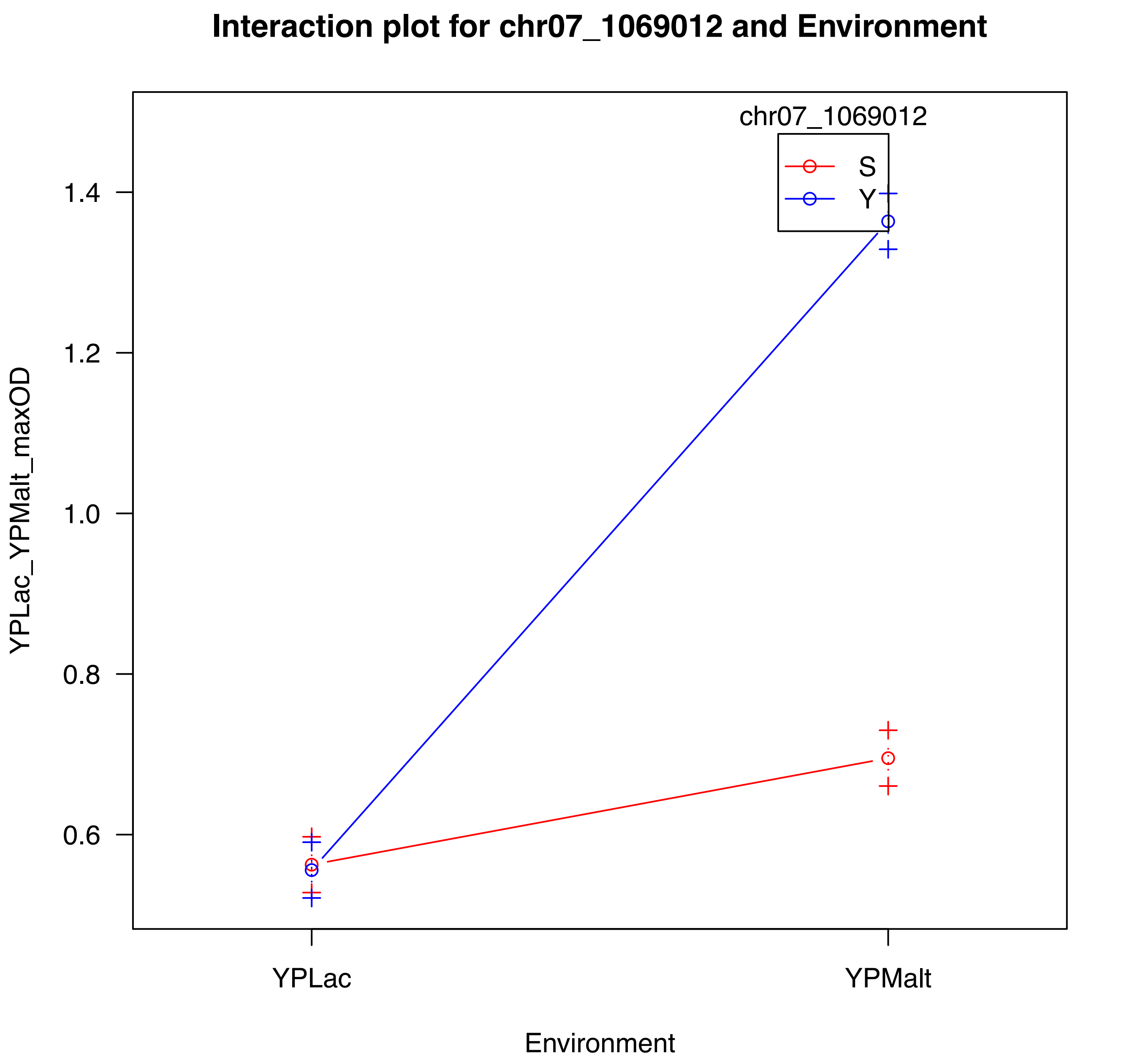

Interaction plot for chr07_1069012 and Environment
chr07_1069012
S
Y
1.4
1.2
1.0
0.8
0.6
YPLac_YPMalt_maxOD
YPLac
YPMalt
Environment

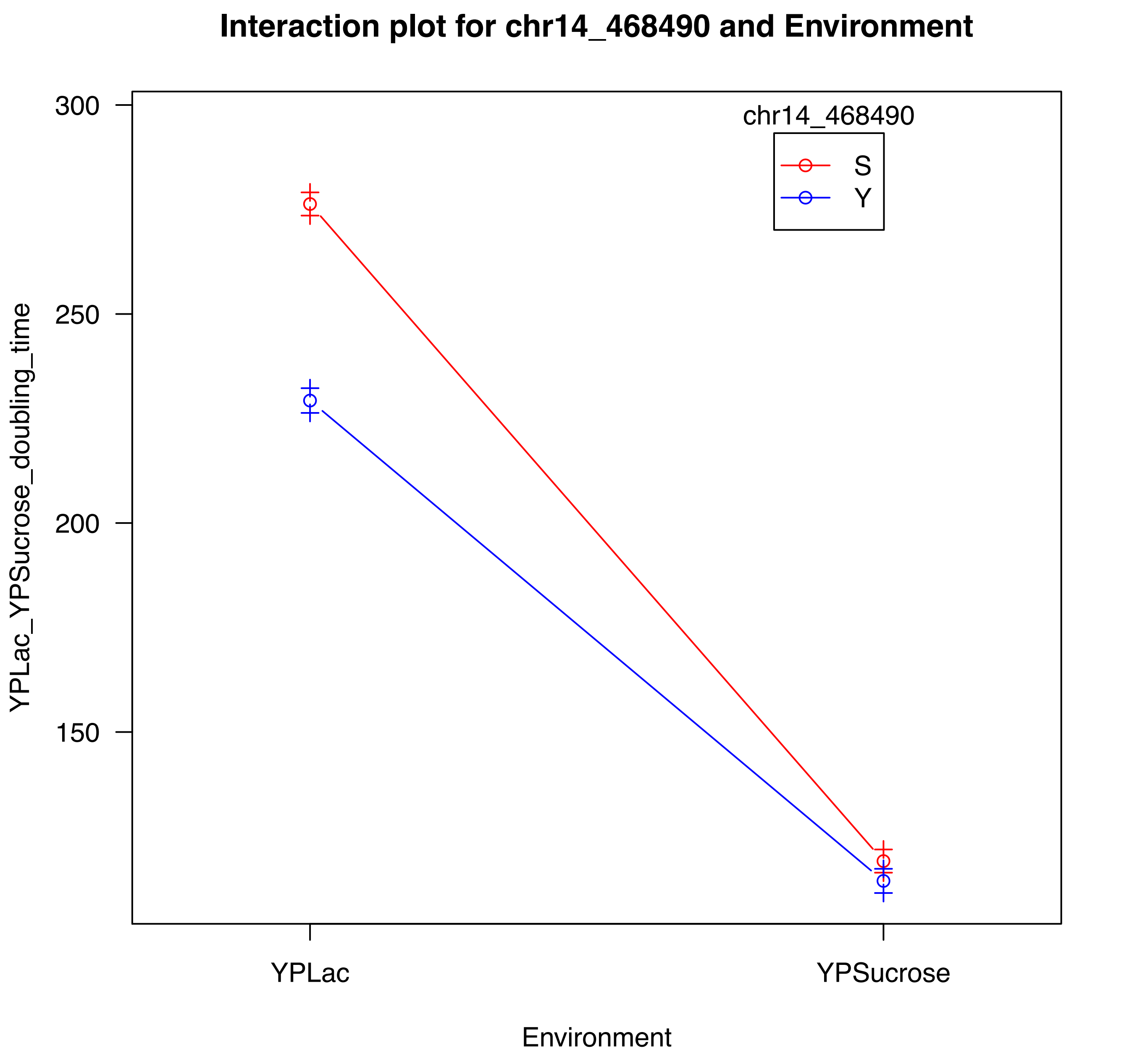
Interaction plot for chr14_468490 and Environment
chr14_468490
S
Y
300
250
200
150
YPLac_YPSucrose_doubling_time
YPLac
YPSucrose
Environment

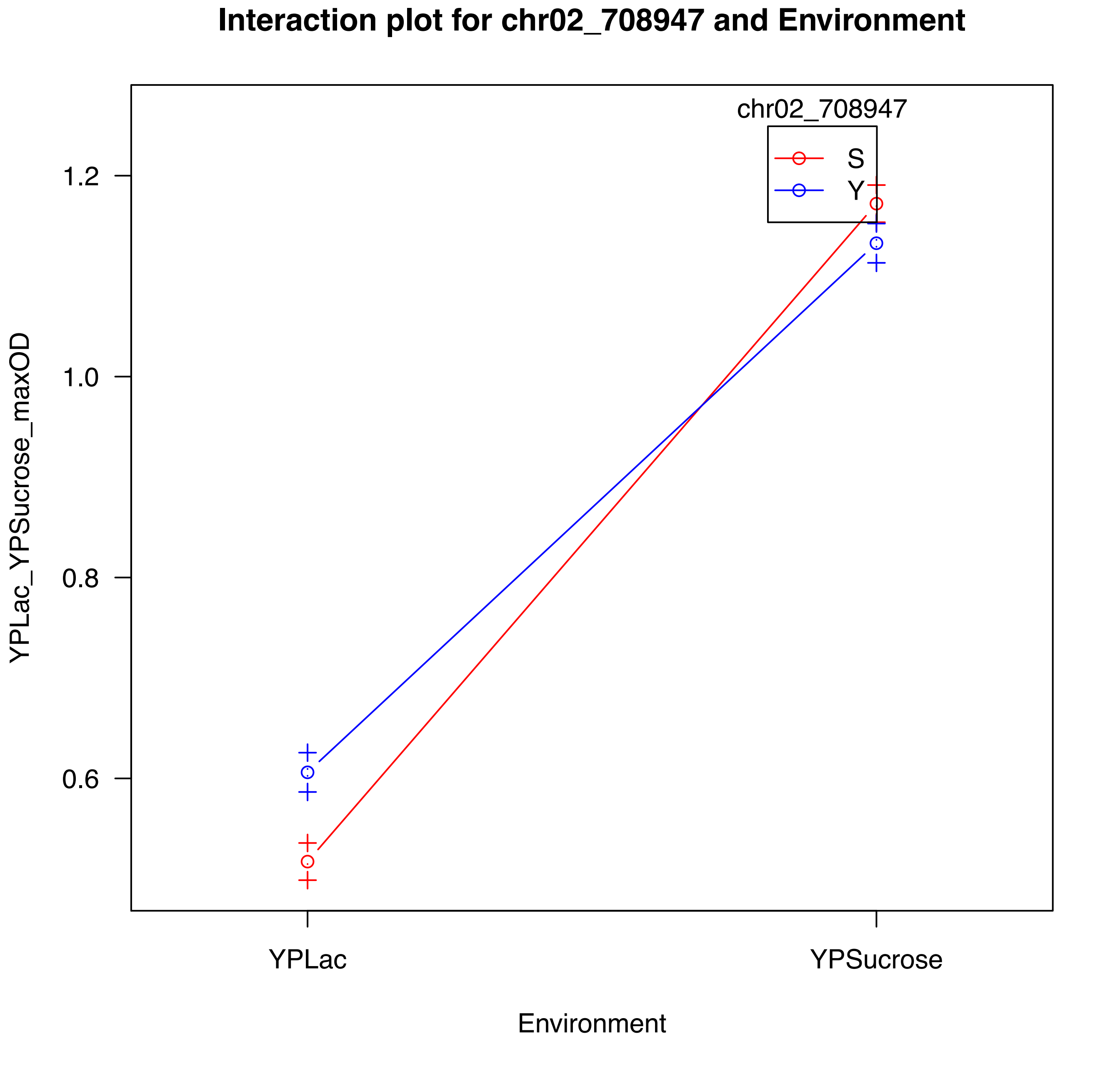
Interaction plot for chr02_708947 and Environment
chr02_708947
S
Y
1.2
1.0
0.8
0.6
YPLac_YPSucrose_maxOD
YPLac
YPSucrose
Environment

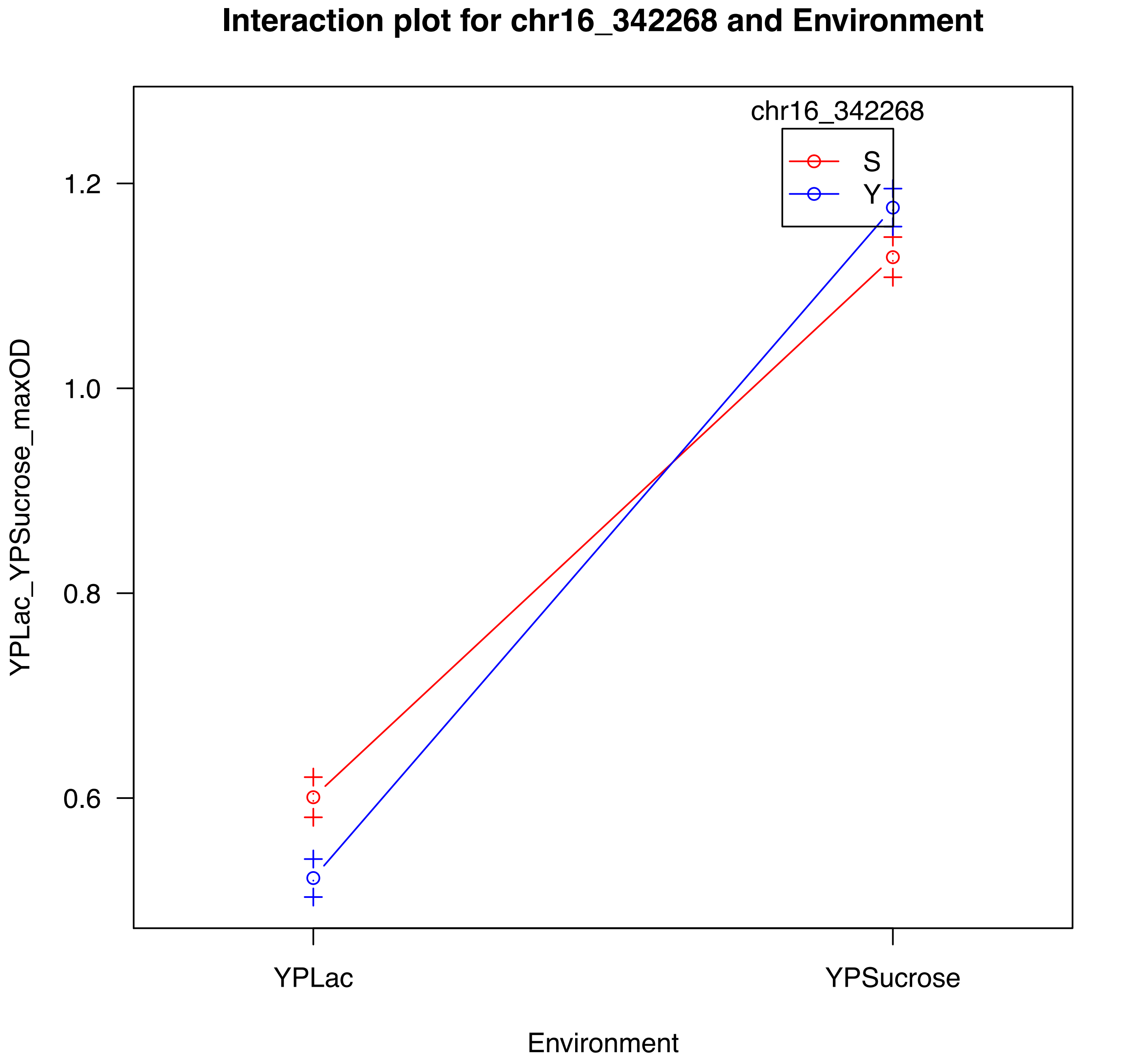

Interaction plot for chr16_342268 and Environment
chr16_342268
S
Y
1.2
1.0
0.8
0.6
YPLac_YPSucrose_maxOD
YPLac
YPSucrose
Environment

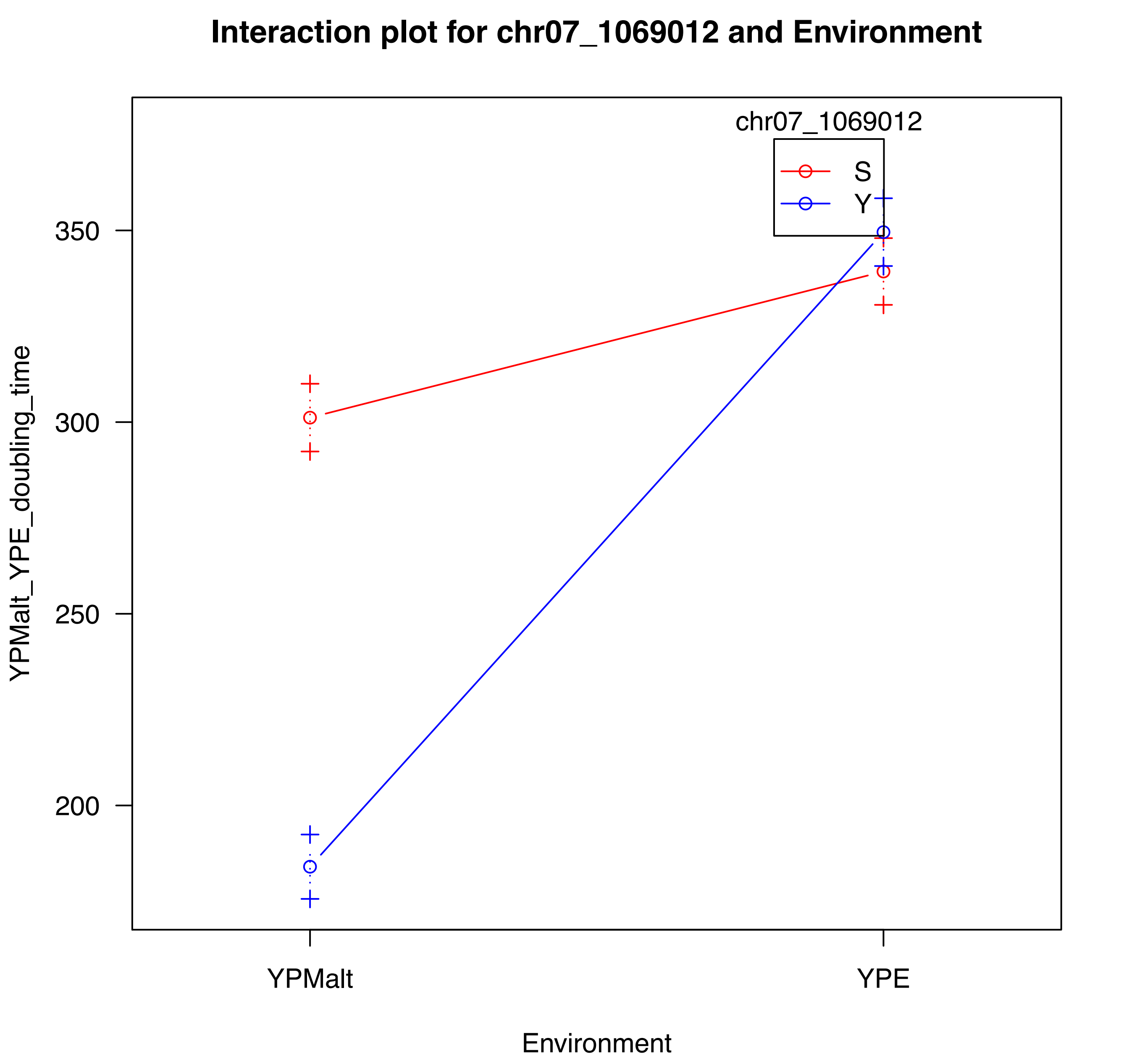

Interaction plot for chr07_1069012 and Environment
chr07_1069012
S
Y
350
300
250
200
YPMalt_YPE_doubling_time
YPMalt
YPE
Environment

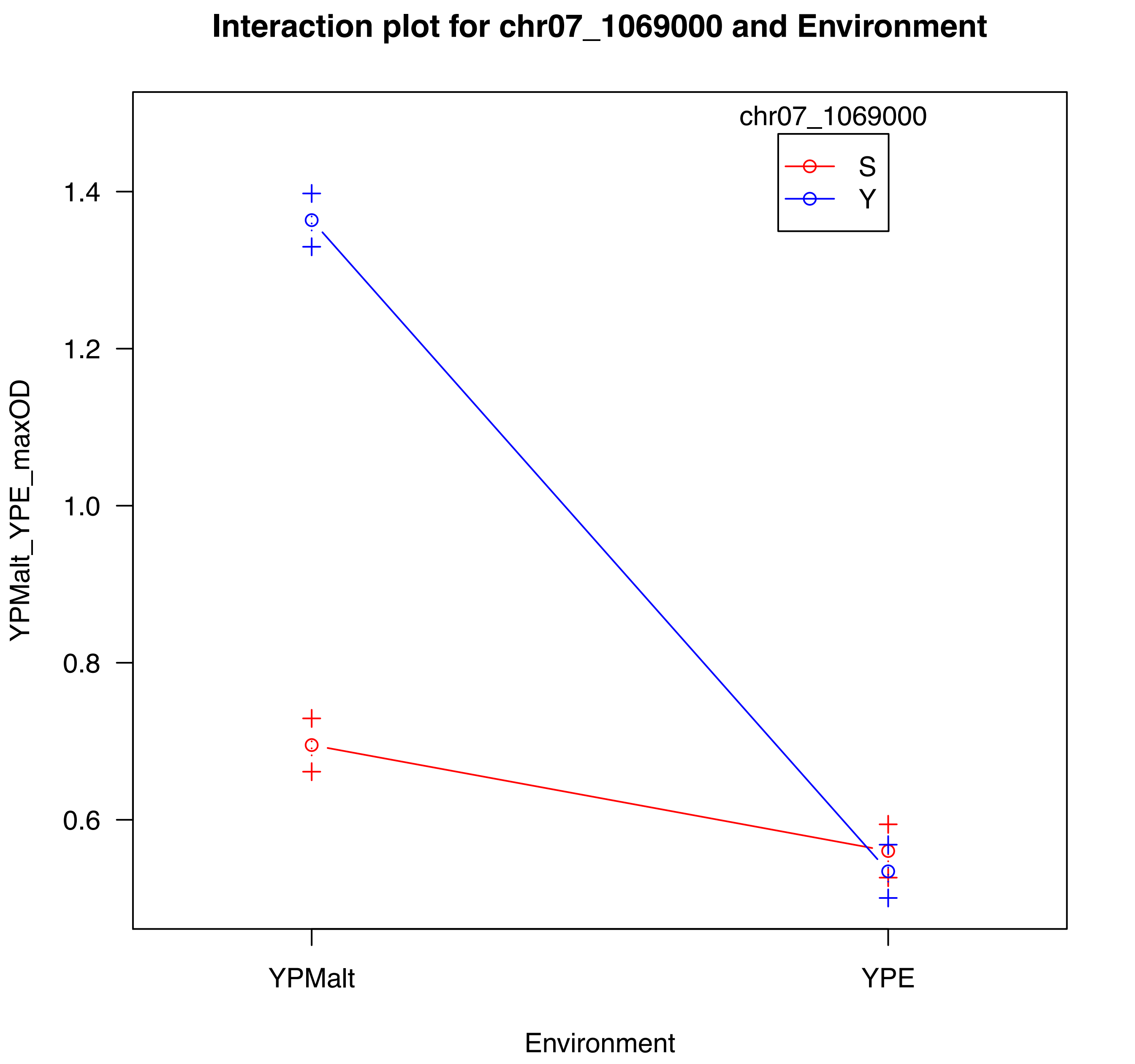

Interaction plot for chr07_1069000 and Environment
chr07_1069000
S
Y
1.4
1.2
1.0
0.8
0.6
YPMalt_YPE_maxOD
YPMalt
YPE
Environment

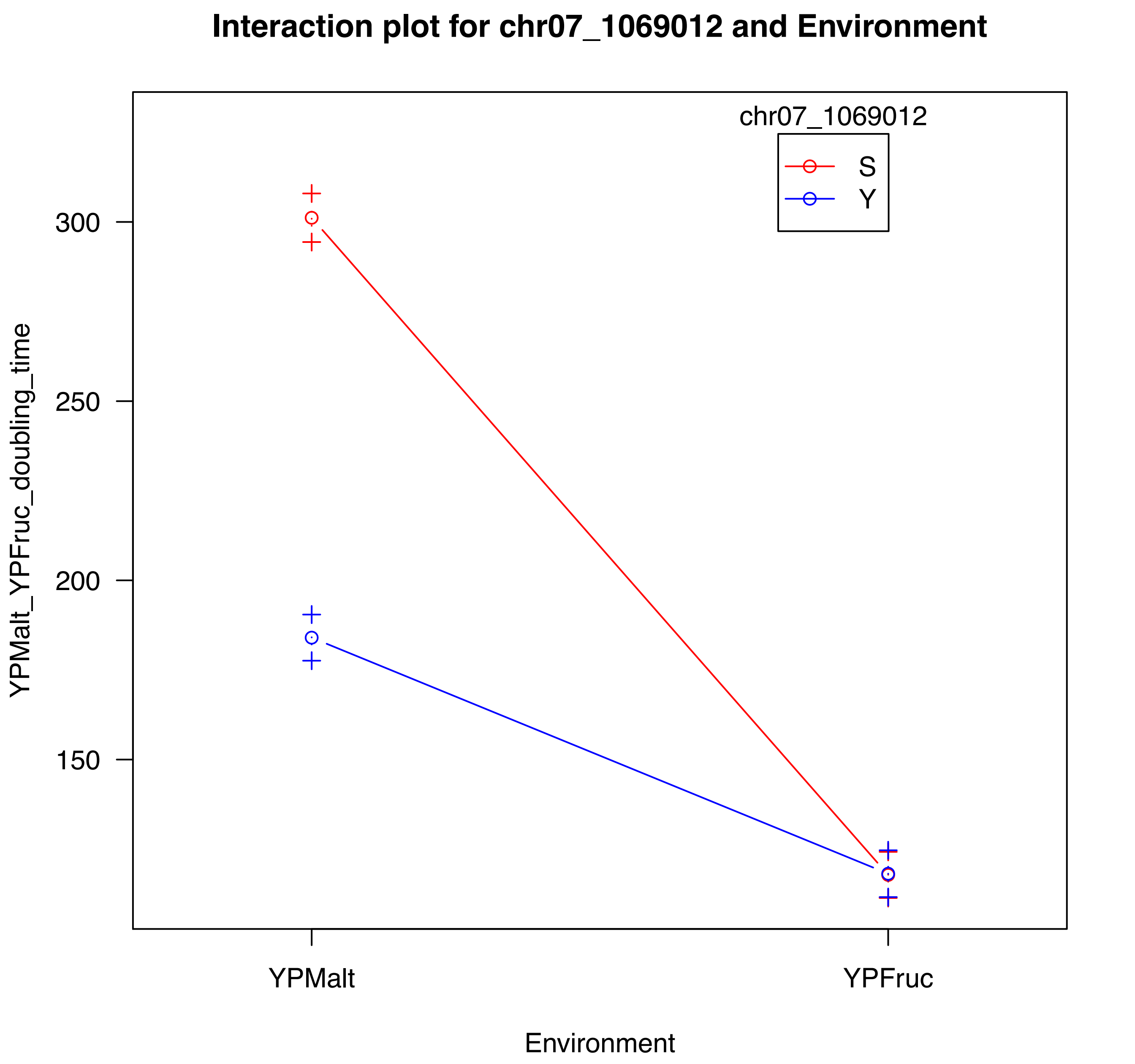
Interaction plot for chr07_1069012 and Environment
chr07_1069012
S
Y
300
250
200
150
YPMalt_YPFruc_doubling_time
YPMalt
YPFruc
Environment

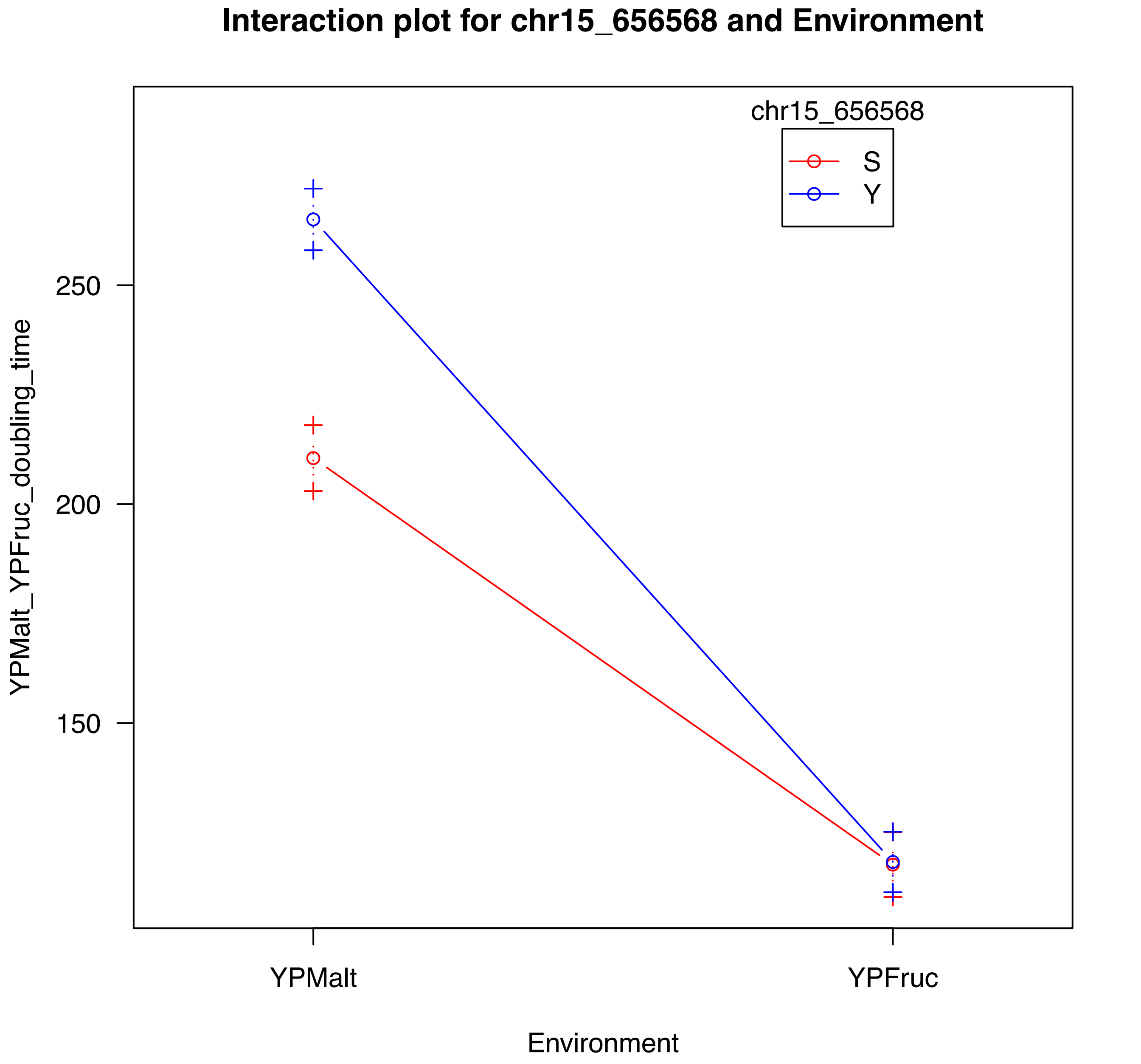

Interaction plot for chr15_656568 and Environment
chr15_656568
S
Y
YPMalt_YPFruc_doubling_time
250
200
150
YPMalt
YPFruc
Environment

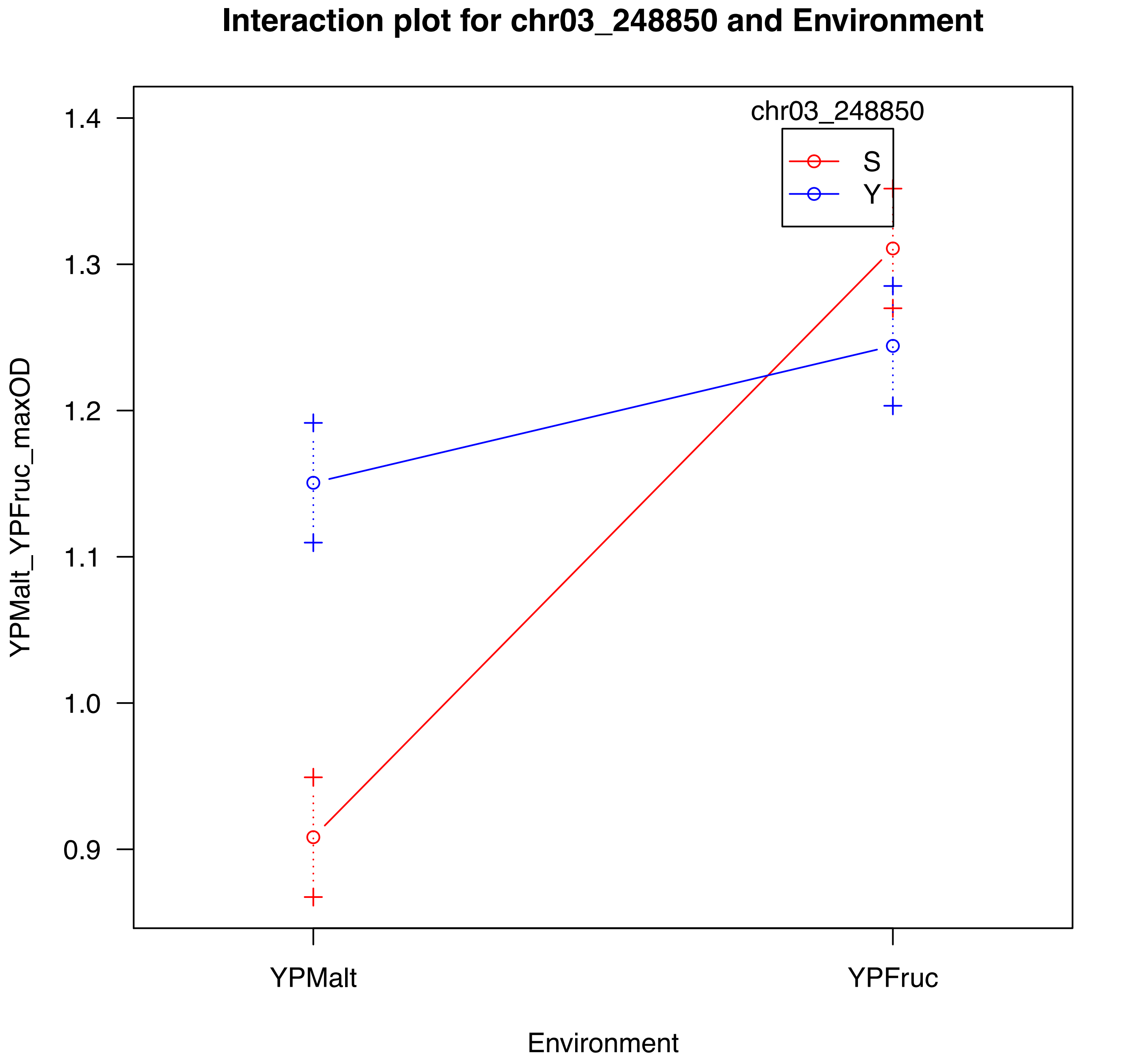

Interaction plot for chr03_248850 and Environment
chr03_248850
S
Y
YPMalt_YPFruc_maxOD
1.4
1.3
1.2
1.1
1.0
0.9
YPMalt
YPFruc
Environment

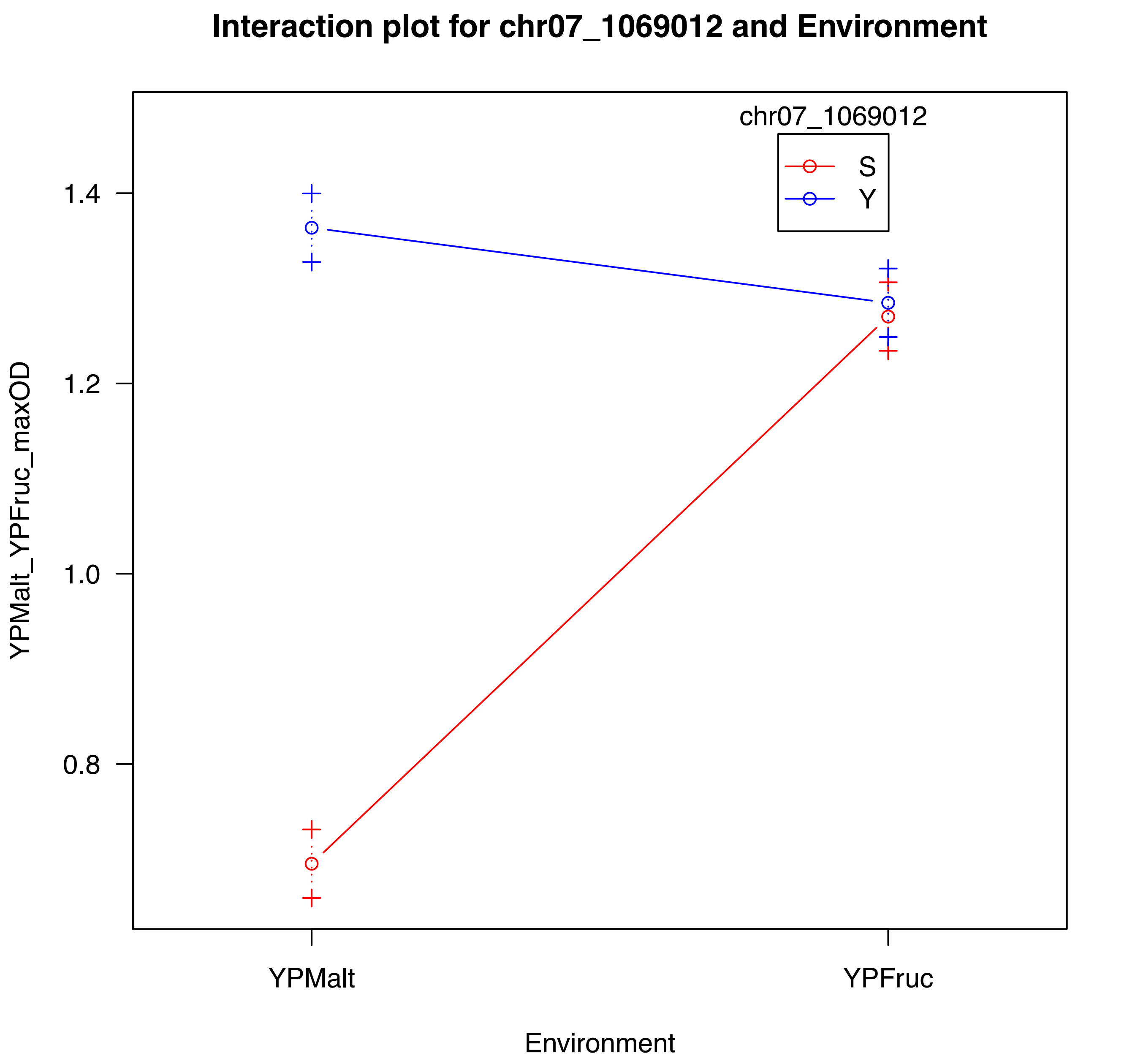

Interaction plot for chr07_1069012 and Environment
chr07_1069012
S
Y
1.4
1.2
1.0
0.8
YPMalt_YPFruc_maxOD
YPMalt
YPFruc
Environment

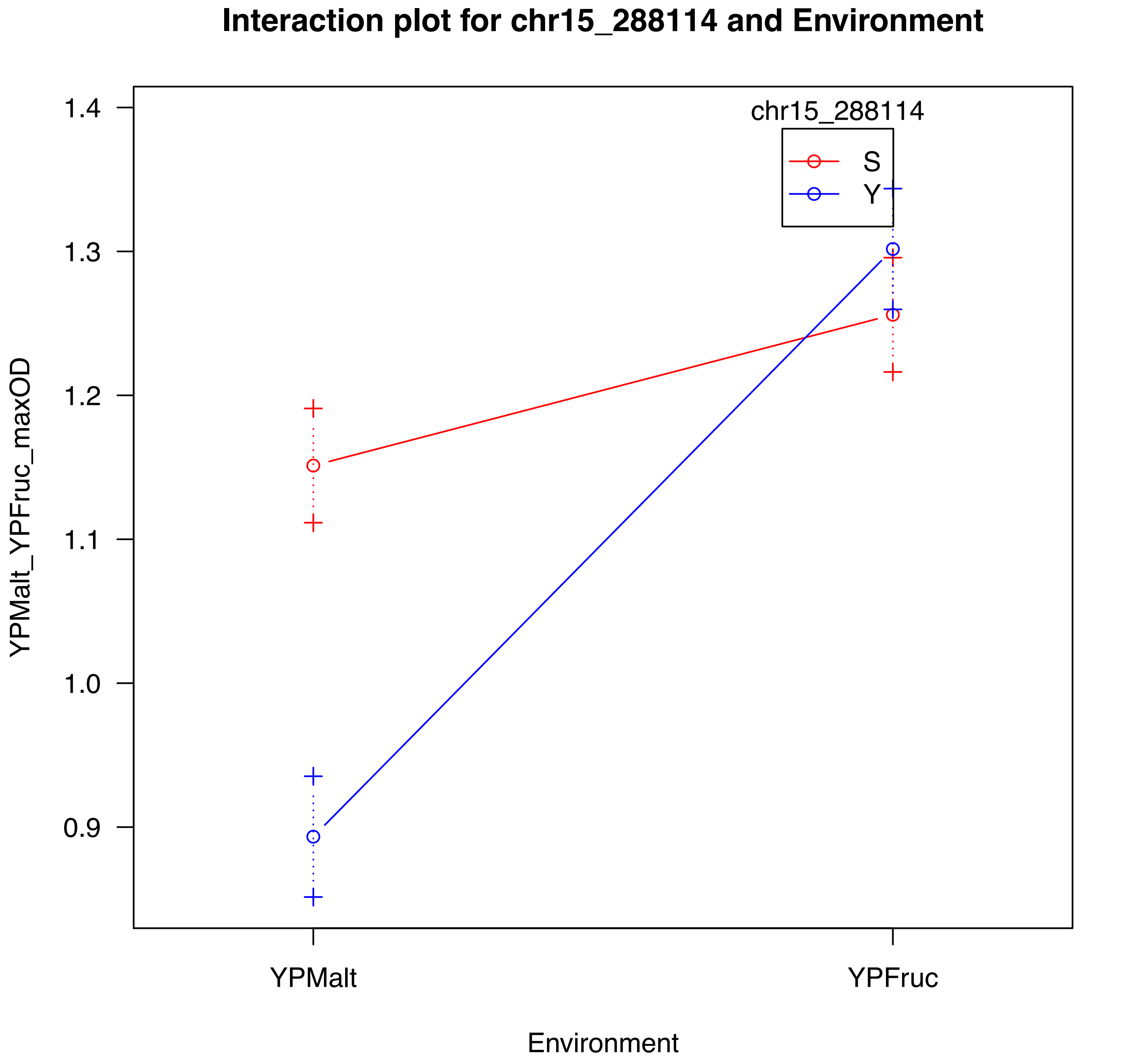

Interaction plot for chr15_288114 and Environment
chr15_288114
S
Y
1.4
1.3
1.2
1.1
1.0
0.9
YPMalt_YPFruc_maxOD
YPMalt
YPFruc
Environment

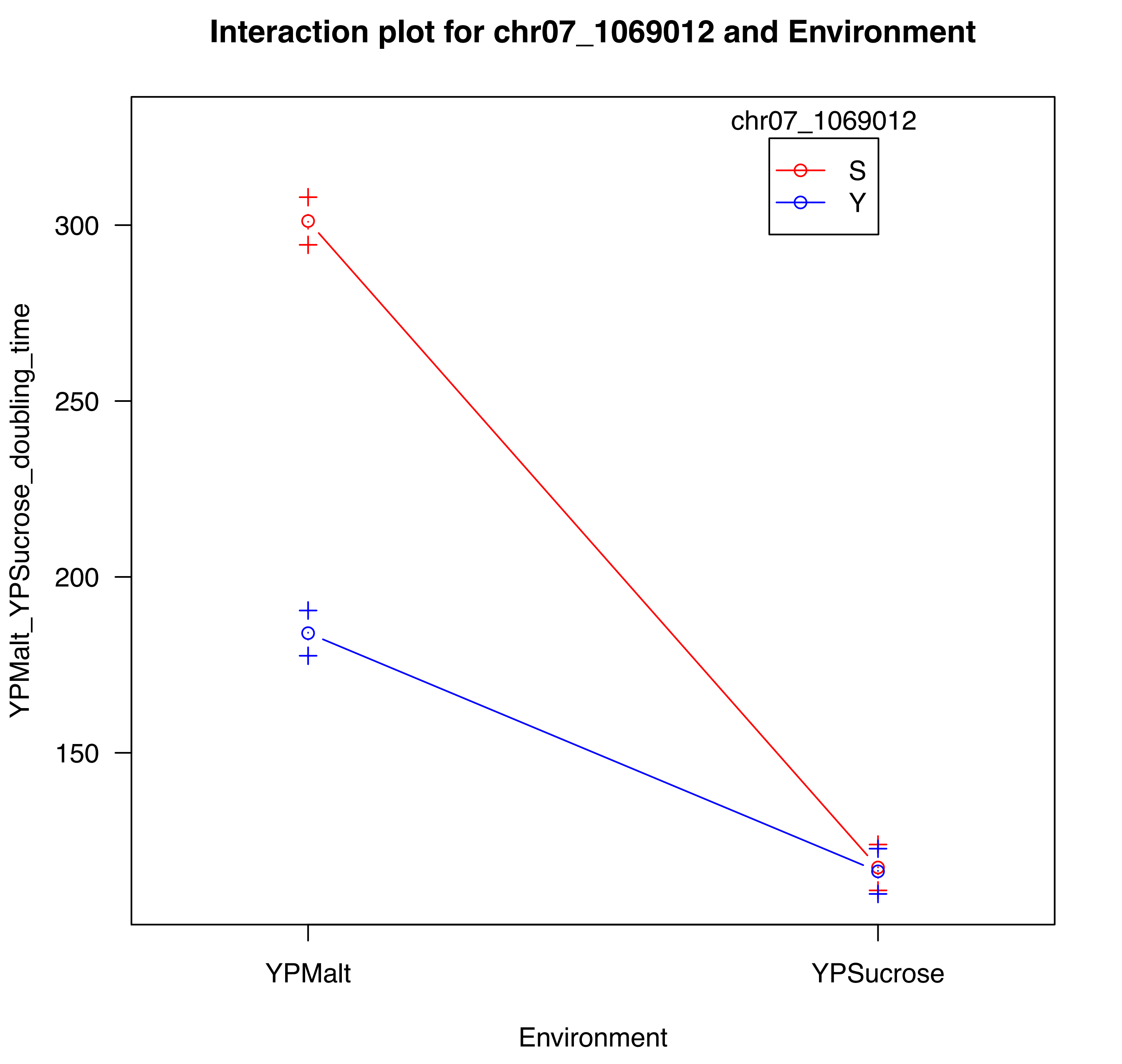

Interaction plot for chr07_1069012 and Environment
chr07_1069012
S
Y
300
250
200
150
YPMalt_YPSucrose_doubling_time
YPMalt
YPSucrose
Environment

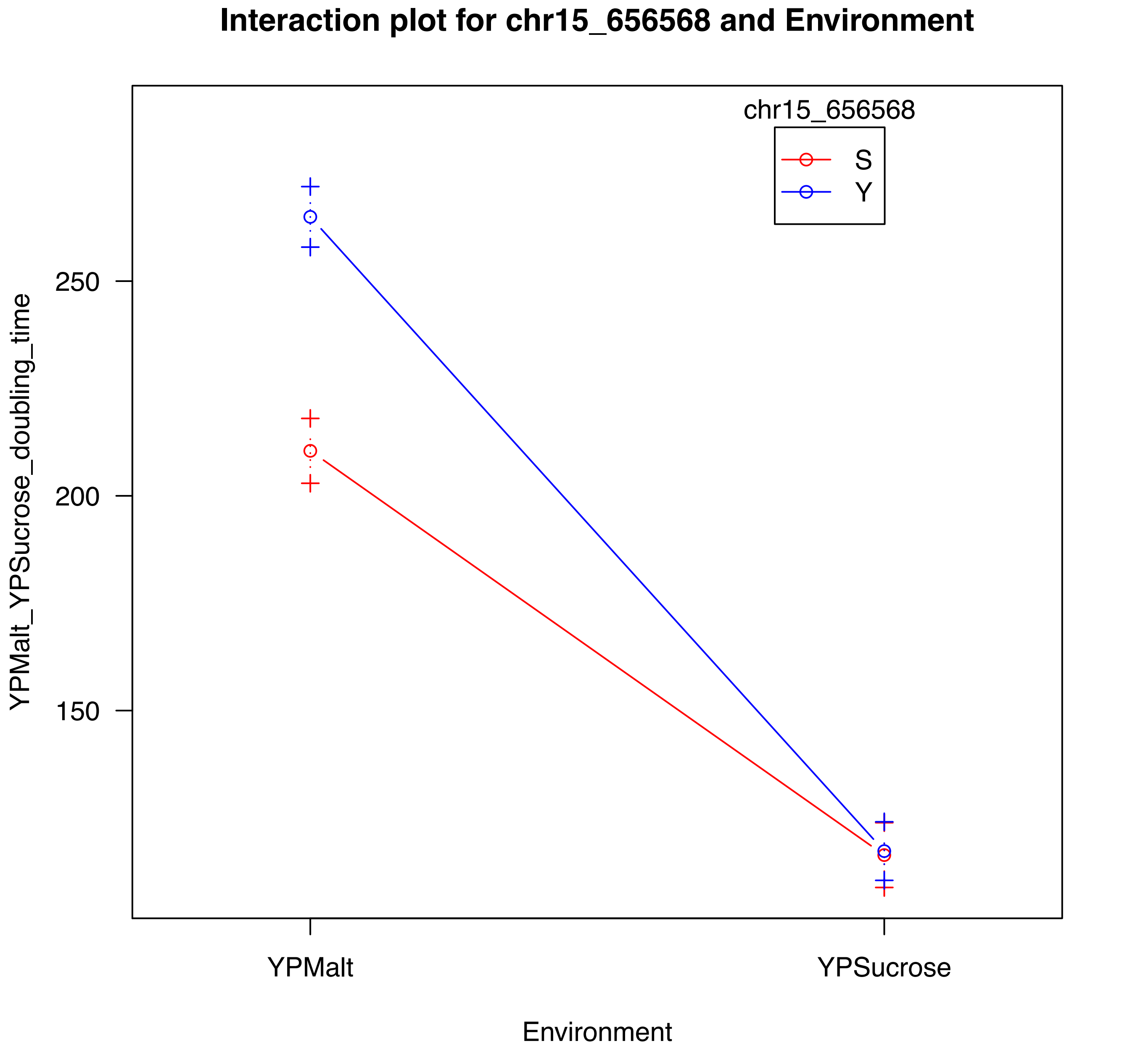

Interaction plot for chr15_656568 and Environment
chr15_656568
S
Y
250
200
150
YPMalt_YPSucrose_doubling_time
YPMalt
YPSucrose
Environment

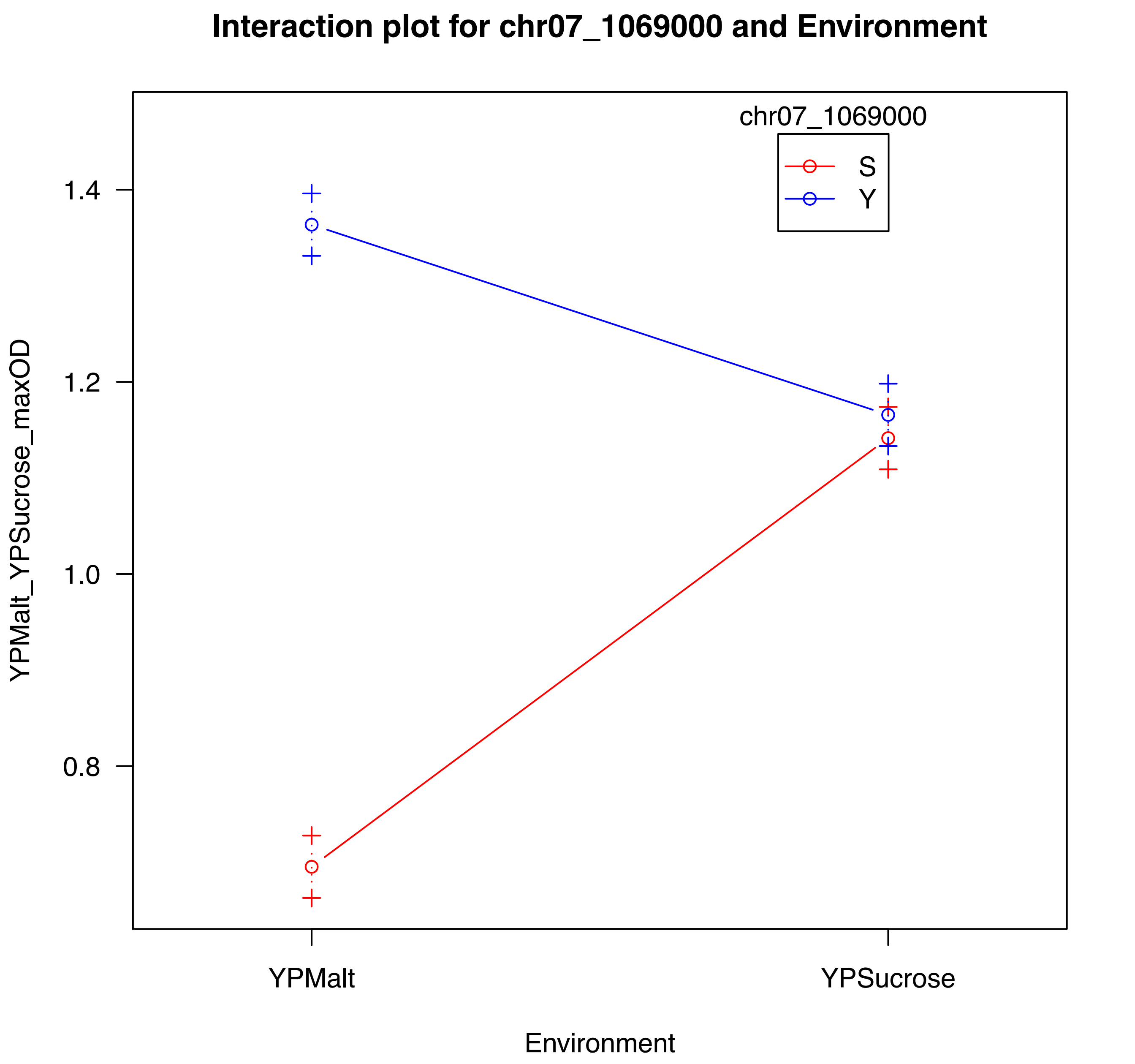
Interaction plot for chr07_1069000 and Environment
chr07_1069000
S
Y
1.4
1.2
1.0
0.8
YPMalt_YPSucrose_maxOD
YPMalt
YPSucrose
Environment

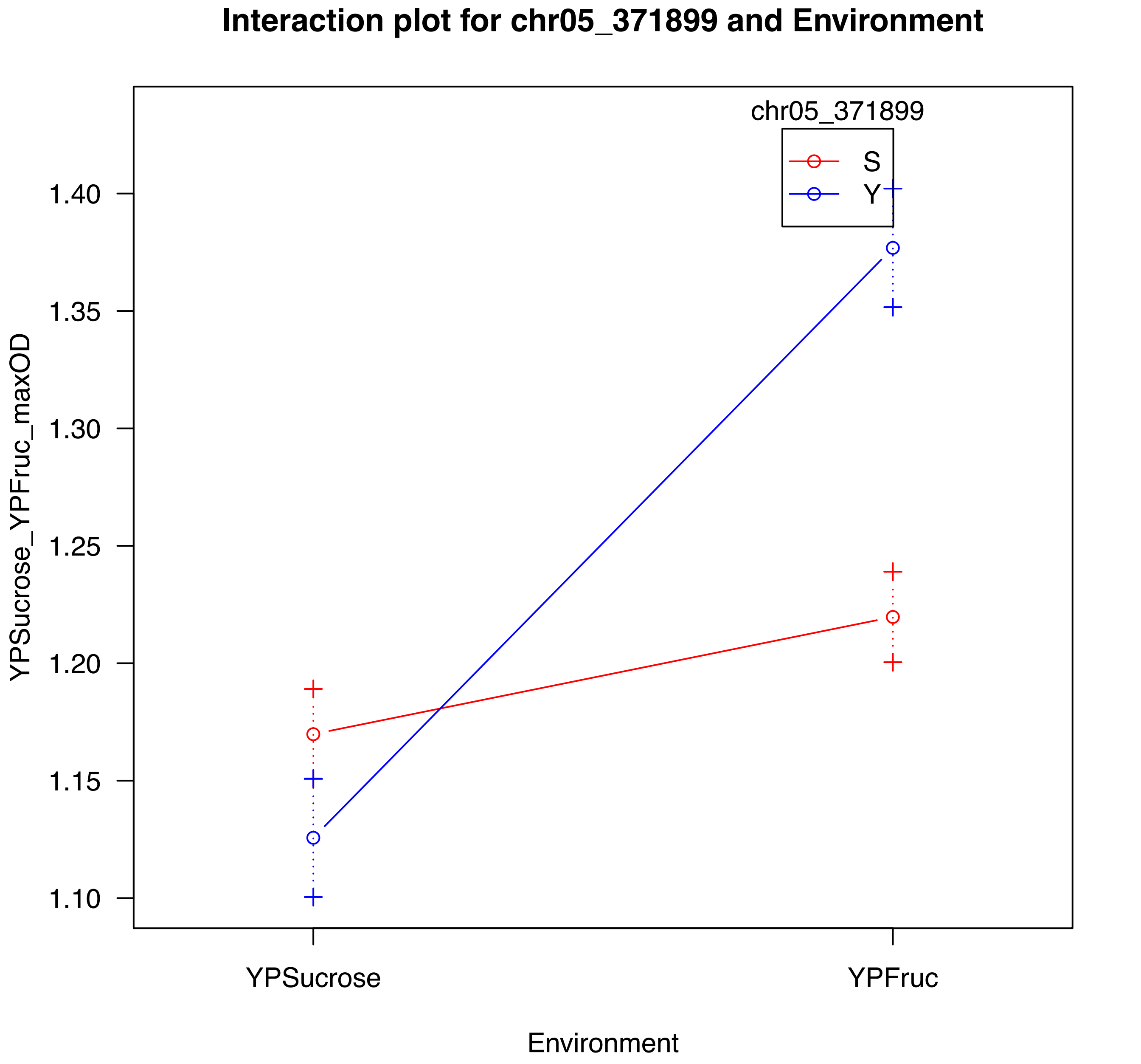

Interaction plot for chr05_371899 and Environment
chr05_371899
S
Y
1.40
1.35
1.30
1.25
1.20
1.15
1.10
YPSucrose_YPFruc_maxOD
YPSucrose
YPFruc
Environment
