# Supplementary material for: Yeast Growth Plasticity Is Regulated by Environment-Specific Multi-QTL Interactions
Source: G3 (Bethesda). 2014 Jan 28;4(5):769–77. doi: 10.1534/g3.113.009142 (PMC4025475; doi:10.1534/g3.113.009142)
Supplement: Supporting Information [file supp_g3.113.009142_FigureS3.pdf]

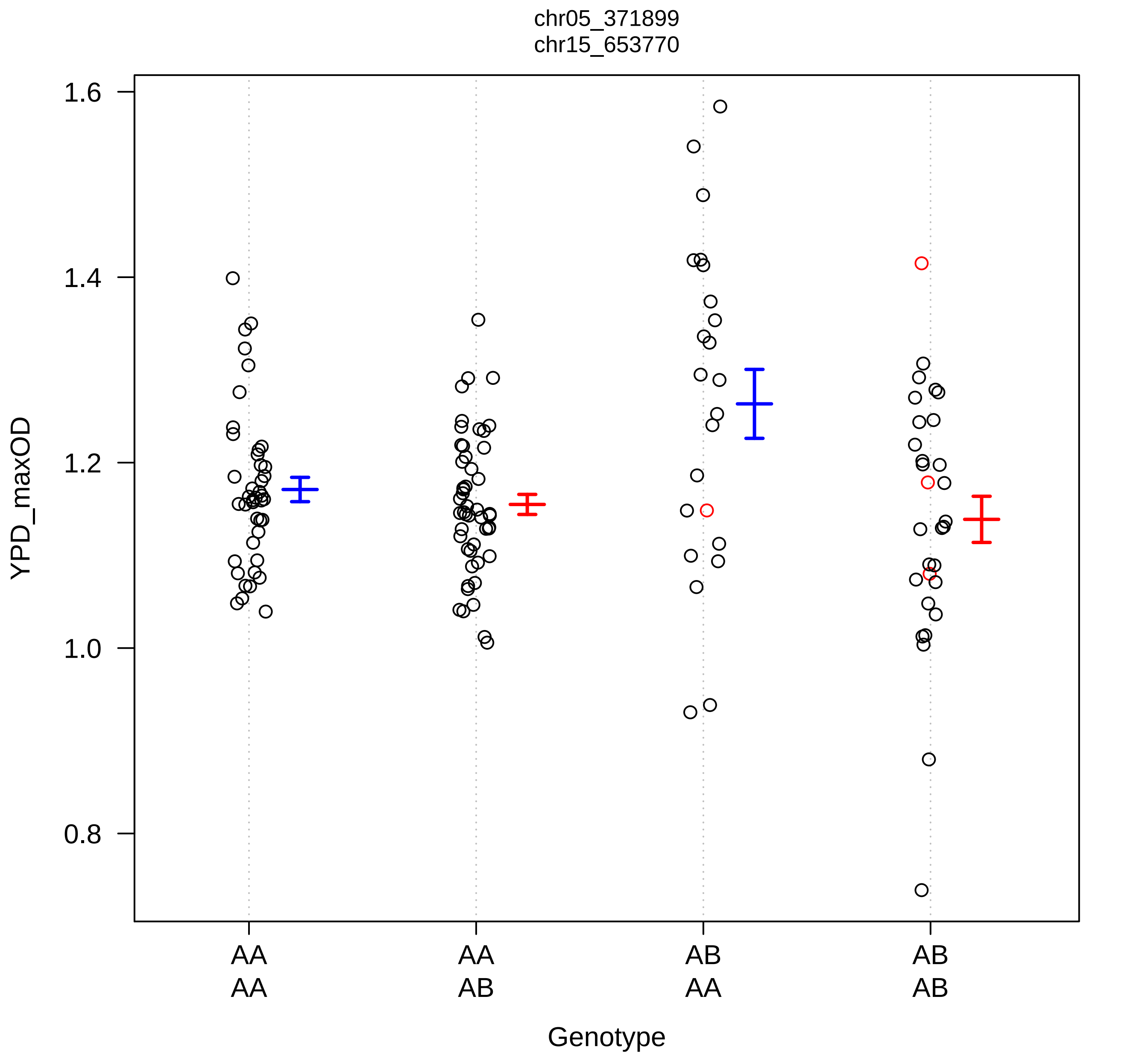
chr05_371899
chr15_653770
YPD_maxOD
1.6
1.4
1.2
1.0
0.8
AA
AA
AA
AB
AB
AA
AB
AB
Genotype

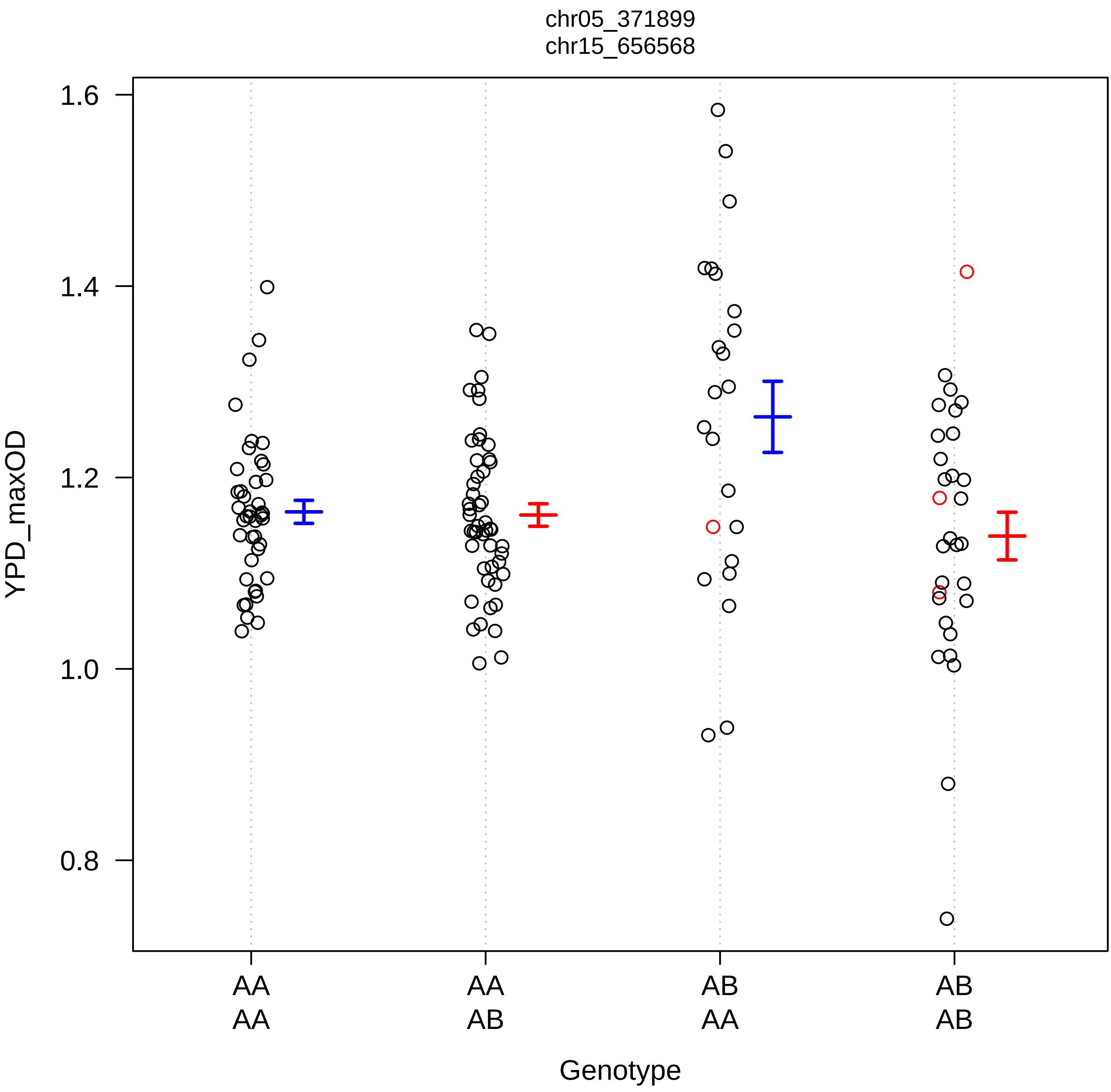
chr05_371899
chr15_656568
YPD_maxOD
1.6
1.4
1.2
1.0
0.8
AA
AA
AA
AB
AB
AA
AB
AB
Genotype

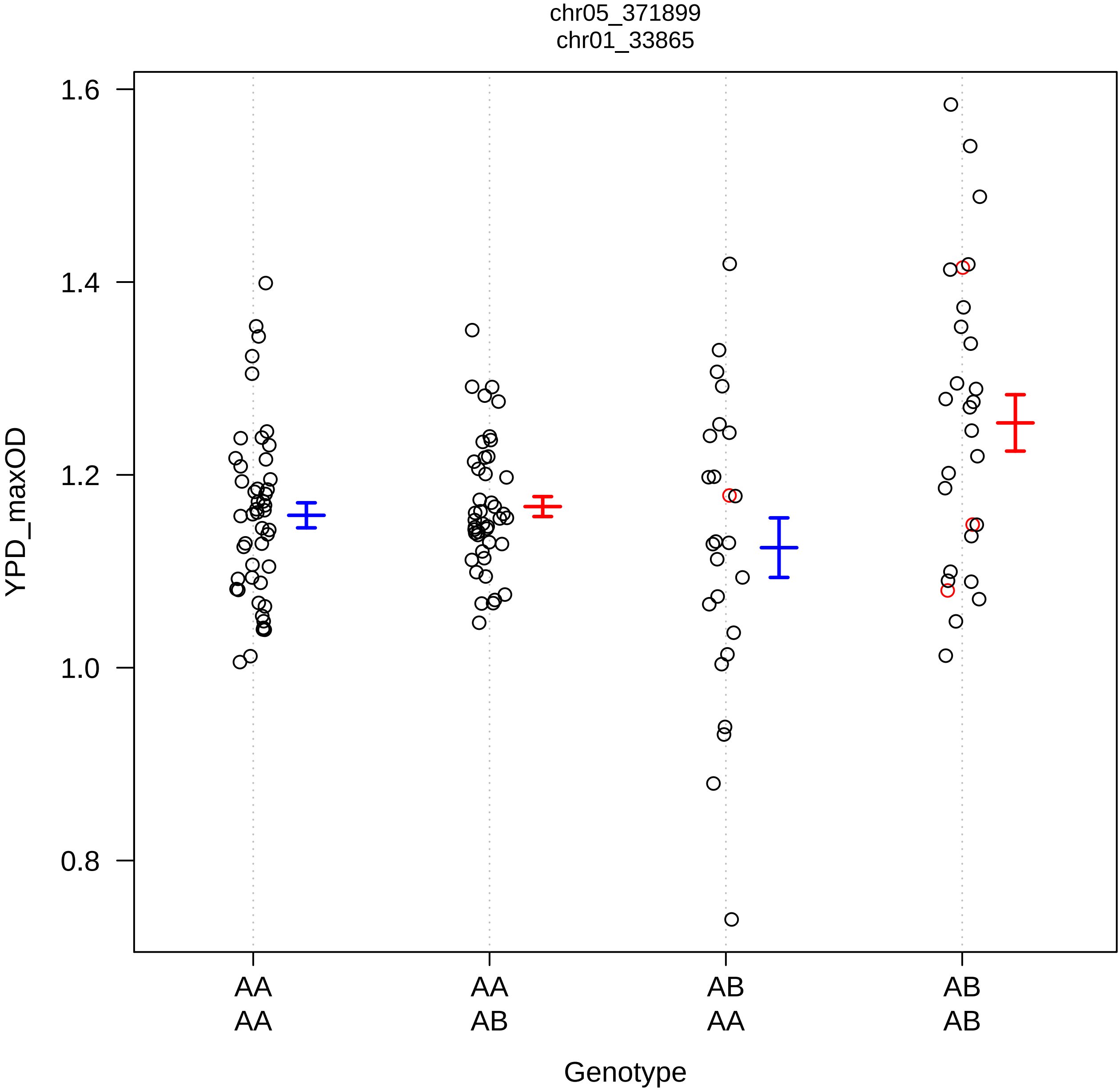
chr05_371899
chr01_33865
YPD_maxOD
1.6
1.4
1.2
1.0
0.8
AA AA
AA AB
AB AA
AB AB
Genotype

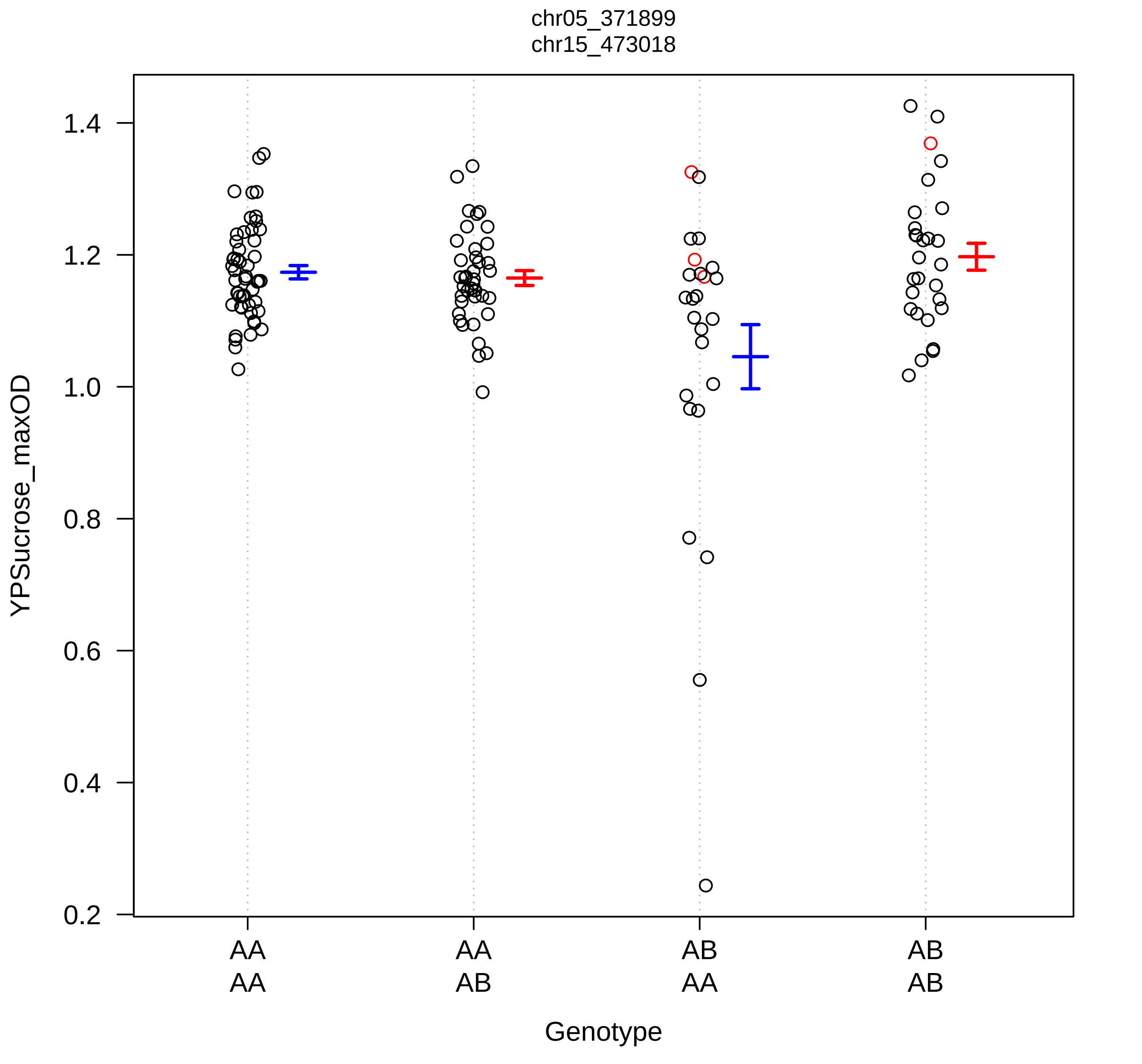
chr05_371899
chr15_473018
YPSucrose_maxOD
1.4
1.2
1.0
0.8
0.6
0.4
0.2
AA
AA
AA
AB
AB
AA
AB
AB
Genotype

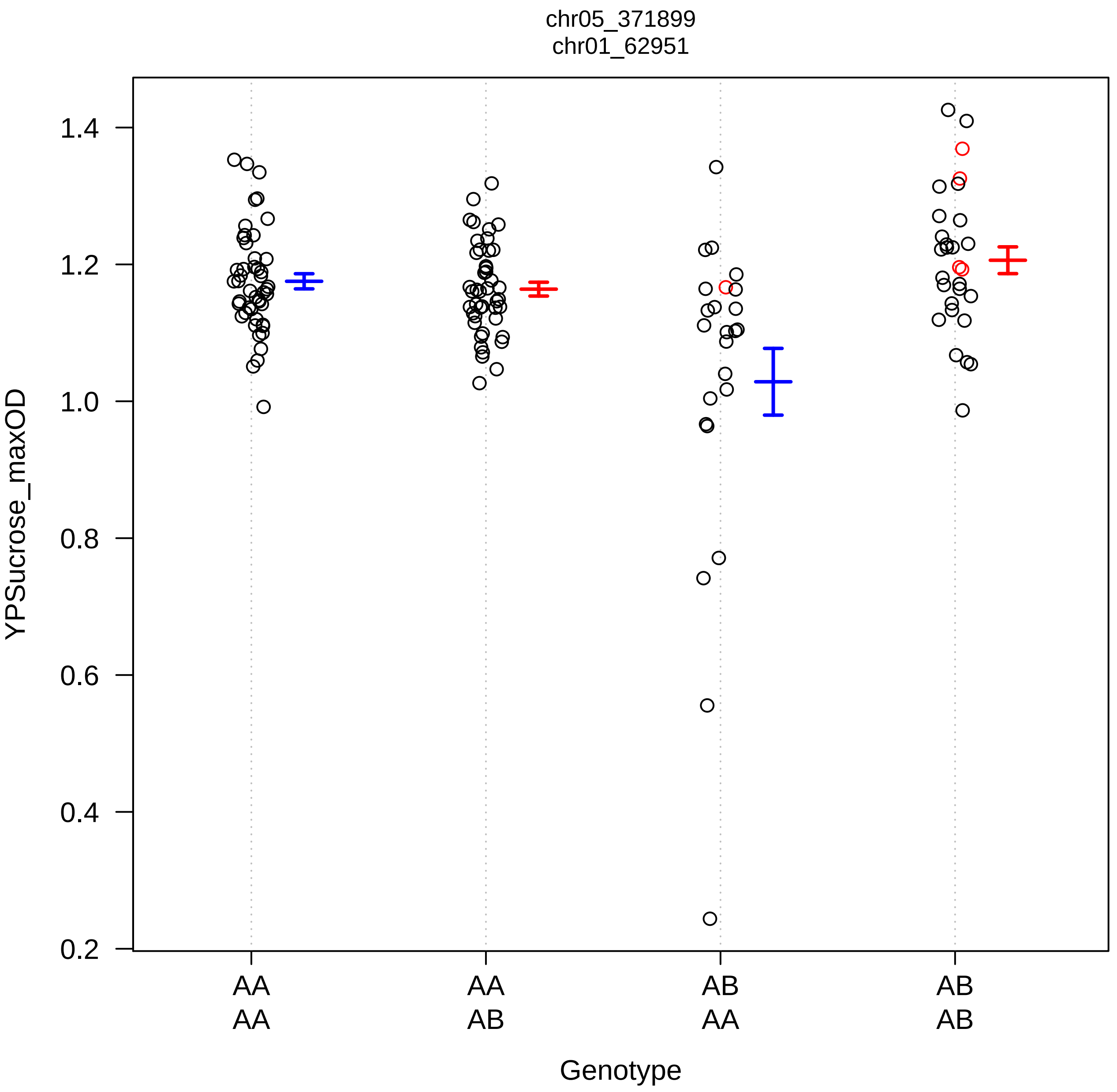
chr05_371899
chr01_62951
YPSucrose_maxOD
1.4
1.2
1.0
0.8
0.6
0.4
0.2
AA
AA
AA
AB
AB
AA
AB
AB
Genotype

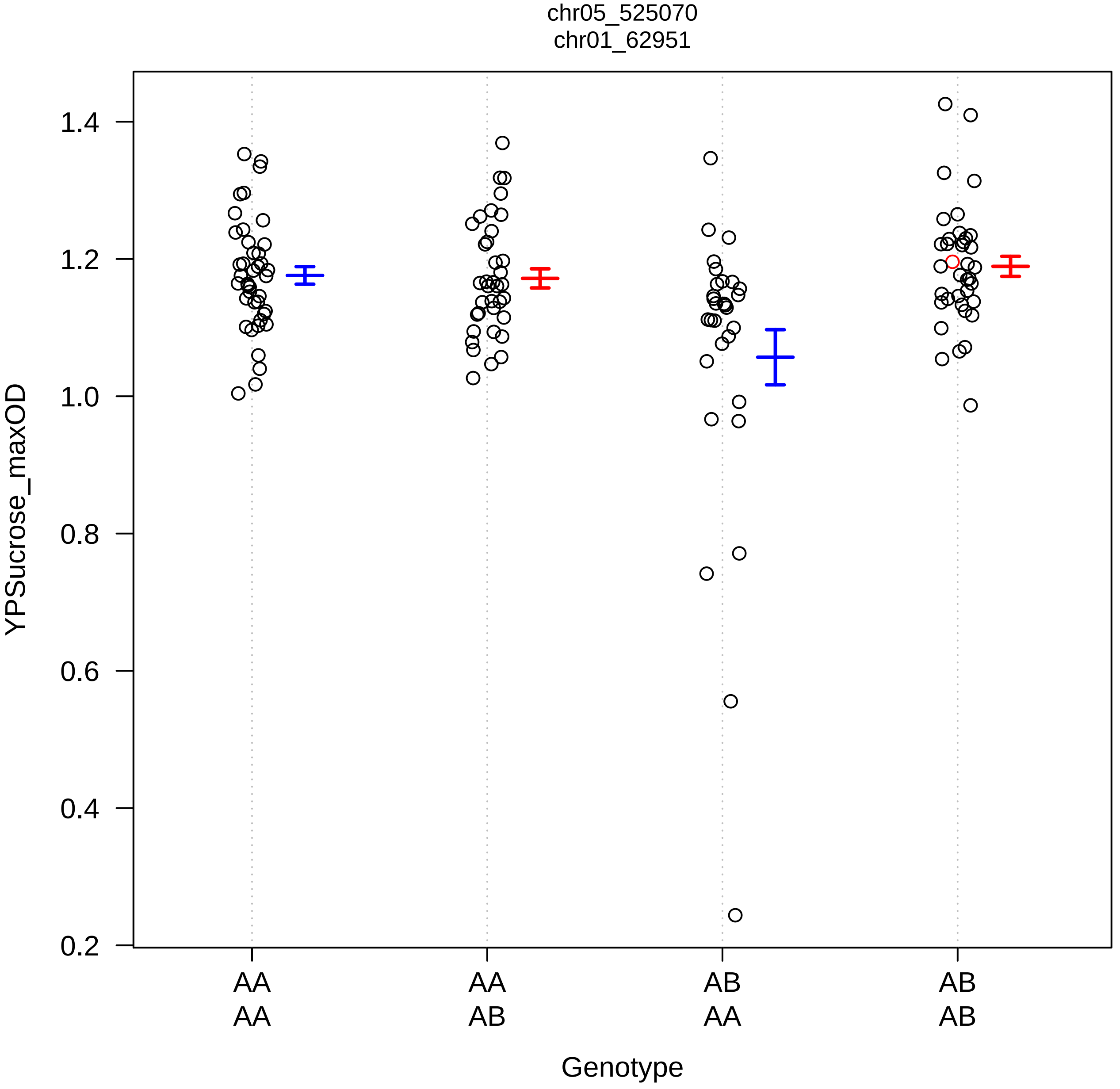

chr05_525070
chr01_62951
YPSucrose_maxOD
1.4
1.2
1.0
0.8
0.6
0.4
0.2
AA
AA
AA
AB
AB
AA
AB
AB
Genotype

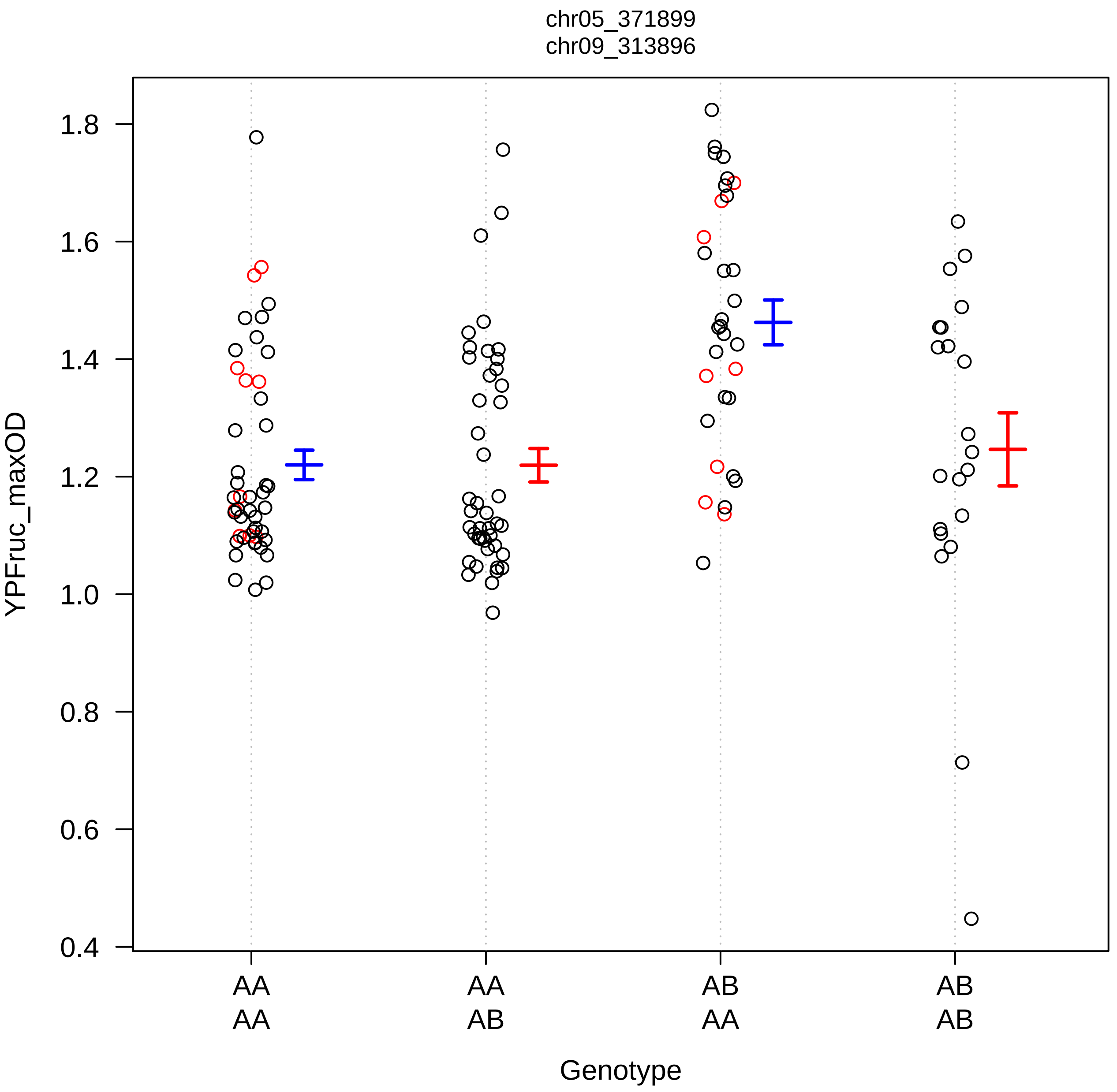
chr05_371899
chr09_313896
YPFruc_maxOD
1.8
1.6
1.4
1.2
1.0
0.8
0.6
0.4
AA
AA
AA
AB
AB
AA
AB
AB
Genotype

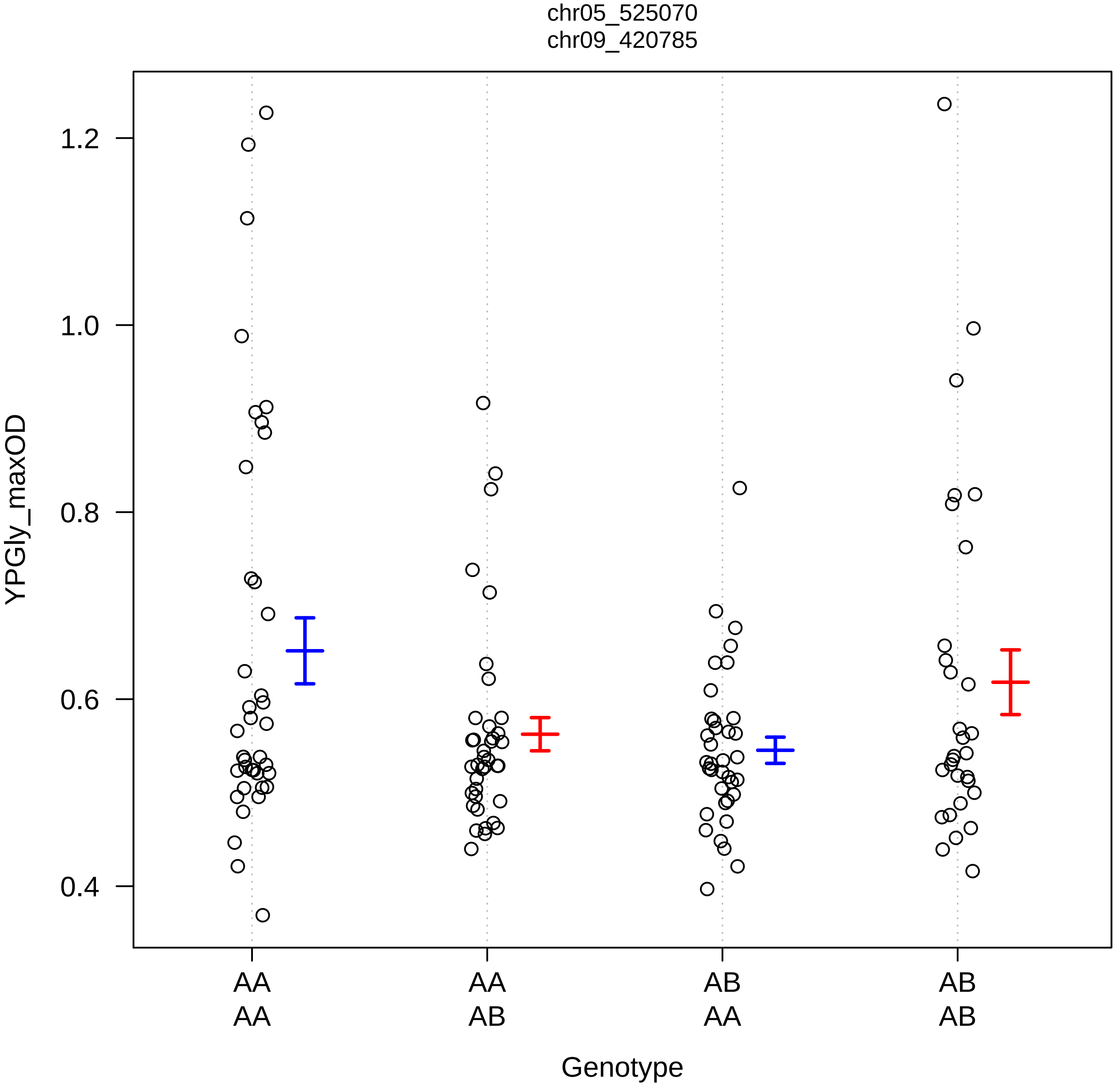
chr05_525070
chr09_420785
YPGly_maxOD
1.2
1.0
0.8
0.6
0.4
AA
AA
AA
AB
AB
AA
AB
AB
Genotype

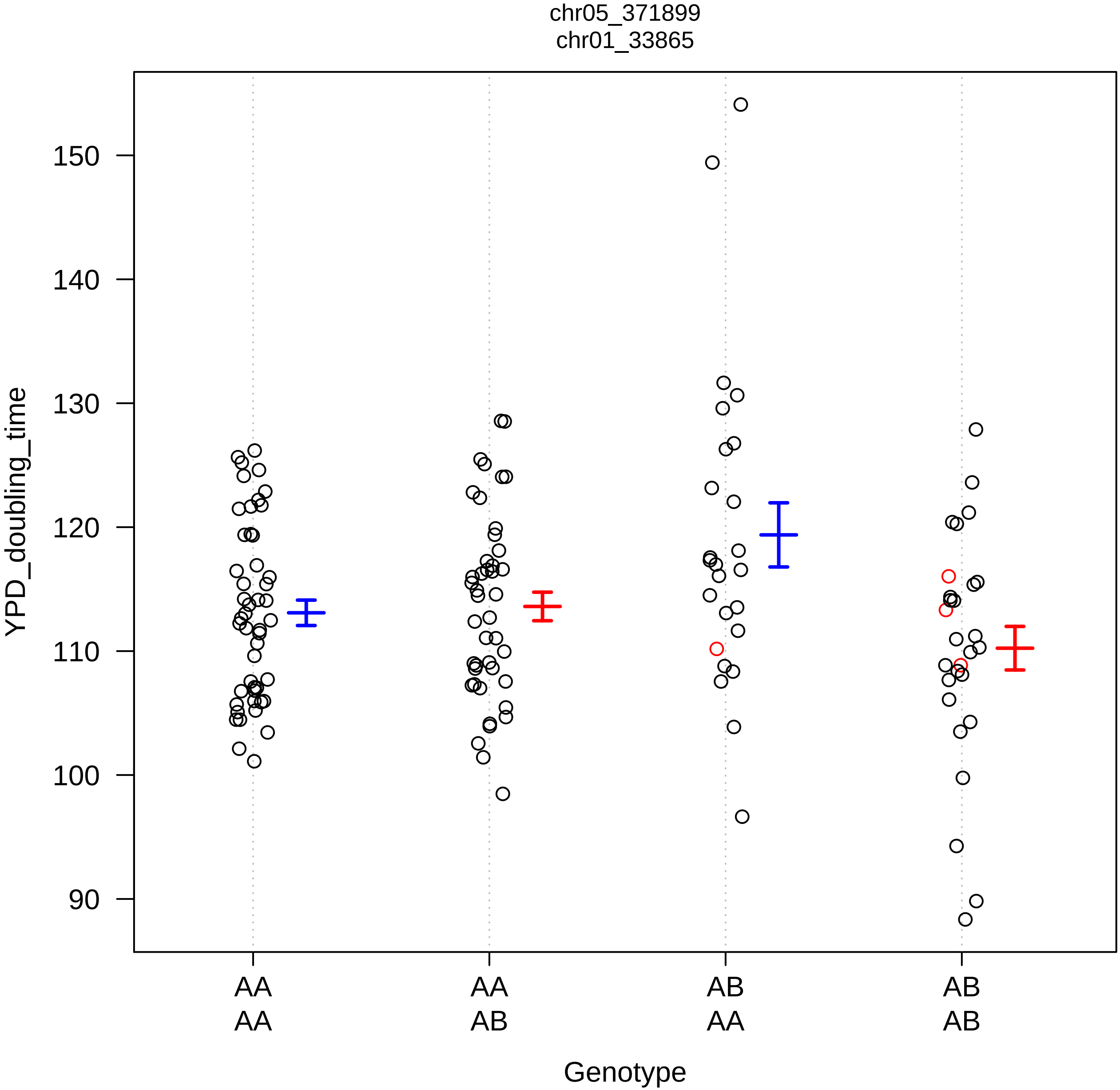
chr05_371899
chr01_33865
YPD_doubling_time
150
140
130
120
110
100
90
AA
AA
AA
AB
AB
AA
AB
AB
Genotype

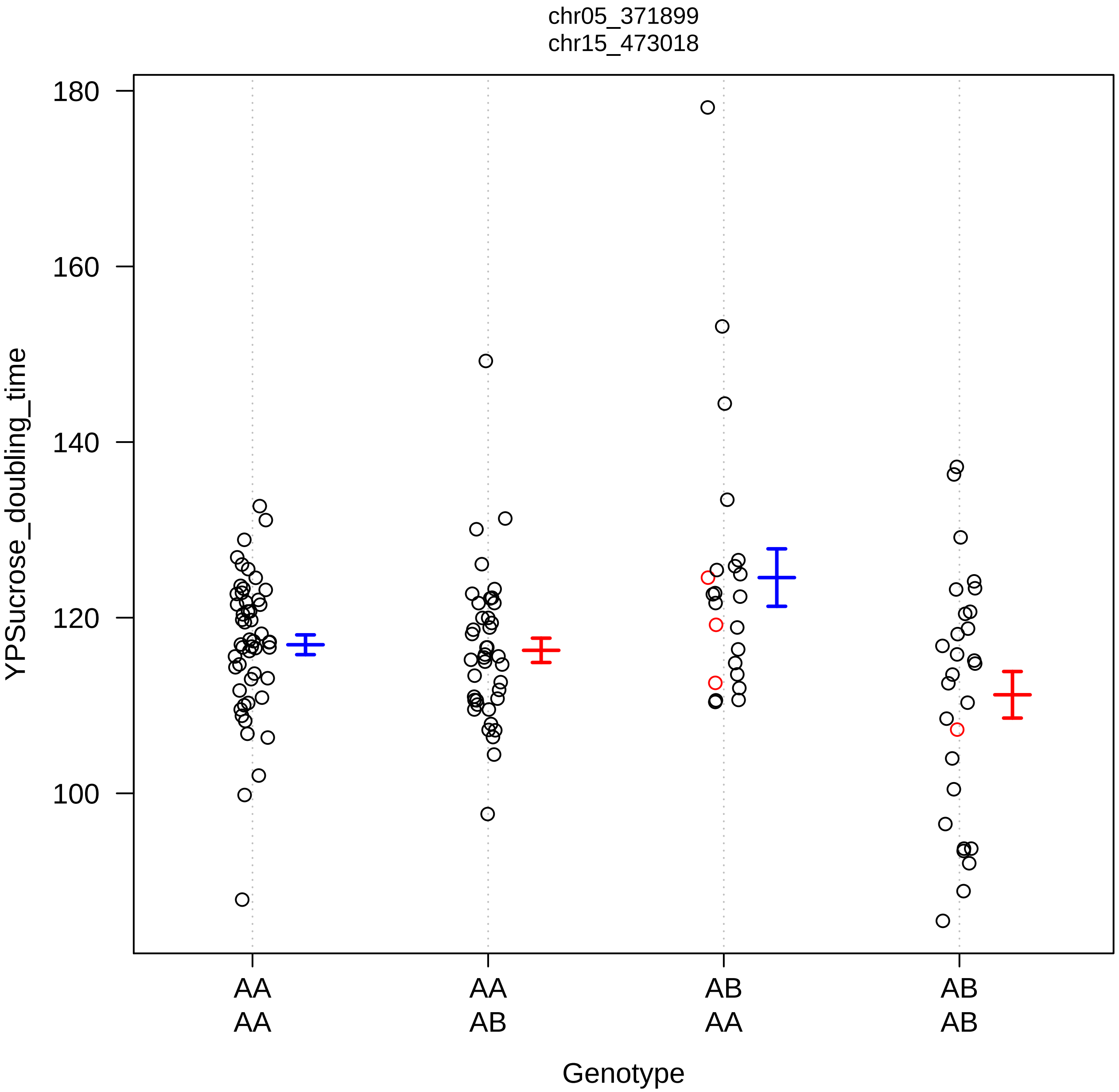
chr05_371899
chr15_473018
YPSucrose_doubling_time
180
160
140
120
100
AA AA
AA AB
AB AA
AB AB
Genotype

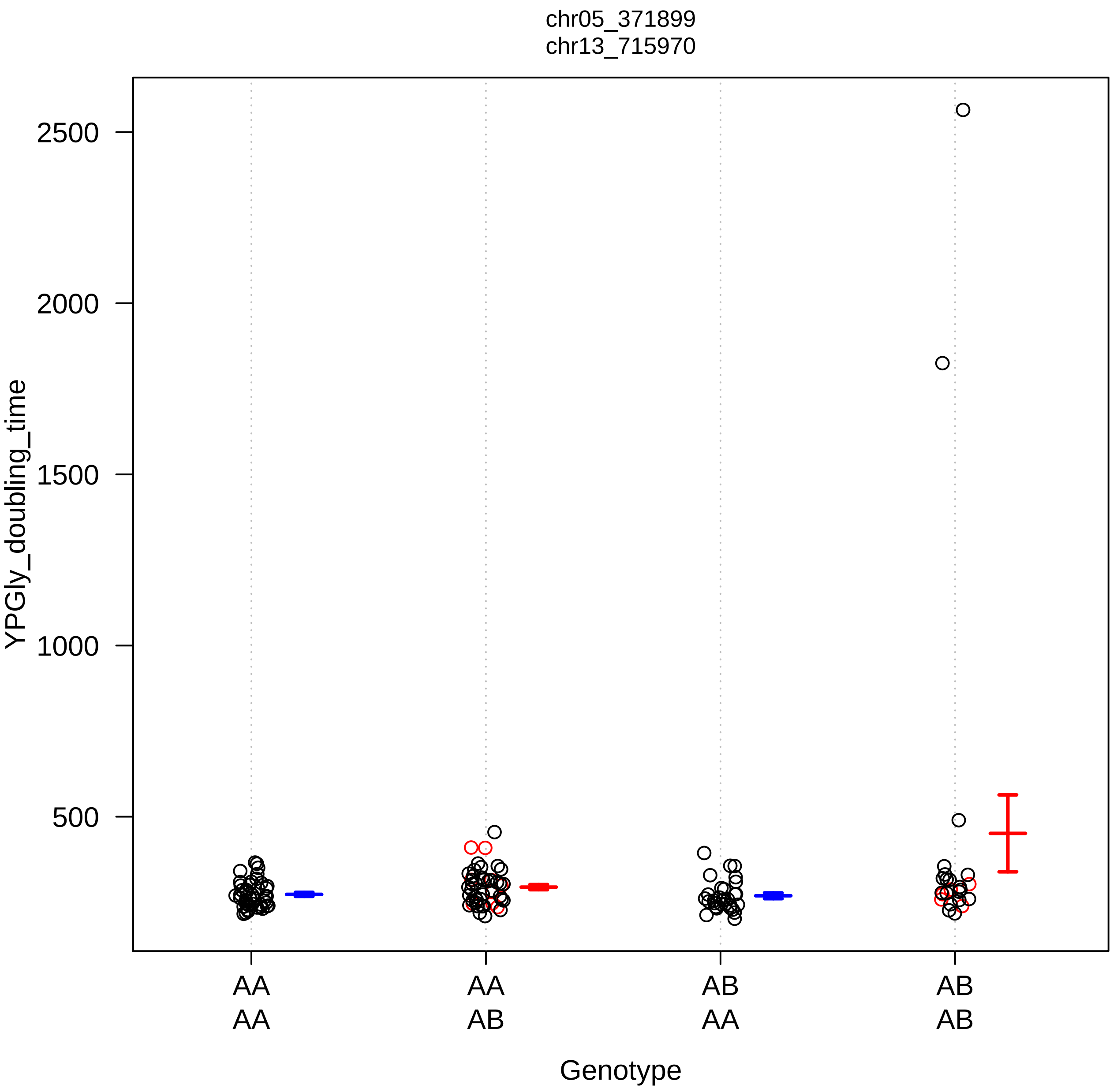
chr05_371899
chr13_715970
YPGly_doubling_time
2500
2000
1500
1000
500
AA
AA
AA
AB
AB
AA
AB
AB
Genotype

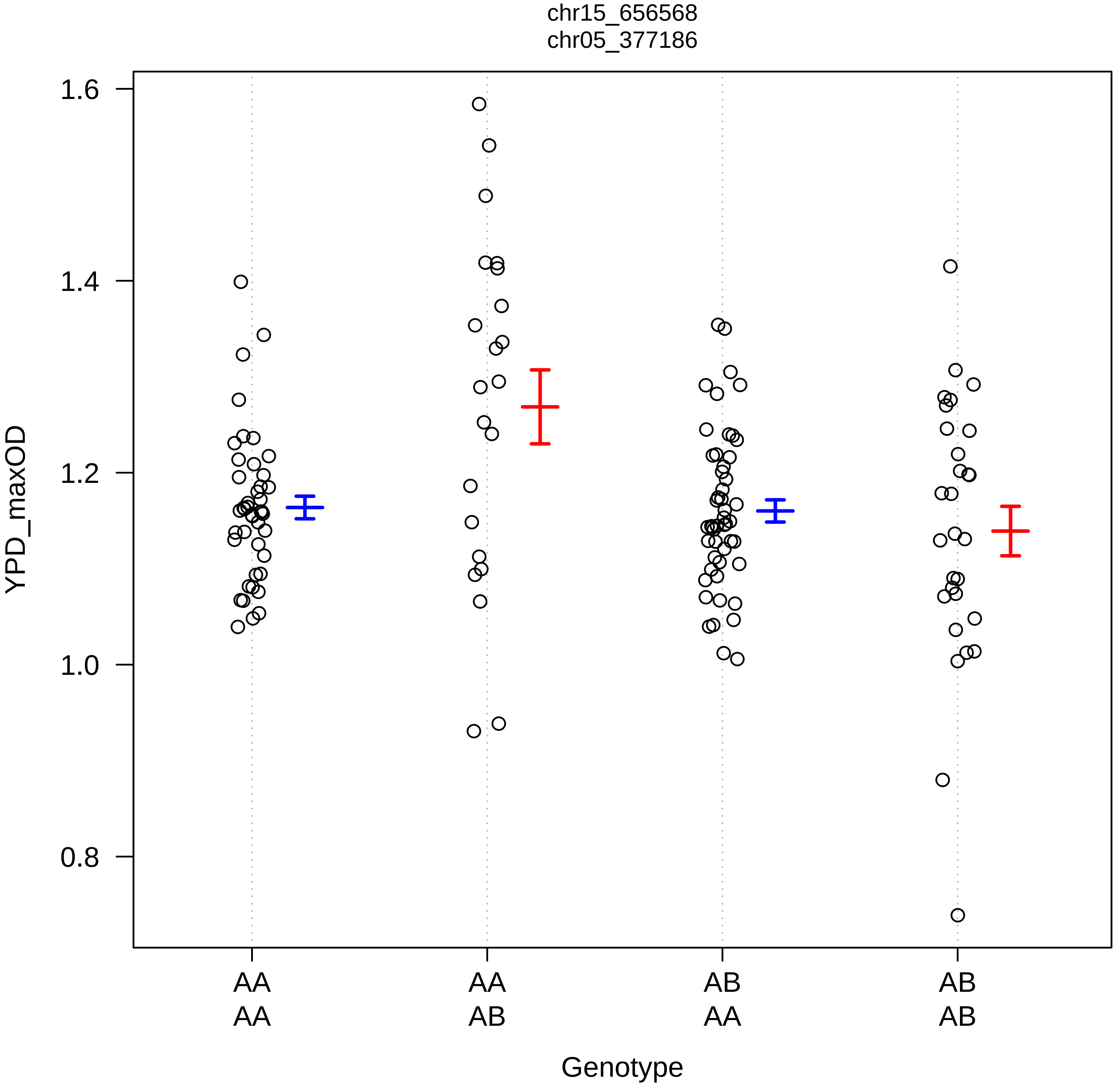

chr15_656568
chr05_377186
YPD_maxOD
1.6
1.4
1.2
1.0
0.8
AA
AA
AA
AB
AB
AA
AB
AB
Genotype

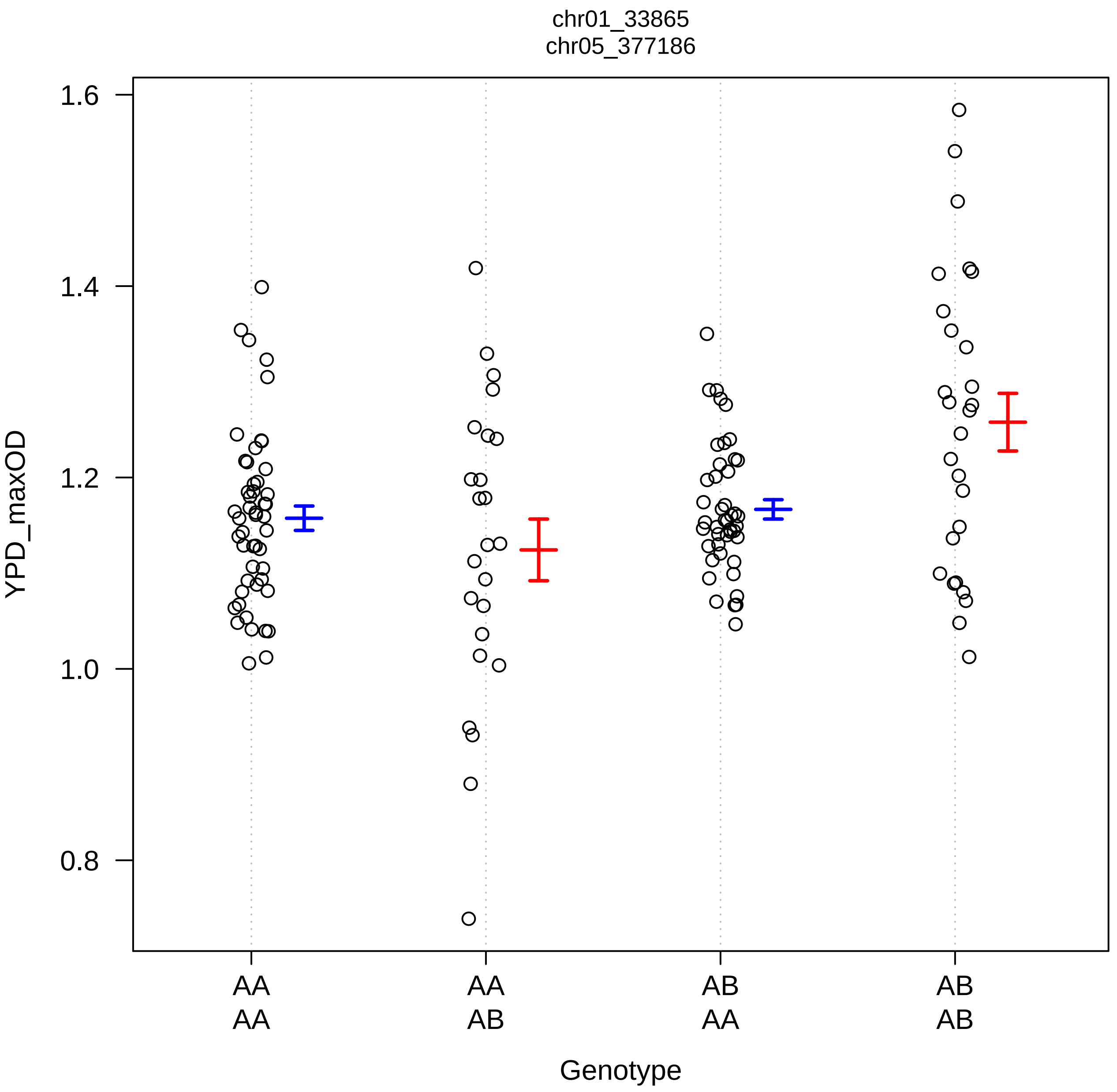
chr01_33865
chr05_377186
YPD_maxOD
1.6
1.4
1.2
1.0
0.8
AA
AA
AA
AB
AB
AA
AB
AB
Genotype

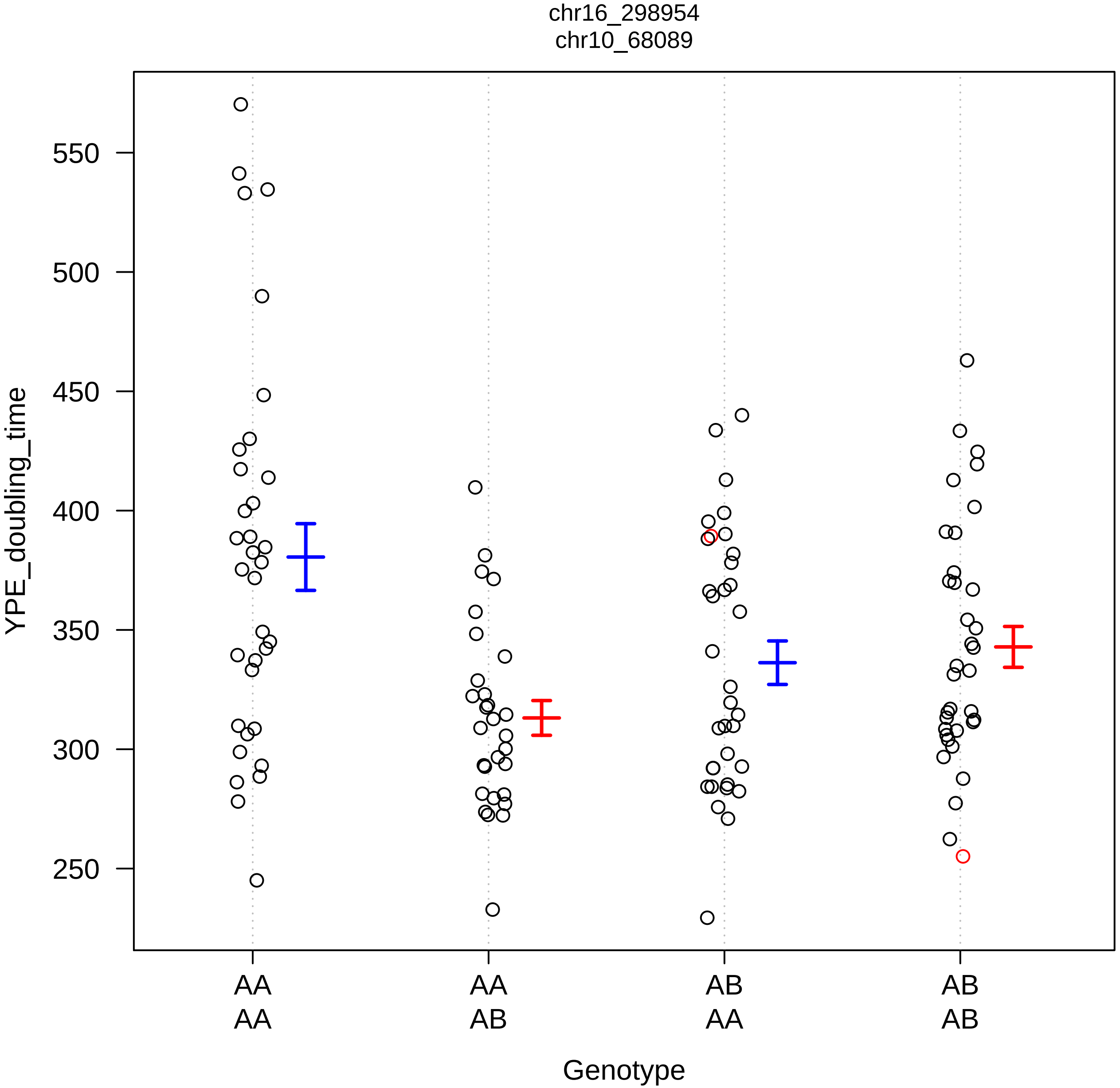
chr16_298954
chr10_68089
YPE_doubling_time
550
500
450
400
350
300
250
AA
AA
AA
AB
AB
AA
AB
AB
Genotype

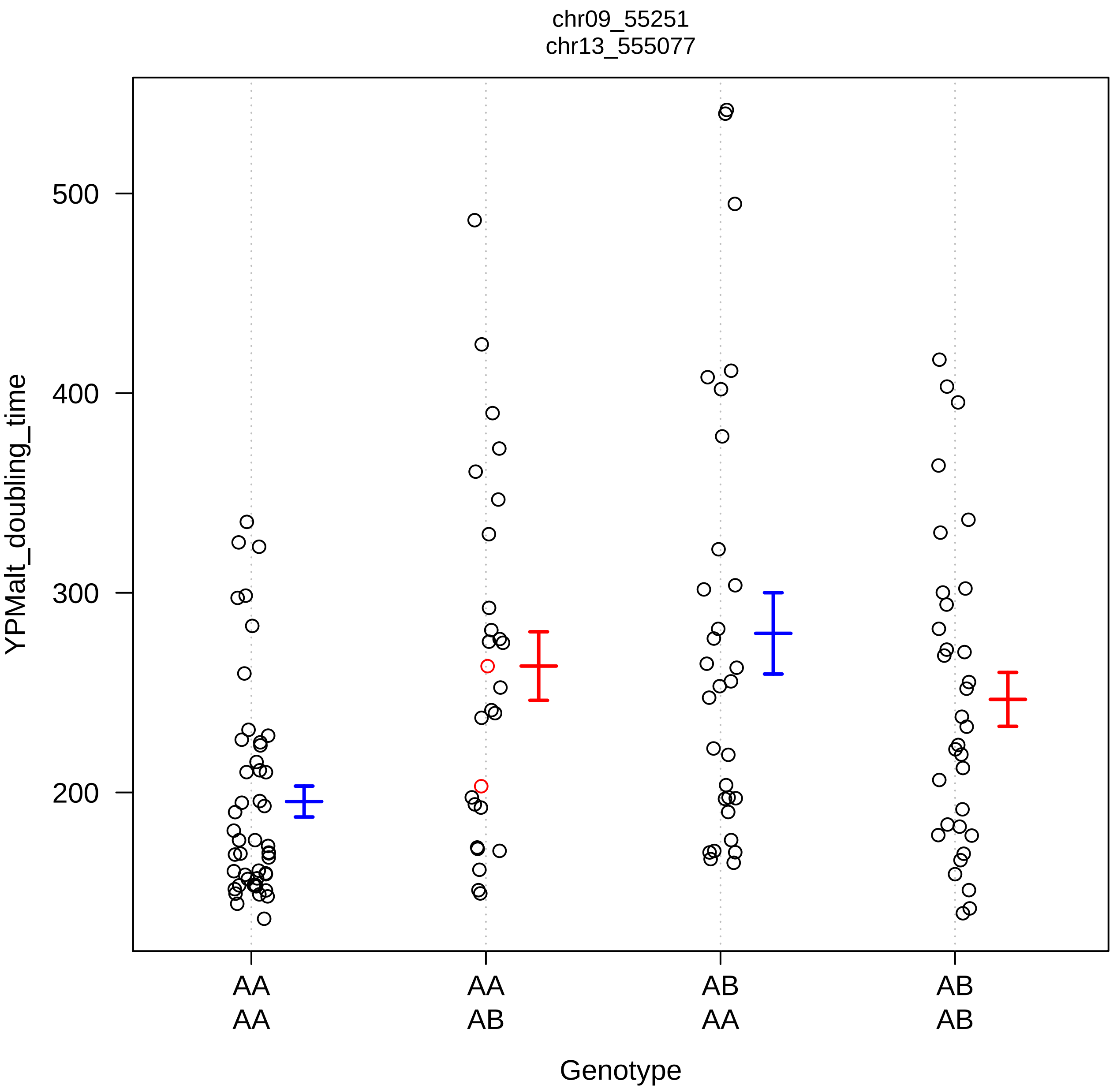
chr09_55251
chr13_555077
YPMalt_doubling_time
500
400
300
200
AA AA
AA AB
AB AA
AB AB
Genotype

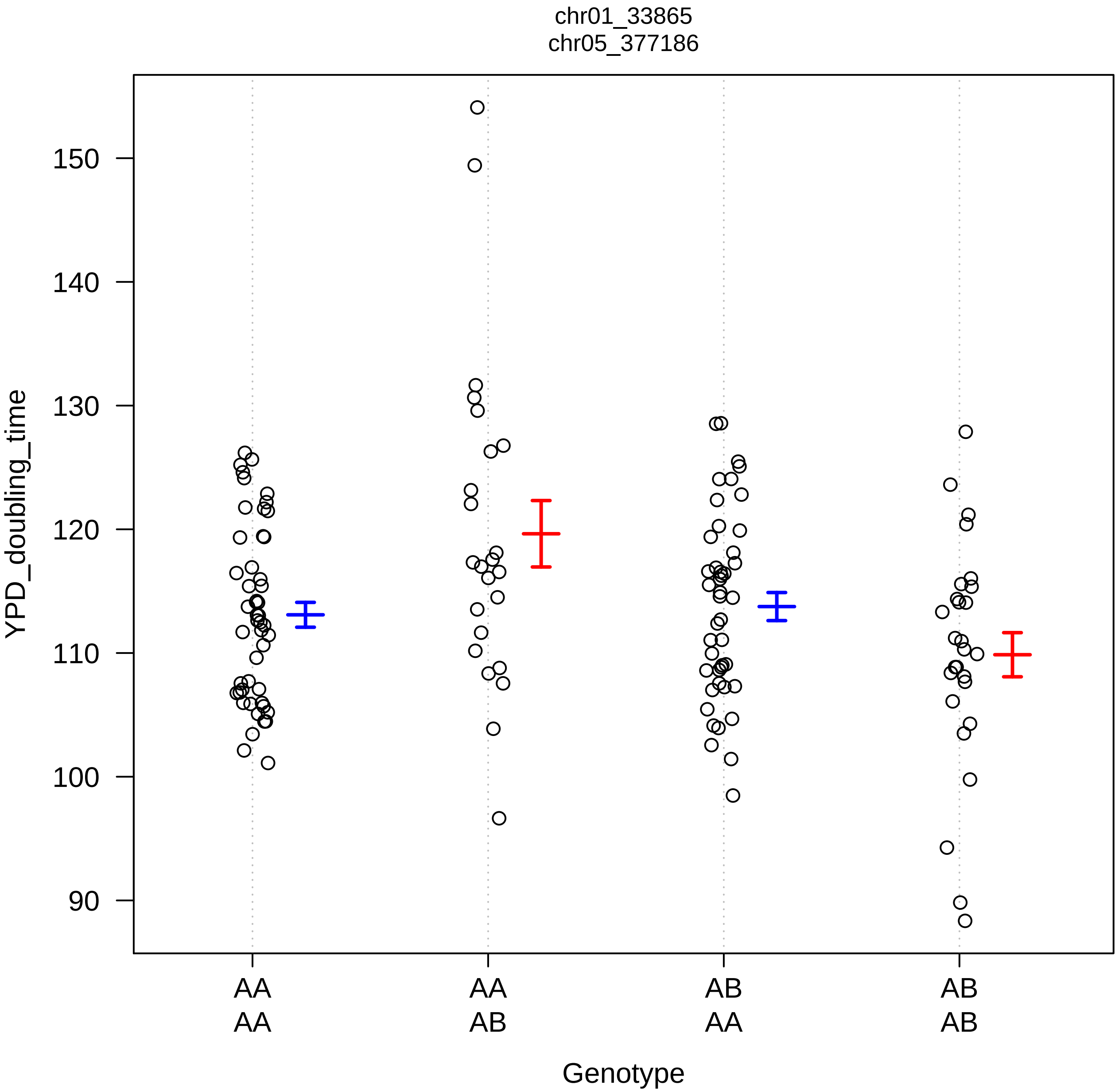
chr01_33865
chr05_377186
YPD_doubling_time
150
140
130
120
110
100
90
AA
AA
AA
AB
AB
AA
AB
AB
Genotype

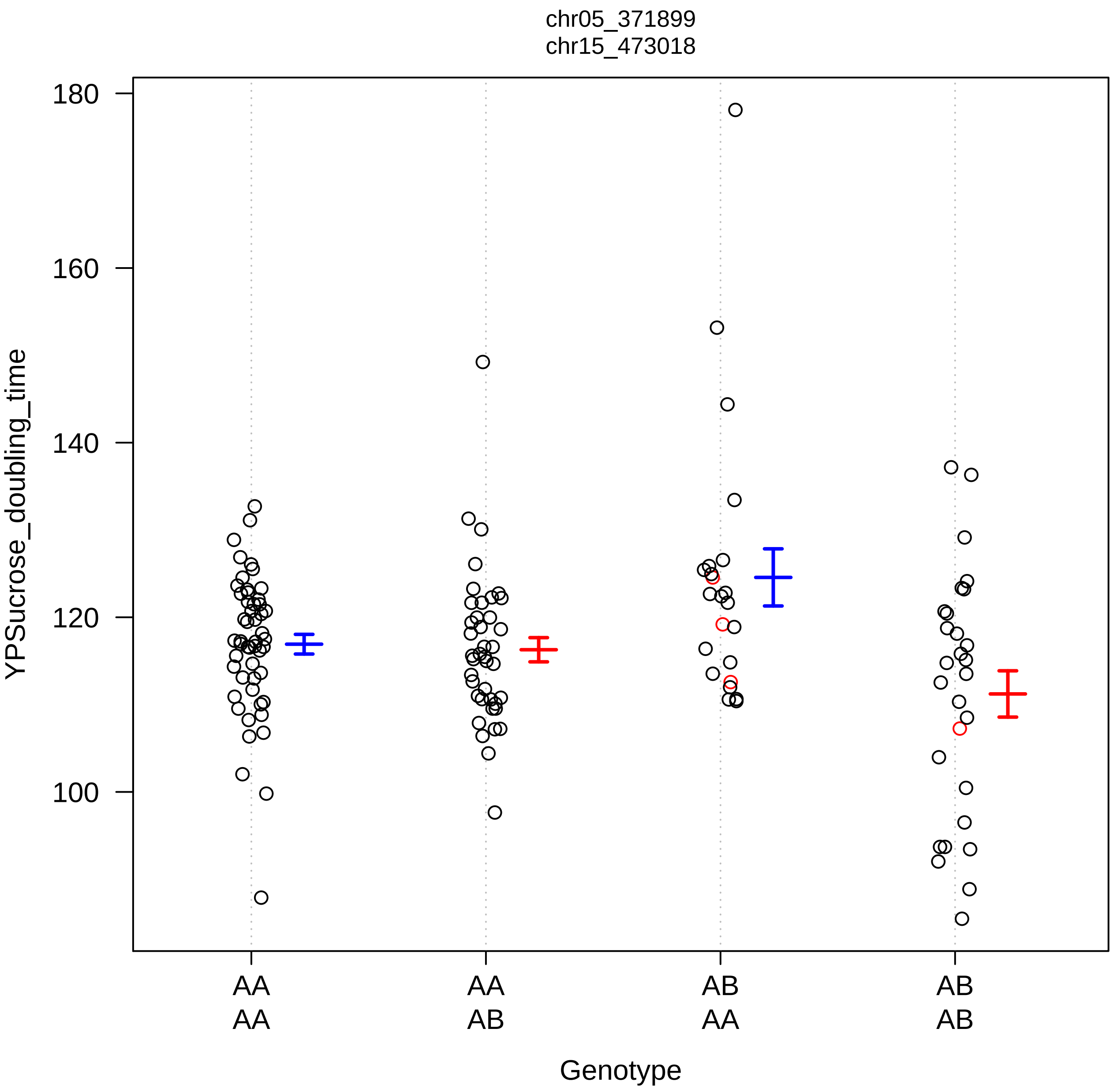
chr05_371899
chr15_473018
YPSucrose_doubling_time
180
160
140
120
100
AA
AA
AA
AB
AB
AA
AB
AB
Genotype
