# Supplementary material for: Yeast Growth Plasticity Is Regulated by Environment-Specific Multi-QTL Interactions
Source: G3 (Bethesda). 2014 Jan 28;4(5):769–77. doi: 10.1534/g3.113.009142 (PMC4025475; doi:10.1534/g3.113.009142)
Supplement: Supporting Information [file supp_g3.113.009142_TableS1.pdf]

Table S1

a. Pearson Correlation Coefficient between various growth media for doubling time (n = 144 segregants).

Condition	Ethanol	Fructose	Glucose	Glycerol	Lactose	Maltose	Sucrose
Ethanol	1.00	0.35	0.38	0.58	0.63	0.25	0.33
Fructose		1.00	0.41	0.33	0.52	0.19	0.53
Glucose			1.00	0.46	0.45	0.32	0.44
Glycerol				1.00	0.59	0.34	0.34
Lactose					1.00	0.40	0.39
Maltose						1.00	0.15
Sucrose							1.00

b. Pearson Correlation Coefficient between various growth media for maxOD (n = 144 segregants).

Condition	Ethanol	Fructose	Glucose	Glycerol	Lactose	Maltose	Sucrose
Ethanol	1.00	0.13	0.11	0.11	0.04	0.01	0.22
Fructose		1.00	0.18	0.30	0.27	0.07	0.12
Glucose			1.00	0.14	-0.02	0.10	0.27
Glycerol				1.00	0.49	0.05	0.11
Lactose					1.00	0.03	0.05
Maltose						1.00	0.19
Sucrose							1.00
