# Supplementary material for: Yeast Growth Plasticity Is Regulated by Environment-Specific Multi-QTL Interactions
Source: G3 (Bethesda). 2014 Jan 28;4(5):769–77. doi: 10.1534/g3.113.009142 (PMC4025475; doi:10.1534/g3.113.009142)
Supplement: Supporting Information [file supp_g3.113.009142_TableS2.pdf]

Broad sense heritability

To calculate broad sense heritability, we used the `lmer` function in the lme4 R package (Bates 2010) to perform an analysis of variance with a random effects model on 2 experimental replicates of segregant data (following procedure described in Poecke *et al.* 2007).

The `confint` function (Bates 2010) was used to compute standard errors of the variance estimates provided by `lmer`, and these errors were then propagated to compute the 95% confidence intervals for the broad sense heritability.

Table S2 Broad sense heritabilities for each growth parameter and environmental condition (parentheses indicate 95% CI).

Condition	Ethanol	Fructose	Glucose	Glycerol	Lactose	Maltose	Sucrose
Doubling time	0.13 (0.07, 0.18)	0.44 (0.32, 0.55)	0.65 (0.59, 0.72)	0.96 (0.95, 0.98)	0.40 (0.29, 0.52)	0.53 (0.46, 0.61)	0.20 (0.03, 0.36)
maxOD	0.04 (0.01, 0.09)	0.41 (0.30, 0.53)	0.15 (0.09, 0.21)	0.36 (0.25, 0.47)	0.14 (0.00, 0.32)	0.70 (0.63, 0.77)	0.72 (0.62, 0.82)

References

Van Poecke, Remco MP, et al. "Natural variation in RPS2-mediated resistance among Arabidopsis accessions: correlation between gene expression profiles and phenotypic responses." The Plant Cell Online 19.12 (2007): 4046-4060.

Bates, Douglas M. "lme4: Mixed-effects modeling with R." URL http://lme4.r-forge.r-project.org/book (2010).

Broman, Karl W., and Saunak Sen. A guide to QTL mapping with R-qtl. Springer, 2009.
