# Supplementary material for: Yeast Growth Plasticity Is Regulated by Environment-Specific Multi-QTL Interactions
Source: G3 (Bethesda). 2014 Jan 28;4(5):769–77. doi: 10.1534/g3.113.009142 (PMC4025475; doi:10.1534/g3.113.009142)
Supplement: Supporting Information [file supp_g3.113.009142_TableS3.pdf]

Percentage of phenotypic variance attributable to single environment QTL

We calculated the fraction of phenotypic variance attributable to single environment QTL using the method described in Broman and Sen (2009). The formula for the percent variance explained by a single QTL is $1-10^{(\frac{-2\times LOD}{n})}$ where LOD is the LOD score of the QTL and n is the number of segregants (n = 144).

Table S3 Total percentage of phenotypic variance attributable to single-environment QTL.

Condition	Ethanol	Fructose	Glucose	Glycerol	Lactose	Maltose	Sucrose
Doubling time	26.2	29.4	24.7	0	40.2	43.2	10.6
maxOD	13.7	11.2	0	0	0	57.6	0
