# Supplementary material for: Yeast Growth Plasticity Is Regulated by Environment-Specific Multi-QTL Interactions
Source: G3 (Bethesda). 2014 Jan 28;4(5):769–77. doi: 10.1534/g3.113.009142 (PMC4025475; doi:10.1534/g3.113.009142)
Supplement: Supporting Information [file supp_g3.113.009142_FileS1.pdf]

File S1

Yeast growth plasticity is regulated by environment specific multi-QTL interactions

Aatish Bhatia, Anupama Yadav, Chenchen Zhu, Julien Gagneur, Aparna Radhakrishnan, Lars M. Steinmetz, Gyan Bhanot and Himanshu Sinha

1 Estimating growth parameters

Doubling time and biomass were robustly estimated from the maximum slope and maximum value respectively of smooth fits of the raw data (local polynomial fit of order 2) using the R/Bioconductor "cellGrowth" package. The bandwidth of the local regression was determined using a ten-fold cross-validation as implemented in the cellGrowth package with default parameters.

Out of the 157 segregants analyzed by Gagneur *et al.* (2013), based on standard error distribution, some segregants showing a large error were filtered out. Thus, in each environment, for mapping QTL, 144 segregants were analyzed. For further details see File S2.

2 Mapping single environment QTL

The R/qtl package (Broman *et al.* 2003, Broman and Sen 2009) was used to construct a genetic map and to identify QTL separately for the each growth parameter in each of the 7 environmental conditions. QTL were identified using the LOD score, which is the of the ratio of the likelihood of the experimental hypothesis to the likelihood of the null hypothesis (Broman and Sen 2009).

For single environment QTL analysis, the data was used to distinguish

the following hypotheses:

$$H_1 : y_i = \mu + \beta g_i + \epsilon_i$$
$$H_0 : y_1 = \mu + \epsilon_i$$

where y_i is the phenotype of strain i, g_i is the genotype variable (0 or 1) and ϵ_i is a random variable with zero mean and fixed variance, representing phenotypic variation. H_1 is the model that the two genotypes have different means (i.e. a QTL is present), and H_0 is the null model that both genotypes have the same mean (no QTL present). The parameter β captures the effect of the QTL.

A likelihood function for each hypothesis can then be defined as:

$$\begin{aligned} L(H_1) &= \prod_i \phi(y_i|\mu + \beta g_i, \sigma^2) \\ L(H_0) &= \prod_i \phi(y_i|\mu, \sigma^2) \end{aligned}$$

where ϕ is the density function for the normal distribution, and the parameters μ, β and σ^2 are obtained for each hypothesis by maximizing the likelihood.

The LOD score of interest for a single environment QTL is then:

$$LOD(H_1) - LOD(H_0) = \log(\frac{L(H_1)}{L(H_0)})$$

An interval mapping method ("scanone" function in R/qtl) was used to compute this LOD score, using the Haley-Knott regression algorithm (Broman *et al.* 2003). This method has the advantage over marker regression in that it can impute data at missing markers and inspect positions between markers. We computed p-values in R/qtl with a permutation test (1,000 permutations) where the null distribution consisted of the highest genome-wide LOD score obtained from each permutation (Broman *et al.* 2003)

3 Mapping GEI

A GEI occurs when the effect of a QTL is environment dependent. We identify such QTL data by pooling data from two environmental conditions

and including the effect of the environment as a covariate. Concretely, we compare the following hypotheses:

$$H_I \;:\; y_i = \mu + \beta_g g_i + \beta_x x_i + \gamma g_i x_i + \epsilon_i$$
$$H_A \;:\; y_i = \mu + \beta_g g_i + \beta_x x_i + \epsilon_i$$

The new variable x_i is an environmental covariate that is 0 or 1 depending on the environment of the strain. As before, the parameters μ, β_g, β_x, γ are all obtained by maximizing the likelihood. In H_A, the effect of the environment is modeled as an additive covariate, i.e. the phenoype is the sum of a constant QTL effect (β_g) and a constant environment dependent effect (β_x). In H_I, the effect of the environment is modeled as an interactive covariate. The term γ captures the effect of the GEI.

To identify a QTL-environment interaction, the LOD score of interest is then $LOD(H_I) - LOD(H_A)$. These scores were calculated using the scanone function in R/qtl (we chose the Haley–Knott regression algorithm) (Broman *et al.* 2003), including the environmental variable as an additive and interactive covariate. P-values were computed with a permutation test in R/qtl (n = 100 permutations).

4 Mapping two-QTL interactions

A two-QTL interaction occurs when the effect of a QTL at a single locus depends on the genotype at some other locus. We identified the presence of two-QTL interactions by comparing the following hypotheses:

$$H_I : y_i = \mu + \beta_1 g_{1i} + \beta_2 g_{2i} + \gamma g_{1i} g_{2i} + \epsilon_i$$
$$H_A : y_i = \mu + \beta_1 g_{1i} + \beta_2 g_{2i} + \epsilon_i$$

Here g_{1i} and g_{2i} are binary variables that specify the genotypes at two loci. As before, μ, β_1, β_2 and γ are inferred from the data using maximum likelihood. The parameters β_1 and β_2 quantify the individual effect of each QTL, and γ quantifies the effect of the two-QTL interaction.

The LOD score of interest in identifying two-QTL interactions is $LOD(H_I)-$

$LOD(H_A)$. The log likelihood of each hypothesis can be written as

$$\log \mathcal{L} = \frac{n}{2} \log \left(\frac{1}{2\pi\sigma^2} \right) - \frac{1}{2\sigma^2} \sum_{j \in \{00,01,10,11\}} \left(\sum_{i \in G_j} (x_i - \bar{x}_j)^2 + n_j (\bar{x}_j - \mu_j)^2 \right) \tag{1}$$

where

$$\begin{aligned} \mu_{00} &= \mu \\ \mu_{10} &= \mu + \beta_1 \\ \mu_{01} &= \mu + \beta_2 \\ \mu_{11} &= \mu + \beta_1 + \beta_2 + \gamma \end{aligned}$$

for the interactive hypothesis H_I.

Maximizing Equation 1 with respect to μ, β_1, β_2 and γ shows that

$$\begin{aligned} \hat{\mu} &= \bar{x}_{00} \\ \hat{\beta}_1 &= \bar{x}_{10} - \hat{\mu} \\ \hat{\beta}_2 &= \bar{x}_{01} - \hat{\mu} \\ \hat{\gamma} &= \bar{x}_{11} - \hat{\mu} - \hat{\beta}_1 - \hat{\beta}_2 \end{aligned}$$

For the additive hypothesis H_A, there is one fewer parameter:

$$\begin{aligned} \mu_{00} &= \mu \\ \mu_{10} &= \mu + \beta_1 \\ \mu_{01} &= \mu + \beta_2 \\ \mu_{11} &= \mu + \beta_1 + \beta_2 \end{aligned}$$

Maximizing Equation 1 with respect to μ, β_1 and β_2 gives a set of three equations:

$$\begin{aligned} n_{00}(\bar{x}_{00} - \mu) + n_{10}(\bar{x}_{10} - \mu - \beta_1) + n_{01}(\bar{x}_{01} - \mu - \beta_2) + n_{11}(\bar{x}_{11} - \mu - \beta_1 - \beta_2) &= 0 \\ n_{10}(\bar{x}_{10} - \mu - \beta_1) + n_{11}(\bar{x}_{11} - \mu - \beta_1 - \beta_2) &= 0 \\ n_{01}(\bar{x}_{01} - \mu - \beta_2) + n_{11}(\bar{x}_{11} - \mu - \beta_1 - \beta_2) &= 0 \end{aligned}$$

Which can be solved to provide maximum likelihood estimates for μ, β_1, and β_2.

$$
\begin{aligned}
\alpha &= n_{01}n_{10}(n_{00}+n_{11}) + n_{00}n_{11}(n_{01}+n_{10}) \\
\hat{\mu} &= (n_{00}(n_{10}n_{11}+n_{01}(n_{10}+n_{11}))\bar{x}_{00} + n_{01}n_{10}n_{11}(\bar{x}_{01}+\bar{x}_{10}-\bar{x}_{11}))/\alpha \\
\hat{\beta}_1 &= ((n_{00}+n_{10})n_{01}n_{11}(\bar{x}_{11}-\bar{x}_{01}) + n_{00}n_{10}(n_{01}+n_{11})(\bar{x}_{10}-\bar{x}_{00}))/\alpha \\
\hat{\beta}_2 &= ((n_{00}+n_{01})n_{10}n_{11}(\bar{x}_{11}-\bar{x}_{10}) + n_{00}n_{01}(n_{10}+n_{11})(\bar{x}_{01}-\bar{x}_{00}))/\alpha
\end{aligned} \tag{2}
$$

The LOD score is then $\log \mathcal{L}(H_I) - \log \mathcal{L}(H_A)$

$$
\begin{aligned}
LOD = \frac{n}{2}\log\Bigg(&\sum_{i\in G_{00}}(x_i-\hat{\mu})^2 + \sum_{i\in G_{10}}(x_i-\hat{\mu}-\hat{\beta}_1)^2 + \sum_{i\in G_{01}}(x_i-\hat{\mu}-\hat{\beta}_2)^2 \\
&+ \sum_{i\in G_{11}}(x_i-\hat{\mu}-\hat{\beta}_1-\hat{\beta}_2)^2\Bigg) - \frac{n}{2}\log\Bigg(\sum_{i\in G_{00}}(x_i-\bar{x}_{00})^2 \\
&+ \sum_{i\in G_{10}}(x_i-\bar{x}_{10})^2 + \sum_{i\in G_{01}}(x_i-\bar{x}_{01})^2 + \sum_{i\in G_{11}}(x_i-\bar{x}_{11})^2\Bigg)
\end{aligned} \tag{3}
$$

where the parameters $\hat{\mu}$, $\hat{\beta}_1$, $\hat{\beta}_2$ are obtained using equation 2.

We used a custom-written python script to compute this LOD score for pairwise comparisons among a set of markers. Our script did not impute missing genotypes. We compute p-values in python with a permutation test (10,000 permutations) where the null distribution consisted of the highest LOD score obtained among all pairwise comparisons for each permutation of the phenotype. To avoid detection of spurious interactions due to the linkage disequilibrium between markers or due to missing data, the permutations with either markers within 10kb or 90% missing (genotype or phenotype) data were not considered.

5 Mapping three-QTL interactions

Thus far we have investigated two locus interactions, where the phenotype data is mapped to a relation of the form

$$y = \mu + \sum_j \beta_g g_j + \sum_{j,k} \gamma_{jk} g_j g_k + \epsilon$$

In the equation above, we have dropped the i subscript on y, g, and ϵ for the sake of clarity. This model accounts for single locus effects as well as pairwise interactions. However, we can also investigate three-point interactions such as QTL $\times$ QTL $\times$ QTL interactions or QTL $\times$ QTL $\times$ environment interactions by investigating the effect of an additional term of the form $\delta g_1 g_2 g_3$ where g_3 could be an additional genotype locus, or it could be an environmental covariate variable x. The term δ captures the effect size of the QTL $\times$ QTL $\times$ environment or QTL $\times$ QTL $\times$ QTL interaction.

Concretely, we compare the following hypotheses:

$$H_{3I} : y_i = \mu + \beta_1 g_{1i} + \beta_2 g_{2i} + \beta_3 g_{3i} + \gamma_{12} g_{1i} g_{2i} + \gamma_{13} g_{1i} g_{3i} + \gamma_{23} g_{2i} g_{3i} + \delta g_{1i} g_{2i} g_{3i} + \epsilon_i$$
$$H_{2I} : y_i = \mu + \beta_1 g_{1i} + \beta_2 g_{2i} + \beta_3 g_{3i} + \gamma_{12} g_{1i} g_{2i} + \gamma_{13} g_{1i} g_{3i} + \gamma_{23} g_{2i} g_{3i} + \epsilon_i$$

As before, the log likelihood of either hypothesis can be written as:

$$\log \mathcal{L} = \frac{n}{2} \log \left(\frac{1}{2\pi\sigma^2} \right) - \frac{1}{2\sigma^2} \sum_{j \in \{\mathcal{Z}_2 \times \mathcal{Z}_2 \times \mathcal{Z}_2\}} \left(\sum_{i \in G_j} (x_i - \bar{x}_j)^2 + n_j (\bar{x}_j - \mu_j)^2 \right)$$

Maximizing $\log \mathcal{L}(H_{3I})$ gives us the maximum likelihood estimate of the parameters of H_{3I}:

$$\begin{aligned}
\hat{\mu} &= \bar{x}_{000} \\
\hat{\beta}_1 &= \bar{x}_{100} - \hat{\mu} \\
\hat{\beta}_2 &= \bar{x}_{010} - \hat{\mu} \\
\hat{\beta}_3 &= \bar{x}_{001} - \hat{\mu} \\
\hat{\gamma}_{12} &= \bar{x}_{110} - \hat{\beta}_1 - \hat{\beta}_2 - \hat{\mu} \\
\hat{\gamma}_{23} &= \bar{x}_{011} - \hat{\beta}_2 - \hat{\beta}_3 - \hat{\mu} \\
\hat{\gamma}_{13} &= \bar{x}_{101} - \hat{\beta}_1 - \hat{\beta}_3 - \hat{\mu} \\
\hat{\delta} &= \bar{x}_{111} - \hat{\gamma}_{12} - \hat{\gamma}_{23} - \hat{\gamma}_{13} - \hat{\beta}_1 - \hat{\beta}_2 - \hat{\beta}_3 - \hat{\mu}
\end{aligned}$$

To maximize the likelihood of H_{2I}, we differentiate $\log \mathcal{L}(H_{2I})$ with respect to μ, β_1, β_2, β_3, γ_{12}, γ_{23}, γ_{13}, resulting in a set of 7 simultaneous linear equations:

$$\begin{aligned}
&2n_{000}(\bar{x}_{000} - \mu) + 2n_{100}(\bar{x}_{100} - \beta_1 - \mu) + 2n_{010}(\bar{x}_{010} - \beta_2 - \mu) \\
&\quad + 2n_{001}(\bar{x}_{001} - \beta_3 - \mu) + 2n_{110}(\bar{x}_{110} - \beta_1 - \beta_2 - \gamma_{12} - \mu) \\
&\quad + 2n_{101}(\bar{x}_{101} - \beta_1 - \beta_3 - \gamma_{13} - \mu) + 2n_{011}(\bar{x}_{011} - \beta_2 - \beta_3 - \gamma_{23} - \mu) \\
&\quad + 2n_{111}(\bar{x}_{111} - \beta_1 - \beta_2 - \beta_3 - \gamma_{12} - \gamma_{13} - \gamma_{23} - \mu) = 0
\end{aligned}$$

$$
\begin{aligned}
&2n_{100}(\bar{x}_{100} - \beta_1 - \mu) + 2n_{110}(\bar{x}_{110} - \beta_1 - \beta_2 - \gamma_{12} - \mu) \\
&\quad + 2n_{101}(\bar{x}_{101} - \beta_1 - \beta_3 - \gamma\gamma_{13} - \mu) \\
&\quad + 2n_{111}(\bar{x}_{111} - \beta_1 - \beta_2 - \beta_3 - \gamma_{12} - \gamma_{13} - \gamma_{23} - \mu) = 0
\end{aligned}
$$

$$
\begin{aligned}
&2n_{010}(\bar{x}_{010} - \beta_2 - \mu) + 2n_{110}(\bar{x}_{110} - \beta_1 - \beta_2 - \gamma_{12} - \mu) \\
&\quad + 2n_{011}(\bar{x}_{011} - \beta_2 - \beta_3 - \gamma_{23} - \mu) \\
&\quad + 2n_{111}(\bar{x}_{111} - \beta_1 - \beta_2 - \beta_3 - \gamma_{12} - \gamma_{13} - \gamma_{23} - \mu) = 0
\end{aligned}
$$

$$
\begin{aligned}
&2n_{001}(\bar{x}_{001} - \beta_3 - \mu) + 2n_{101}(\bar{x}_{101} - \beta_1 - \beta_3 - \gamma_{13} - \mu) \\
&\quad + 2n_{011}(\bar{x}_{011} - \beta_2 - \beta_3 - \gamma_{23} - \mu) \\
&\quad + 2n_{111}(\bar{x}_{111} - \beta_1 - \beta_2 - \beta_3 - \gamma_{12} - \gamma_{13} - \gamma_{23} - \mu) = 0
\end{aligned}
$$

$$
\begin{aligned}
&2n_{110}(\bar{x}_{110} - \beta_1 - \beta_2 - \gamma_{12} - \mu) \\
&\quad + 2n_{111}(\bar{x}_{111} - \beta_1 - \beta_2 - \beta_3 - \gamma_{12} - \gamma_{13} - \gamma_{23} - \mu) = 0
\end{aligned}
$$

$$
\begin{aligned}
&2n_{011}(\bar{x}_{011} - \beta_2 - \beta_3 - \gamma_{23} - \mu) \\
&\quad + 2n_{111}(\bar{x}_{111} - \beta_1 - \beta_2 - \beta_3 - \gamma_{12} - \gamma_{13} - \gamma_{23} - \mu) = 0
\end{aligned}
$$

$$
\begin{aligned}
&2n_{101}(\bar{x}_{101} - \beta_1 - \beta_3 - \gamma_{13} - \mu) \\
&\quad + 2n_{111}(\bar{x}_{111} - \beta_1 - \beta_2 - \beta_3 - \gamma_{12} - \gamma_{13} - \gamma_{23} - \mu) = 0
\end{aligned}
$$

The solution to these simultaneous equations gives us the maximum likelihood estimates $\hat{\mu}$, $\hat{\beta}_1$, $\hat{\beta}_2$, $\hat{\beta}_3$, $\hat{\gamma}_{12}$, $\hat{\gamma}_{23}$, $\hat{\gamma}_{13}$

$$
\begin{aligned}
\alpha = (&n_{000}n_{001}n_{010}n_{011}n_{100}n_{101}n_{110} + n_{001}n_{010}n_{011}n_{100}n_{101}n_{110}n_{111} \\
&+ n_{000}(n_{001}n_{010}n_{011}n_{100}n_{101} + n_{010}n_{011}n_{100}n_{101}n_{110} \\
&+ n_{001}(n_{010}n_{011}n_{100} + n_{011}n_{100}n_{101} + n_{010}(n_{011} + n_{100})n_{101})n_{110})n_{111})
\end{aligned}
$$

$$
\begin{aligned}
\hat{\mu} \cdot \alpha = n_{000}(&n_{001}n_{010}n_{011}n_{100}n_{101}n_{110} + n_{010}n_{011}n_{100}n_{101}n_{110}n_{111} \\
&+ n_{001}(n_{010}n_{011}n_{100}n_{101} \\
&+ (n_{010}n_{011}n_{100} + n_{011}n_{100}n_{101} + n_{010}(n_{011} + n_{100})n_{101})n_{110})n_{111})\bar{x}_{000} \\
&+ n_{001}n_{010}n_{011}n_{100}n_{101}n_{110}n_{111}(\bar{x}_{001} + \bar{x}_{010} - \bar{x}_{011} + \bar{x}_{100} - \bar{x}_{101} - \bar{x}_{110} \\
&+ \bar{x}_{111})
\end{aligned}
$$

$$
\begin{aligned}
\hat{\beta}_1 \cdot \alpha = {} & n_{000}(n_{010}n_{011}n_{100}n_{101}n_{110}n_{111}(-\bar{x}_{000} + \bar{x}_{100}) \\
& + n_{001}(n_{011}n_{100}n_{101}n_{110}n_{111}(-\bar{x}_{000} + \bar{x}_{100}) \\
& + n_{010}(n_{100}n_{101}n_{110}n_{111}(-\bar{x}_{000} + \bar{x}_{100}) \\
& + n_{011}(-n_{100}(n_{101}n_{110} + (n_{101} + n_{110})n_{111})(\bar{x}_{000} - \bar{x}_{100}) \\
& + n_{101}n_{110}n_{111}(-\bar{x}_{001} - \bar{x}_{010} + \bar{x}_{011} + \bar{x}_{101} + \bar{x}_{110} - \bar{x}_{111}))))) \\
& + n_{001}n_{010}n_{011}n_{100}n_{101}n_{110}n_{111}(-\bar{x}_{001} - \bar{x}_{010} + \bar{x}_{011} + \bar{x}_{101} + \bar{x}_{110} - \bar{x}_{111})
\end{aligned}
$$

$$
\begin{aligned}
\hat{\beta}_2 \cdot \alpha = {} & n_{000}(n_{010}n_{011}n_{100}n_{101}n_{110}n_{111}(-\bar{x}_{000} + \bar{x}_{010}) \\
& + n_{001}(-n_{010}(n_{011}n_{100}n_{101}n_{110} + (n_{011}n_{100}n_{101} + n_{100}n_{101}n_{110} \\
& + n_{011}(n_{100} + n_{101})n_{110})n_{111})(\bar{x}_{000} - \bar{x}_{010}) \\
& + n_{011}n_{100}n_{101}n_{110}n_{111}(-\bar{x}_{001} + \bar{x}_{011} - \bar{x}_{100} + \bar{x}_{101} + \bar{x}_{110} - \bar{x}_{111}))) \\
& + n_{001}n_{010}n_{011}n_{100}n_{101}n_{110}n_{111}(-\bar{x}_{001} + \bar{x}_{011} - \bar{x}_{100} + \bar{x}_{101} + \bar{x}_{110} - \bar{x}_{111})
\end{aligned}
$$

$$
\begin{aligned}
\hat{\beta}_3 \cdot \alpha = {} & n_{000}(-n_{001}(n_{010}n_{011}n_{100}n_{101}n_{110} + n_{011}n_{100}n_{101}n_{110}n_{111} \\
& + n_{010}(n_{011}n_{100}n_{101} + n_{100}n_{101}n_{110} + n_{011}(n_{100} + n_{101})n_{110})n_{111})(\bar{x}_{000} \\
& - \bar{x}_{001}) \\
& + n_{010}n_{011}n_{100}n_{101}n_{110}n_{111}(-\bar{x}_{010} + \bar{x}_{011} - \bar{x}_{100} + \bar{x}_{101} + \bar{x}_{110} - \bar{x}_{111})) \\
& + n_{001}n_{010}n_{011}n_{100}n_{101}n_{110}n_{111}(-\bar{x}_{010} + \bar{x}_{011} - \bar{x}_{100} + \bar{x}_{101} + \bar{x}_{110} - \bar{x}_{111})
\end{aligned}
$$

$$
\begin{aligned}
\hat{\gamma}_{12} \cdot \alpha = {} & n_{001}n_{010}n_{011}n_{100}n_{101}n_{110}n_{111}(\bar{x}_{001} - \bar{x}_{011} - \bar{x}_{101} + \bar{x}_{111}) \\
& + n_{000}(n_{010}n_{011}n_{100}n_{101}n_{110}n_{111}(\bar{x}_{000} - \bar{x}_{010} - \bar{x}_{100} + \bar{x}_{110}) \\
& + n_{001}(n_{011}n_{100}n_{101}n_{110}n_{111}(\bar{x}_{001} - \bar{x}_{011} - \bar{x}_{101} + \bar{x}_{111}) \\
& + n_{010}(n_{100}n_{101}n_{110}n_{111}(\bar{x}_{000} - \bar{x}_{010} - \bar{x}_{100} + \bar{x}_{110}) \\
& + n_{011}(n_{101}n_{110}n_{111}(\bar{x}_{001} - \bar{x}_{011} - \bar{x}_{101} + \bar{x}_{111}) + n_{100}(n_{110}n_{111}(\bar{x}_{000} - \bar{x}_{010} - \bar{x}_{100} + \bar{x}_{110}) \\
& + n_{101}(n_{110}(\bar{x}_{000} - \bar{x}_{010} - \bar{x}_{100} + \bar{x}_{110}) + n_{111}(\bar{x}_{001} - \bar{x}_{011} - \bar{x}_{101} + \bar{x}_{111})))))))
\end{aligned}
$$

$$
\begin{aligned}
\hat{\gamma}_{23} \cdot \alpha = {} & n_{001}n_{010}n_{011}n_{100}n_{101}n_{110}n_{111}(\bar{x}_{100} - \bar{x}_{101} - \bar{x}_{110} + \bar{x}_{111}) \\
& + n_{000}(n_{010}n_{011}n_{100}n_{101}n_{110}n_{111}(\bar{x}_{100} - \bar{x}_{101} - \bar{x}_{110} + \bar{x}_{111}) \\
& + n_{001}(n_{011}n_{100}n_{101}n_{110}n_{111}(\bar{x}_{100} - \bar{x}_{101} - \bar{x}_{110} + \bar{x}_{111}) \\
& + n_{010}(n_{011}(n_{100}n_{101}n_{110} + n_{101}n_{110}n_{111} + n_{100}(n_{101} + n_{110})n_{111}) \\
& (\bar{x}_{000} - \bar{x}_{001} - \bar{x}_{010} + \bar{x}_{011}) + n_{100}n_{101}n_{110}n_{111}(\bar{x}_{100} - \bar{x}_{101} - \bar{x}_{110} + \bar{x}_{111}))))
\end{aligned}
$$

$$
\begin{aligned}
\hat{\gamma}_{13} \cdot \alpha = {} & n_{001}n_{010}n_{011}n_{100}n_{101}n_{110}n_{111}(\bar{x}_{010} - \bar{x}_{011} - \bar{x}_{110} + \bar{x}_{111}) \\
& + n_{000}(n_{010}n_{011}n_{100}n_{101}n_{110}n_{111}(\bar{x}_{010} - \bar{x}_{011} - \bar{x}_{110} + \bar{x}_{111}) \\
& + n_{001}(n_{011}n_{100}n_{101}n_{110}n_{111}(\bar{x}_{000} - \bar{x}_{001} - \bar{x}_{100} + \bar{x}_{101}) \\
& + n_{010}(n_{100}n_{101}n_{110}n_{111}(\bar{x}_{000} - \bar{x}_{001} - \bar{x}_{100} + \bar{x}_{101}) \\
& + n_{011}(n_{101}n_{110}n_{111}(\bar{x}_{010} - \bar{x}_{011} - \bar{x}_{110} + \bar{x}_{111}) \\
& + n_{100}(n_{101}(n_{110} + n_{111})(\bar{x}_{000} - \bar{x}_{001} - \bar{x}_{100} + \bar{x}_{101}) + n_{110}n_{111}(\bar{x}_{010} - \bar{x}_{011} - \bar{x}_{110} + \bar{x}_{111}))))))
\end{aligned}
$$

The LOD score is then $\log \mathcal{L}(H_{3I}) - \log \mathcal{L}(H_{2I})$

$$\begin{aligned}
LOD = \frac{n}{2} \log \Bigg(&\sum_{i \in G_{000}} (x_i - \hat{\mu})^2 + \sum_{i \in G_{100}} (x_i - \hat{\mu} - \hat{\beta}_1)^2 + \sum_{i \in G_{010}} (x_i - \hat{\mu} - \hat{\beta}_2)^2 \\
&+ \sum_{i \in G_{001}} (x_i - \hat{\mu} - \hat{\beta}_3)^2 + \sum_{i \in G_{110}} (x_i - \hat{\mu} - \hat{\beta}_1 - \hat{\beta}_2 - \hat{\gamma}_{12})^2 \\
&+ \sum_{i \in G_{011}} (x_i - \hat{\mu} - \hat{\beta}_2 - \hat{\beta}_3 - \hat{\gamma}_{23})^2 + \sum_{i \in G_{101}} (x_i - \hat{\mu} - \hat{\beta}_1 - \hat{\beta}_3 - \hat{\gamma}_{13})^2 \\
&+ \sum_{i \in G_{111}} (x_i - \hat{\mu} - \hat{\beta}_1 - \hat{\beta}_2 - \hat{\beta}_2 - \hat{\gamma}_{12} - \hat{\gamma}_{23} - \hat{\gamma}_{13})^2 \Bigg) \\
- \frac{n}{2} \log \Bigg(&\sum_{i \in G_{000}} (x_i - \bar{x}_{000})^2 + \sum_{i \in G_{100}} (x_i - \bar{x}_{100})^2 + \sum_{i \in G_{010}} (x_i - \bar{x}_{010})^2 \\
&+ \sum_{i \in G_{001}} (x_i - \bar{x}_{001})^2 + \sum_{i \in G_{110}} (x_i - \bar{x}_{110})^2 + \sum_{i \in G_{011}} (x_i - \bar{x}_{011})^2 \\
&+ \sum_{i \in G_{101}} (x_i - \bar{x}_{101})^2 + \sum_{i \in G_{111}} (x_i - \bar{x}_{111})^2 \Bigg)
\end{aligned} \tag{4}$$

This LOD score can then be used to investigate the presence of QTL × QTL × QTL interactions or QTL × QTL × environment interactions.

6 References

Broman, K. W., H. Wu, S. Sen, and G. A. Churchill, (2003) R/qtl: QTL mapping in experimental crosses. Bioinformatics (Oxford, England) 19: 889–890.

Broman, K. W., and S. Sen, (2009) A Guide to QTL Mapping with R/qtl. Spinger, New York.

Gagneur, J., *et al.*, (2013) Genotype-environment interactions reveal causal pathways that mediate genetic effects on phenotype. PLoS Genet. 9: e1003803.
